# Marine biodiversity was greater in the presence of ecosystem engineers throughout the Phanerozoic

**DOI:** 10.1038/s41467-026-73628-6

**Published:** 2026-07-28

**Authors:** Alison T. Cribb, Simon A. F. Darroch, William Gearty

**Affiliations:** 1 University of Southampton, School of Ocean and Earth Science, Southampton England, UK; 2 Senckenberg Research Institute and Natural History Museum Frankfurt, Frankfurt, Germany; 3 Cluster of Excellence (EXC 3121): TERRA - Terrestrial Geo-Biosphere Interactions in a Changing World. University of Tübingen, Tübingen, Germany; 4 Division of Paleontology, American Museum of Natural History, New York, NY USA; 5 Open Source Program Office, Syracuse University, Syracuse, New York, NY USA

**Keywords:** Palaeoecology, Evolutionary ecology

## Abstract

Phanerozoic marine biodiversity dynamics have been shaped by continuous environmental change and biotic interactions. Although ecosystem engineers – animals whose behaviours modify resource availability – have established impacts on modern community ecology and diversity, their impacts on ancient ecosystems over geologic time have remained quantitatively underexplored. Here, we investigate how biodiversity was impacted by reef-building or bioturbating ecosystem engineers at each stage of the Phanerozoic, including their ability to maintain biodiversity over mass extinctions, and identify extrinsic conditions that modulate their impacts. We analyse fossil communities with and without ecosystem engineers to calculate effect sizes, thereby quantifying the effects of the presence of ecosystem engineers on Shannon’s Diversity (*H*) at each stage in the Phanerozoic. We show that ecosystem engineers are significantly associated with increased biodiversity during the majority of the Phanerozoic. We also show that, over the last ~250 million years, the effectiveness of modern-type reef-builders is more sensitive to climate-driven stress than for modern-style bioturbators. Finally, our results reveal that climate may modulate ecosystem engineer impacts, with both bioturbators and reef-builders exhibiting the strongest effect sizes within an optimal moderate temperature range. These results underscore ecosystem engineers’ crucial role in contributing to fluctuating biodiversity dynamics over the Phanerozoic.

## Abstract (web summary)

Ecosystem engineering by animals is fundamental to our modern biosphere, but the importance of ancient biodiversity is less understood. Here, the authors show that two key groups of marine ecosystem engineers are strongly associated with increased biodiversity over the last ~541 million years.

## Introduction

What drives marine biodiversity dynamics over the Phanerozoic (~538 Ma – present) has long been a major focus of palaeobiology^1–3^. With global datasets such as the Paleobiology Database^2^ and new statistical treatments of the fossil record^4^, two broad key observations of the marine Phanerozoic biodiversity record have emerged: diversity over time has been subject to numerous rises and falls, and diversity appears to have been increasing^2,3^. Since early endeavours to document the history of marine biodiversity^5^, these observations have been subject to a long history of scrutiny, including the demonstration of the impacts of spatial sampling biases^6^, the effects of changing stratigraphic completeness^7^, and the extent to which the observed increase in biodiversity is an artefact of the “Pull of the Recent”^8,9^. Taking the scrutinized reconstructions of ancient biodiversity dynamics as true biological signals, much of this dynamism has been attributed to interactions between life and the abiotic environment, such as biosphere responses to changes in global temperatures^10^, oxygen levels^11^, continental configuration^12^, and sea level^13^. Climate change, for example, is strongly linked to macroevolutionary dynamics^14^, most dramatically during mass extinctions and their subsequent recovery intervals^15^. Increases in biodiversity have also been linked to biotic interactions, such as the case for the predator-prey arms races that are proposed to have caused fundamental step-wise shifts in the structure of marine ecosystems during the early Palaeozoic^16^ and Mesozoic^17^. However, other biotic interactions that are known to have profound impacts on community-level biodiversity today, such as ecosystem engineering^18,19^, have historically been underappreciated as drivers of Phanerozoic biodiversity. Moreover, as recent advances have underscored the importance of local- and regional-scale biotic changes in driving global Phanerozoic biodiversity trends^6,20^, there is a need to understand the importance and impacts of these key biotic interactions.

Ecosystem engineers significantly impact local-scale biodiversity today^18^. While all organisms interact with their environments in some way, ecosystem engineers are organisms whose activities change their environments’ physical characteristics and resource availability for other organisms with which they co-exist^18^. Ecosystem engineers most directly impact ecology by modulating the abiotic controls on community structure and composition^21^. By changing the availability and distribution of resources in their environments, ecosystem engineers impact taxonomic richness^19^, other biotic and trophic interactions^22^, and community resilience to environmental perturbations^23^. Moreover, by changing their environments’ physical characteristics and resource distribution, ecosystem engineers create, modify, and destroy ecological niches – the availability of which is predicted to have had major influences on both biotic radiations and biodiversity on geologic timescales^24^.

In this work, we investigate the role that marine bioturbators and reef-building metazoans – two of the most important ecosystem engineering groups in modern oceans^18^ – have had on biodiversity, providing the first quantitative test of the hypothesis that ecosystem engineers over the Phanerozoic acted as key drivers in increasing biodiversity^24^. Using the Paleobiology Database (paleobiodb.org), we divide the fossil record into palaeocommunities that do and do not contain either bioturbating or reef-building ecosystem engineers. Then, for each Phanerozoic stage, we calculate genera-level Shannon’s Diversity (*H*)^25^ in each formation. Finally, we use the Hedges’ g effect size statistic^26^ to compare *H* in formations with and without ecosystem engineering. These effect sizes allow us to quantify the strength of the impact of the presence of ecosystem engineers on diversity at each stage of the Phanerozoic. Finally, we test whether variability in effect sizes can be explained by either global climate or diversity of ecosystem engineering taxa to understand what controls ecosystem engineering strength throughout the Phanerozoic.

We make three key predictions about the impacts of ecosystem engineers on Phanerozoic biodiversity. First, we predict that diversity will be greater where ecosystem engineers are present, as ecosystem engineers both increase resource availability and the heterogeneity of the distribution of those resources^27,28^. This prediction follows studies of modern ecosystem engineers that show they are associated with greater taxonomic diversity^19,29,30^, and testing it allows us to understand the role the ecosystem engineers played in increasing Phanerozoic biodiversity^2,3^. Second, we predict that positive effect sizes will persist through mass extinction events. While many of the Big 5 Phanerozoic mass extinction events^31^ are associated with severe impacts to both bioturbators^32^ and reef-builders^33^, surviving ecosystem engineers are hypothesized to have been able to ameliorate environmental stress and create refugia where biodiversity remained high^23,34–36^. Surviving ecosystem engineers may strongly influence the structure of communities that survive and recover in the wake of mass extinction events, thus influencing the nature of the rises and falls that are characteristic of the Phanerozoic biodiversity record^2,3^. Finally, we predict that ecosystem engineering impacts should be most strongly correlated with climate, such that the influence of ecosystem engineers is weakened by climatic extremes that already deplete resource availability and stifle biodiversity.

Here, we demonstrate consistently higher local biodiversity where bioturbating and reef-building reef builders are present compared to where they are not. While these strong positive effect sizes arise early and can be found throughout the Phanerozoic, some time intervals demonstrate stronger associations between biodiversity and ecosystem engineers than others. Although we find inconsistent trends across mass extinctions and weak associations between ecosystem engineer diversity and their relationship with biodiversity. Instead, we do find a relationship between climate and ecosystem engineer impacts, in which global climatic extremes are associated with a collapse of the relationships between the presence of ecosystem engineers and greater local biodiversity. Overall, we conclude that the impacts of bioturbating and reef-building ecosystem engineers on local biodiversity are a key part of understanding global biodiversity trends through geologic time.

## Results

**Fig. 1:**
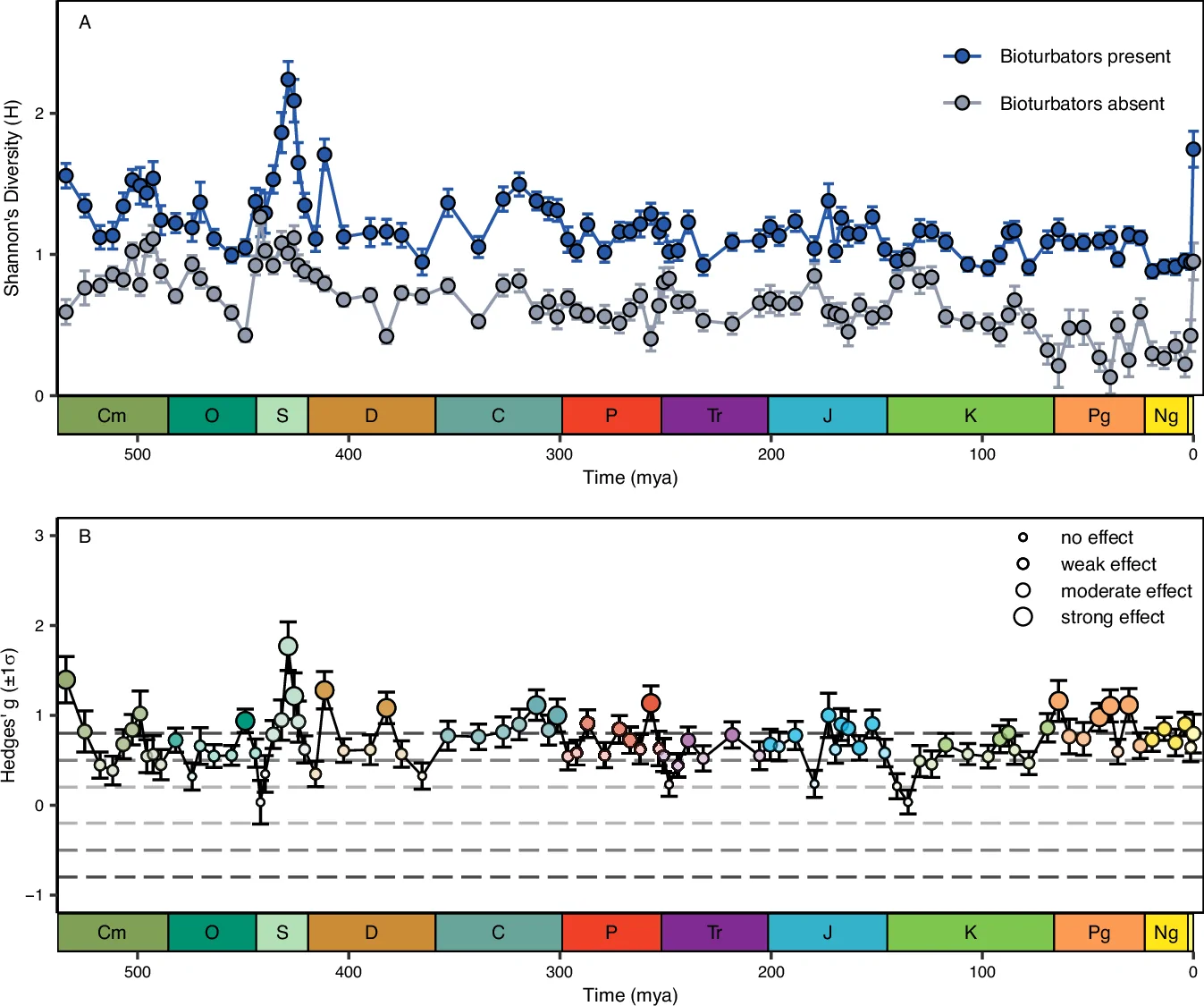
Effects of bioturbators on Shannon’s Diversity (*H*). **A** Each point represents the average *H* taken across all formations where bioturbators are present (blue) or absent (grey). Points are mean *H* of 1000 stage-level subsampling iterations and error bars are ±1 standard deviation. **B** Hedges’ g effect sizes for each stage, where points are colour-coded to their respective geologic periods and size is scaled for effect size strength. Points are mean *g* of 1000 iterations and error bars are ±1 standard deviation. Dashed lines visualize effect size thresholds: the weak effect threshold is at the lightest grey dashed line (±0.2), the moderate effects threshold is at the medium grey dashed line (±0.5), and the strongest effect threshold is at the darkest grey dashed line (±0.8). Geologic timescale abbreviations are: ‘Cm’ = Cambrian, ‘O’ = Ordovician, ‘S’ = Silurian, ‘D’ = Devonian, ‘C’ = Carboniferous, ‘P’ = Permian, ‘Tr’ = Triassic, ‘J’ = Jurassic, ‘K’ = Cretaceous, ‘Pg’ = Paleogene, ‘Ng’ = Neogene. The Quaternary is represented by the final two points on each figure, but is too small to display on the x-axis. Geologic timescale given by ref. ^61^.

In the following section, we describe the effects of bioturbation and reef-building organisms on biodiversity separately, and subsequently the trajectory of effect sizes over mass extinction events, and finally relationships with putative ecological and environmental correlates. Overall, we find positive associations between biodiversity and the presence of both bioturbating and reef-building ecosystem engineers throughout the Phanerozoic (Fig. 1). These differences between formations with and without ecosystem engineers cannot be explained by occurrences of engineering taxa alone (Supplementary Fig. 1). Moreover, while we find some differences in the overall trends of effect sizes through the Phanerozoic among different palaeoenvironments, positive effect sizes are still present when accounting for palaeoenvironmental differences. Although palaeoenvironments may modulate the magnitude of ecosystem engineering effects, we cannot attribute positive impacts on biodiversity to lithology or bathymetry alone (Supplementary Figs. 2–5).

### Effects of bioturbation on biodiversity

Formations where bioturbators are present consistently have higher *H* than formations where bioturbators are absent (Fig. 1A; Supplementary Table 1), driving positive effect sizes throughout the entire interval (Fig. 1B; Supplementary Table 2). While the magnitude of these effect sizes tends to be more variable when constrained within equivalent palaeoenvironments, and particularly when constrained bathymetrically, significant positive effect sizes remain (Supplementary Fig. 3). These positive effect sizes (with non-overlapping uncertainty bounds, strong = *g* ≥ 0.8; moderate = *g* ≥ 0.5; weak = *g* ≥ 0.2) arise early in the Cambrian, and persist subsequently. Within the post-Cambrian Phanerozoic, however, there exist several notable peaks – particularly in the Palaeozoic – in which effect sizes are strong (*g* ≥ 0.8), and significantly higher than in the previous stage (i.e., non-overlapping uncertainty). These peaks occur in the Katian (late Ordovician, ~453–445 Ma), Homerian (Silurian, ~431–427), Pragian and Givetian (both Devonian, ~413–410 Ma and ~388–382 Ma, respectively), Wuchiapingian (Permian, ~259-254 Ma) and Paleogene, ~66–62 Ma). We note that the Fortunian (basal Cambrian, ~539–529) also has a significantly strong effect size that may also represent a peak, but insufficient Ediacaran data is available to calculate a comparable effect size.

There are a minority of stages when bioturbators have insignificant effects on generic richness, notably during the Rhuddanian (Silurian, ~443–441 Ma), Olenekian (Triassic, ~250–247 Ma), Toarcian (Jurassic, ~184–175 Ma) and Berriasian and Valanginian (both Cretaceous, ~143–137 Ma and ~137–133 Ma respectively; Fig. 1B; Supplementary Table 2).

### Effects of reef-building on biodiversity

**Fig. 2:**
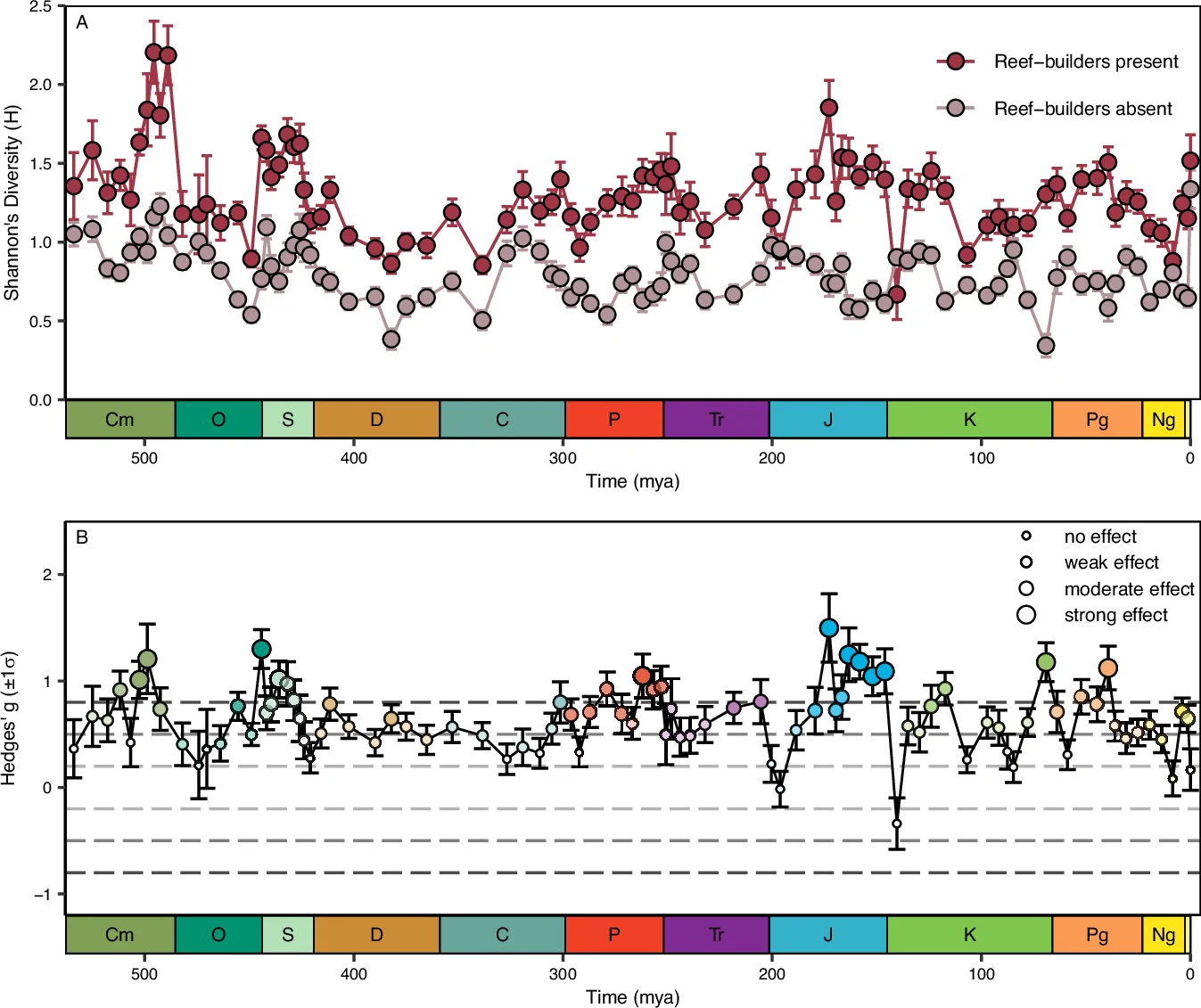
Effects of reef-builders on Shannon’s Diversity (*H*). **A** Each point represents the average *H* taken across all formations where reef-builders are present (pink) or absent (brown). Points are mean *H* of 1000 stage-level subsampling iterations and error bars are ±1 standard deviation. Points not connected by lines represent stages where fewer than two reef communities are present. **B** Hedges’ g effect sizes for each stage, where points are colour-coded to their respective geologic periods and size is scaled for effect size strength. Points are mean *g* of 1000 iterations and error bars are ±1 standard deviation. Effect size thresholds and geologic timescale abbreviations are the same here as for Fig. 1.

Effect sizes associated with reef-building metazoans are more variable than with bioturbators, especially post-Palaeozoic. Similarly, however, there are peaks in effect size strength, in this case during the Drumian and Guzhangian (both Cambrian, ~504–500 Ma and ~500–497 Ma respectively), Hirnantian (Ordovician, ~445–443 Ma), Capitanian (Permian, ~264–259 Ma), Aalenian (Triassic, ~175–171 Ma), Maastrichtian (Cretaceous, ~72–66 Ma), and Bartonian (Paleogene, ~41–37 Ma; Fig. 2B; Supplementary Table 4). These latter intervals of strong positive effect sizes are bracketed by marked collapses in effect sizes in the Mesozoic and Cenozoic. Specifically, effect sizes are insignificant or even weakly negative during the Hettangian and Sinemurian (early Jurassic, ~201–196 Ma and ~199–193 Ma, respectively), Berriasian and Albian (both Cretaceous, ~143–137 Ma and ~113–100 Ma, respectively), and the Santonian (Upper Miocene, ~11–5 Ma) (Supplementary Table 4).

Notably, there are no strong positive effect sizes for the remainder of the Cenozoic following the strong positive peak during the Bartonian. As with bioturbators, effect sizes can be more dynamic in specific palaeoenvironments (notably in only deep marine facies), but strong positive effect sizes remain (Supplementary Fig. 5).

### Effects during mass extinctions

**Fig. 3:**
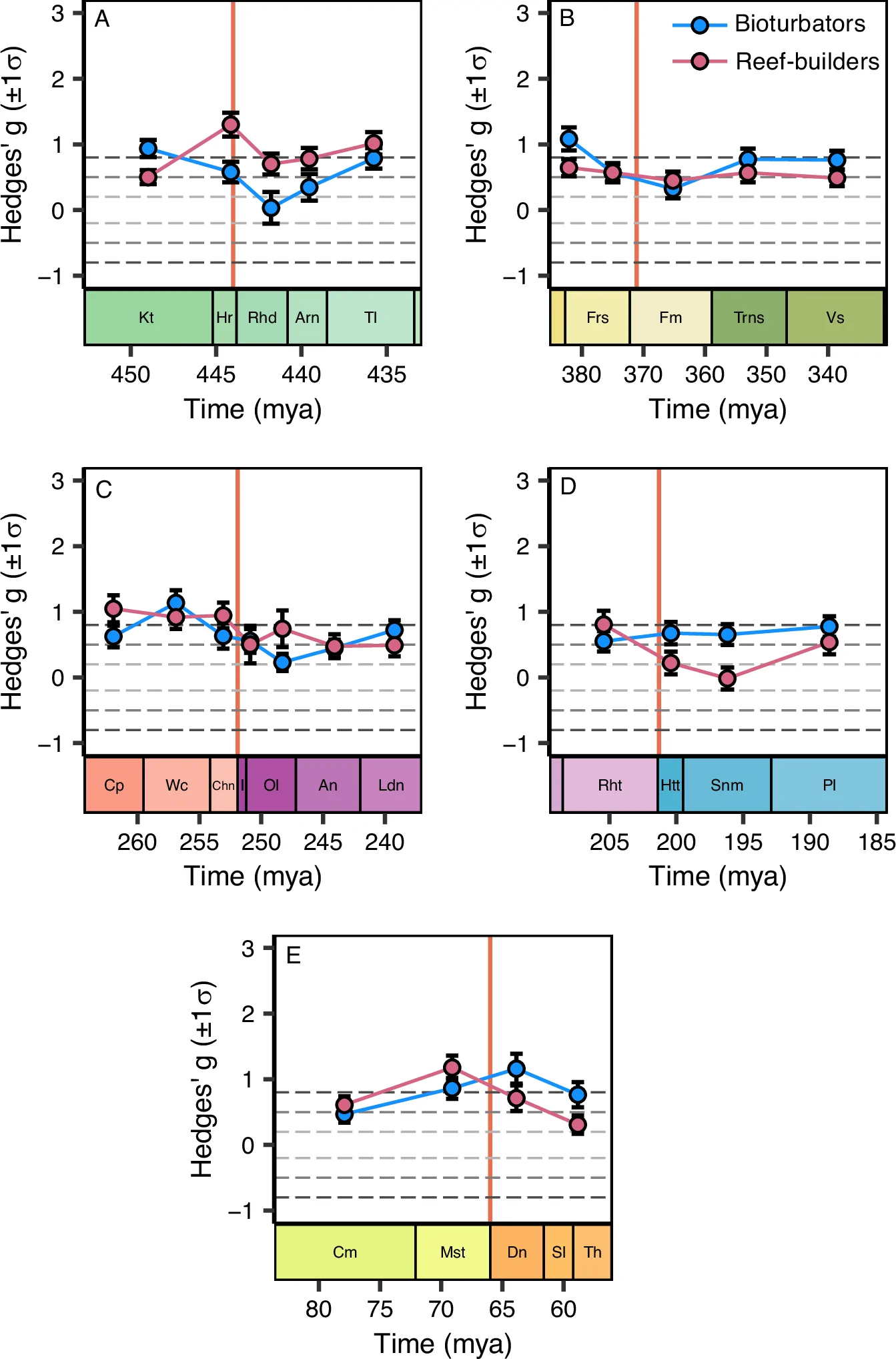
Effect sizes during mass extinctions. Subset of effect sizes from Fig. 1 and Fig. 2 across the major Phanerozoic mass extinction events: **A** the end-Ordovician mass extinction (~444 Ma), **B** the late Devonian mass extinction (~371 Ma), **C** the end-Permian mass extinction (~252 Ma), **D** the end-Triassic mass extinction (~201 Ma), and **E** the Cretaceous-Paleogene mass extinction (~65 Ma). For all panels, blue points and lines represent effect sizes for bioturbators, and pink points and lines represent effect sizes for reef-builders. Estimated mass extinction boundaries are visualized as red vertical lines on each panel. Effect size thresholds are visualized as dashed horizontal lines following Figs. 1 and 2. Points are mean *g* of 1000 stage-level subsampling iterations and error bars are mean ± 1 standard deviation. Geologic timescale abbreviations are as follows: (A) ‘Kt’ = Katian, ‘Hr’ = Hirnantian, ‘Rhd’ = Rhuddanian, ‘Arn’ = Aeronian, ‘Tl’ = Telychian; (B) ‘Frs’ = Frasnian, ‘Fm’ = Famennian, ‘Trns’ = Tournaisian, ‘Vs’ = Visean; (C) ‘Cp’ = Capitanian’, ‘Wc’ = Wuchiapingian, ‘Chn’ = Changhsingian, ‘I’ = Induan, ‘Ol’ = Olenekian, ‘An’ = Anisian, ‘Ldn’ – Ladinian; (D) ‘Rht’ = Rhaetian, ‘Htt’ = Hettangian, ‘Snm’ = Sinemurian, ‘Pl’ = Pliensbachian; (E) ‘Cm’ = Campanian, ‘Mst’ = Maastrichtian, ‘Dn’ = Danian, ‘St’ = Santonian, ‘Th’ = Thanetian.

We describe the effects of ecosystem engineers on within-formation diversity over each of the Big 5 mass extinction events in chronological order. In general, we find that effect sizes do not exhibit consistent responses through mass extinction events, nor when comparing between bioturbators and reef-builders (Fig. 3). There is also a broad pattern whereby effect sizes associated with both engineering groups decrease over Palaeozoic extinctions (although more severely in bioturbators), while Mesozoic extinction events overwhelmingly impact the effects of reef-builders.

Through the end-Ordovician mass extinction (Fig. 3A), positive effect sizes associated with both of reef-builders and bioturbators decrease over the extinction (Hirnantian-Rhuddanian) boundary, although they remain strongly positive in reef-builders. Effect sizes increase throughout the recovery period, such that both reef-builders and bioturbators have strong positive effect sizes by the Telychian. A similar pattern is evident over the late Devonian mass extinction (Fig. 3B), with effect sizes decreasing before and during the extinction event between the Givetian, Frasnian, and Famennian, before recovering to strong and significantly positive effects in the Carboniferous. The end-Permian mass extinction (Fig. 3C) stands out for having a strong impact on the effects of bioturbators, with strong effect sizes during the Wuchiapingian declining to only weakly positive effects during the Changhsingian and Induan, and to no effect during the Olenekian. In contrast, reef-builders have significantly positive effects during the Wuchiapingian and Changhsingian, and do not collapse to insignificance in the early Triassic recovery period.

The end-Triassic mass extinction (Fig. 3D) sees an increase in effect sizes for bioturbators, with weak effects during the Rhaetian increasing to significantly positive effects during the Hettangian and maintaining positive effects during the Sinemurian and Pliensbachian. Meanwhile, the effects of reef-builders are severely impacted, with significant effect sizes declining to no effect in the Hettangian and Sinemurian. Finally, bioturbators and reef-builders have opposite responses across the K-Pg mass extinction, with bioturbators increasing through the interval to strong positive effect sizes during the Danian and moderate positive effect sizes at the Selandian-Thanetian, while reef-builders decrease from strong positive effects during the Maastrichtian to insignificant effects by the Selandian-Thanetian (Fig. 3E).

### Evolutionary, diversity, and climate correlates

**Fig. 4:**
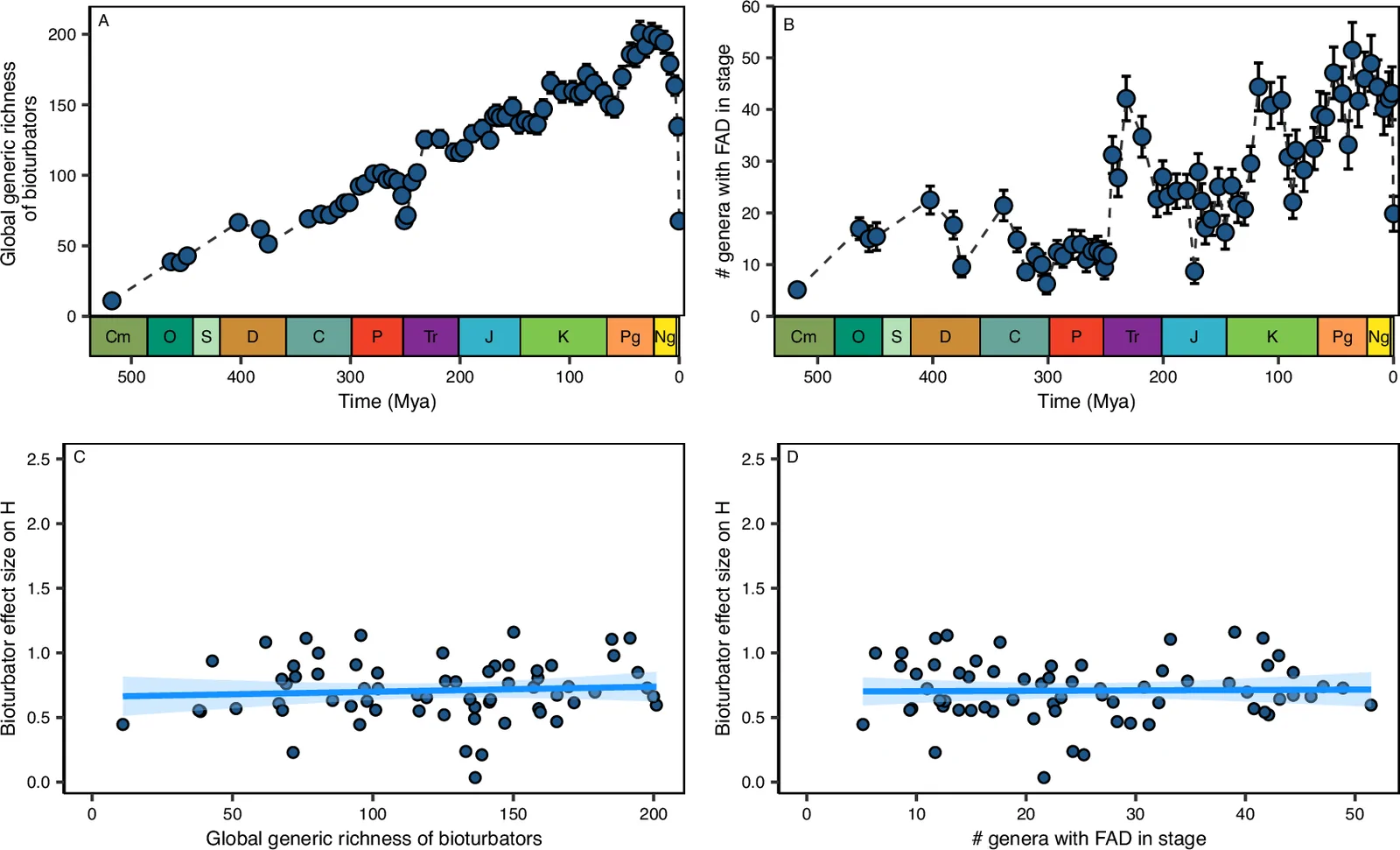
Evolution of bioturbator diversity as a predictor of effect size strength. **A** Global generic richness of bioturbators at each sage in the Phanerozoic. Points are mean stage-level richness of 1000 iterations, and error bars are ±1 standard deviation. **B** Number of bioturbating genera whose FAD (first appearance datum) are in each stage. Points are the stage-level mean number of new genera of 1000 iterations, and error bars are ±1 standard deviation. Dashed lines represent discontinuous curves, as some stages are excluded due to insufficient data for subsampling. **C** Effect of global generic richness of bioturbators on effect sizes, with linear regression fit through data (solid blue line) and 95% confidence intervals on the linear regression (light blue area). **D** Effect of the number of bioturbating genera whose FADs are in each stage, with linear regression fit through data (solid blue line) and 95% confidence intervals on the linear regression (light blue area). For A and B, geologic timescale abbreviations are the same as Fig. 1.

**Fig. 5:**
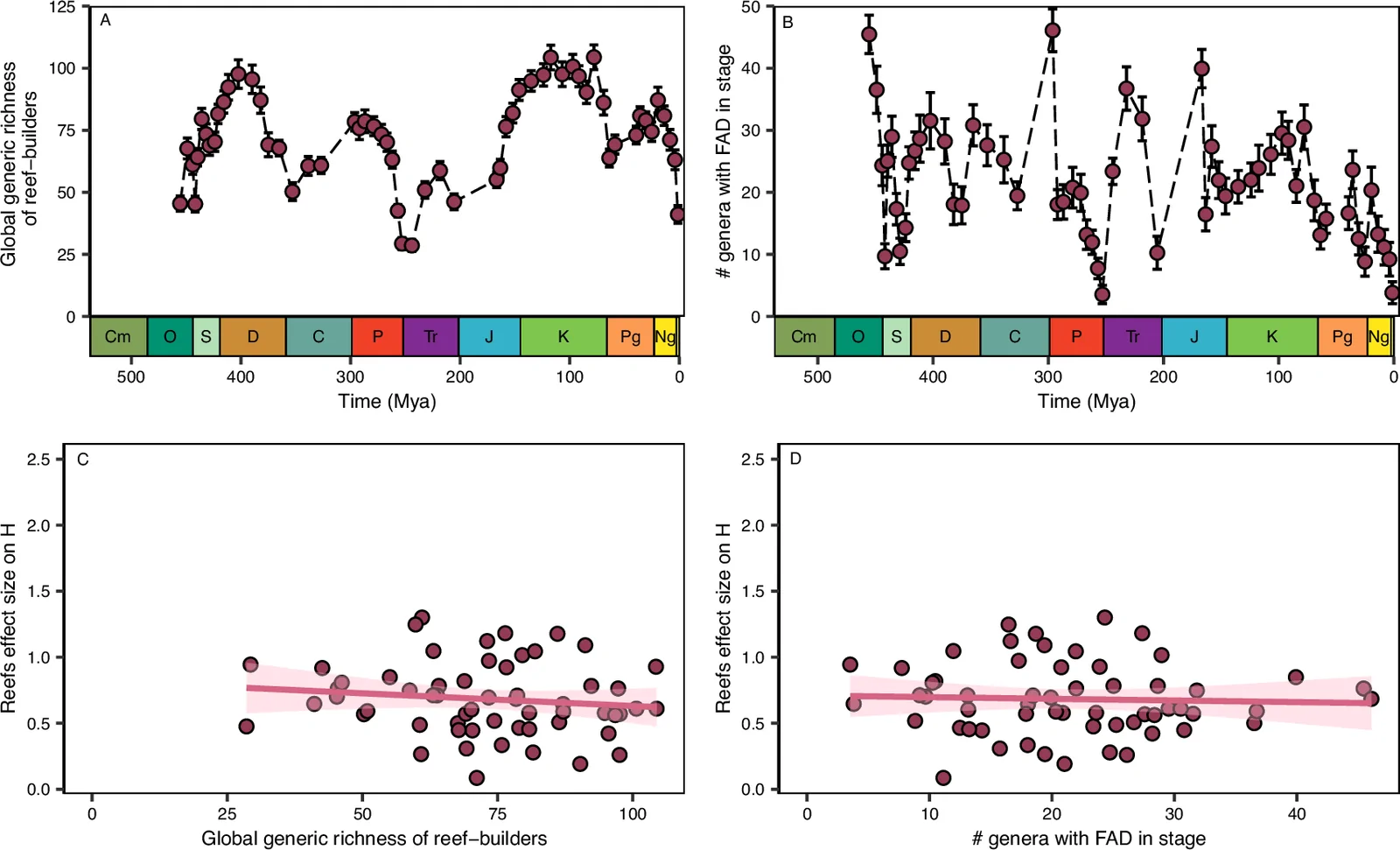
Evolution of reef-builder diversity as a predictor of effect size strength. **A** Global (stage-level, across all formations) generic richness of reef-building ecosystem engineers. Points are mean stage-level richness of 1000 iterations and error bars are ±1 standard deviation. **B** Number of reef-building genera whose FADs are in each stage. Points are the stage-level means of 1000 iterations and error bars are ±1 standard deviation. Dashed lines represent discontinuous curves. **C** Effect of global generic richness of reef-builders on effect sizes, with linear regression fit through data (solid pink line) and 95% confidence intervals on the linear regression (light pink area). **D** Effect of the number of reef-building genera whose FADs are in each stage, with linear regression fit through data (solid pink line) and 95% confidence intervals on the linear regression (light pink area). For **A** and **B**, geologic timescale abbreviations are the same as Fig. 1.

We find no clear relationship between the evolving diversity of ecosystem engineers and their effect sizes through the Phanerozoic. Bioturbators have a clear strong upwards trajectory in global richness (Fig. 4A) and, in general, there is an increasing trend in bioturbator generic originations among bioturbating taxa, with an increasingly greater number of new genera appearing in each stage through time (Fig. 4B). However, neither of these trends predict variability in effect sizes (Fig. 4C, D). Reef-builders do not exhibit similarly strong upward trajectories in global richness (Fig. 5A) or number of new genera arising per stage (Fig. 5B). As with bioturbators, neither can be used to predict effect size (Fig. 5C, D).

**Fig. 6:**
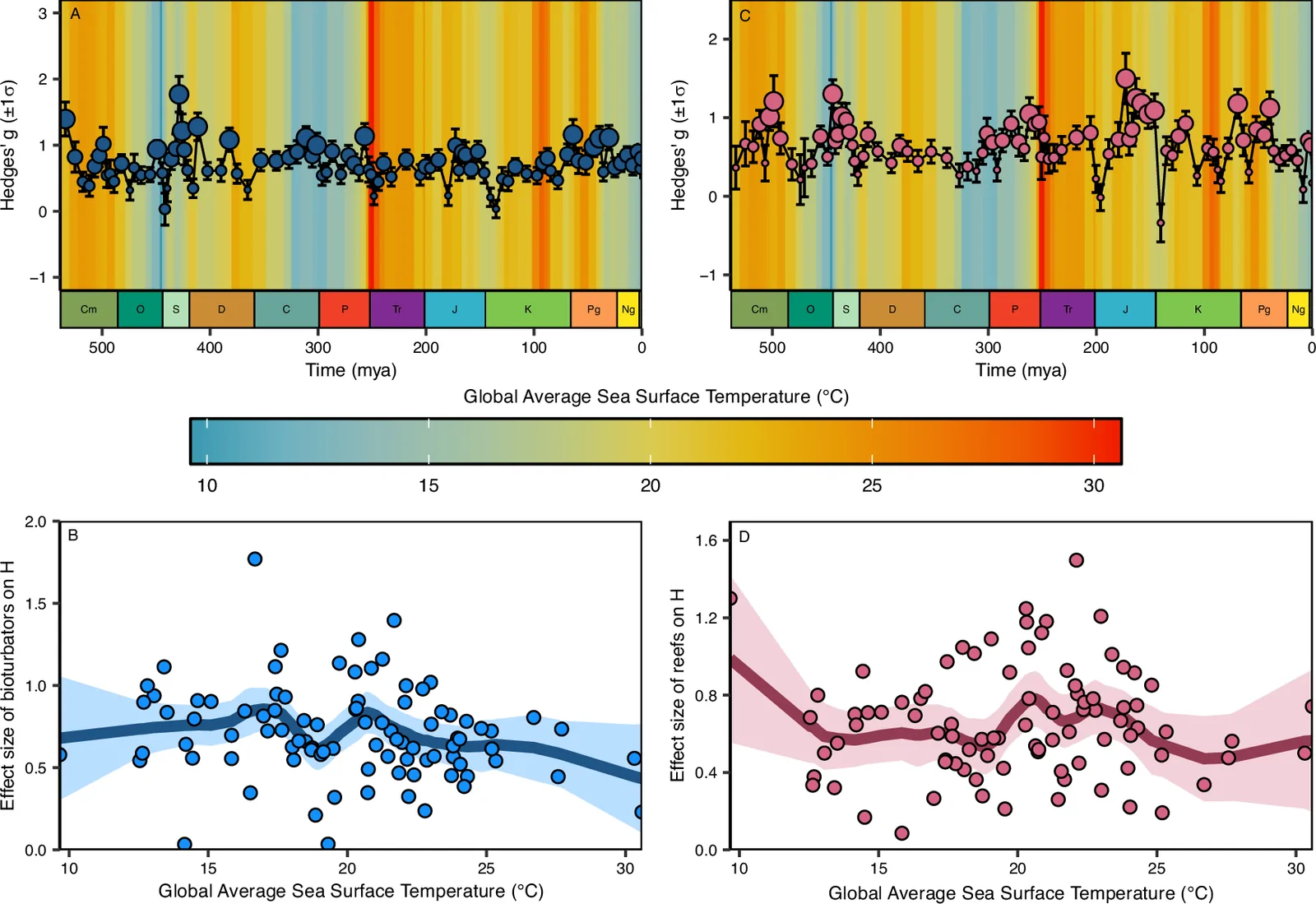
Climate as a predictor of ecosystem engineering effects of bioturbators (blue, left) and reef-builders (pink, right). **A** Hedges’ g effect sizes for bioturbators plotted on colour-coded temperatures bands for each stage. **B** Global average temperature relationship with effect sizes. **C** Hedges’ g effect sizes for reef-builders plotted on same colour-coded temperatures bands for each stage. **D** Global average temperature relationship with effect sizes. For **A** and **C**, climate band colours correspond to the global average temperature legend, where blue colours are colder and red colours are hotter. For **A** and **C**, effect sizes are the same as Figs. 1B, 2B; points are mean *g* for 1000 iterations with error bars as ±1 standard deviation. For **B** and **D**, curves are cross-validated loess curves with 95% confidence intervals. Global average temperatures are from ref. ^62^. Geologic timescale abbreviations on x axis of **A** and **B** follow those for Fig. 1.

Correlation tests between effect sizes and global average temperatures through time suggest that there may be a relationship between ecosystem engineering impact and an optimal temperature range in moderate climates (Fig. 6). Smaller effect sizes of bioturbators generally occur with climatic extremes, such as rapid global cooling associated with the end-Ordovician mass extinction and rapid global warming with the end-Permian mass extinction. However, we note that there are numerous intervals in which effect sizes collapse regardless of climate trends, while other intervals characterized by more moderate warming do appear to impact effect sizes (Fig. 6A). While we do not find a clear, robust relationship between temperature and effect sizes, the strongest effects of bioturbators do occur in moderate global temperatures in the range ~16–22 °C, and effect sizes decrease as the climate warms or cools towards extremes (Fig. 6B). Effect sizes of reef-builders, similar to bioturbators, collapse during intervals of severe and rapid global warming – such as during the end-Permian and end-Triassic mass extinctions – and rapidly changing effect sizes throughout the Mesozoic are associated with the warm Mesozoic hothouse interval (Fig. 6C). In contrast to bioturbators, however, we find that extreme global cooling may be less likely to drive collapses in the effect size of reef-builders, as strong effect sizes are also apparent during the Hirnantian cool interval (Fig. 6C). Additionally, there are numerous temperature states which are associated with insignificant effect sizes (Fig. 6D). While we do not find a clear relationship between climatic extremes and effect sizes of reef-builders, we do note that their strongest effect sizes – like bioturbators – are clustered around a moderate global climate between ~20–23 °C (Fig. 6D).

## Discussion

Our analyses illustrate that two important groups of ecosystem engineers – bioturbators and reef-building metazoans – have had persistent and positive impacts on biodiversity since at least the beginning of the Cambrian. In testing our initial hypotheses, we draw three main conclusions. First, the presence of both bioturbating and reef-building ecosystem engineers is significantly associated with increased biodiversity for the vast majority of the Phanerozoic. These results indicate a fundamental role of ecosystem engineers in driving community-scale ecological dynamics that are reflected in reconstructions of global Phanerozoic biodiversity. Second, we show that these two groups of ecosystem engineers have had variable effects in response to mass extinctions, with implications of differential response diversity in different ecosystem engineering evolutionary regimes. Third, while the evolution of diversity among ecosystem engineering groups is unlikely to have driven these trends, our results do suggest a possible role for climate in modulating the strength of the impact that ecosystem engineers have on biodiversity.

Positive effect sizes indicate that ecosystem engineering activities of bioturbators and reef-builders have fostered biodiversity over the Phanerozoic, just as ecosystem engineers do in modern marine communities^19^. These results reinforce the relationship between biodiversity and the distribution and availability of resources, which ecosystem engineers are known to directly influence^37^. Bioturbators increase resource heterogeneity by changing the three-dimensional physical characteristics and biogeochemistry of the seafloor. Bioturbators are also key for maintaining benthic-pelagic nutrient fluxes and thereby can also influence animals living in the water column^38^. Although bioturbation behaviours have certainly been evolving over the Phanerozoic^39^, complex ecosystem engineering activities which would have allowed bioturbators to significantly impact their environments have been present since the Ediacaran-Cambrian transition^40,41^. These established ecosystem engineering impacts can explain several intervals of very strong effect sizes. The strong Fortunian effect size likely reflects the Cambrian-Substrate Revolution, as bulldozing bioturbators’ activities opened niche space by increasing resource availability and the habitability of the seafloor^39,42^. As mixed-layer development increased through the Phanerozoic, nutrient cycling would have been particularly stimulated in bioturbated ecosystems^43^. Positive effect sizes in the Silurian, Devonian, and Carboniferous are likely associated with this mixed-layer development^43^, driven by the evolution of sediment-mixing deposit feeders during these intervals^44^. For example, the Homerian effect size peak is most likely driven by the invasion of deposit-feeding ecosystem engineers, which include rapidly burrowing deposit-feeding bivalves. Moreover, the Homerian sees an expansion in deposit feeders, being the stage with the highest proportion of deposit feeders out of all bioturbation feeding ecologies over the entire Phanerozoic^44^.

Reef-builders have similar ecosystem engineering roles, increasing resource heterogeneity through the construction of their three-dimensional biomineralized structures, impact on local-scale hydrodynamic patterns, creation of biosediments, and provision of new hard substrates^45,46^. These ecosystem engineering behaviours allow reef-builders to have positive impacts on biodiversity – including hosting biodiversity hotspots today and throughout the Phanerozoic^47^. We find this is not limited to coral ecosystem engineers – sponge reef-builders such as hexactinellids and stromatoporoids apparently had significant impacts on biodiversity early in the Phanerozoic. Our results illustrate positive effect sizes during intervals of the Cambrian when reefs are made entirely of hexactinellid sponges, and also during the Hirnantian, when stromatoporoids and corals make up roughly equal proportion of reef-building ecosystem engineers^44^, driving the positive effect size peak (Fig. 2). Both bioturbators and reef-builders were thus ecologically important ecosystem engineers early in the Phanerozoic, perhaps earlier than expected, given that modern-type bioturbation (e.g., large three-dimension network burrows and extensive deep sedimentary mixed layers) and reef-builders (e.g., aragonitic, branching- and framework-building, zooxanthellate scleractinian corals) did not arise until the Mesozoic. This indicates that engineers that may be hypothesised to have weaker impacts – such as smaller, shallower invertebrate burrowers and reef mound- and biostrome-constructors – still played critical ecological roles in the Palaeozoic, and their impacts should be considered in the context of their own contemporaneous ecosystems and Earth systems states. Interestingly, the Devonian reef peak – in which reefs are at their most dominant in terms of number and abundance of reef ecosystems^44^ – is not associated with strong positive effect sizes. Effect sizes during this interval are driven by in-phase declines in biodiversity of both reef and non-reef associated taxa (Fig. 2A), indicating environmental prevalence and geographic distribution are not always coupled with ecosystem engineering impact. There is a clear shift in the Mesozoic and Cenozoic, when scleractinian corals evolve and the amplitude of effect size change increases (Fig. 2). When scleractinian coral reef ecosystems collapse, effect sizes suffer even when other reef-builders persist. For example, much of the weak and insignificant effect sizes during the Cretaceous are associated with the rise of rudist bivalves^44^, which were apparently less efficient ecosystem engineers in terms of driving increased biodiversity, possibly because they did not build ‘true reef’ structures with similar levels of complexity relative to same-age corals and sponges (Fig. 2).

Mass extinctions do not impact bioturbators and reef-builders equally, nor do bioturbators and reef-builders respond to every mass extinction and interval of climate-driven stress in the same way. This result underscores the diversity of stress responses that encompasses ecosystem engineers as a large functional group – not all ecosystem engineering taxa should be expected to have the same ameliorating effects on environmental stress. Bioturbators are particularly sensitive to mass extinctions and climate-driven stress, exhibiting collapses in effect size around the end-Ordovician, end-Devonian, and end-Permian mass extinctions (Fig. 3), as well as the Toarcian Ocean Anoxic Event (T-OAE) (Fig. 1). These results contrast previous findings from the trace fossil record, indicating that the strength and complexity of bioturbation ecosystem engineer behaviours persist across even the worst mass extinction events^34,35^. Effect sizes still collapse despite the resiliency of these ecosystem engineers, likely because the high-impact complex behaviours remain rare, and environmental changes (e.g., eutrophication^48^, euxinia^49^) hindered even surviving strong bioturbators’ abilities to mitigate environmental stress. However, bioturbator effects are largely unaffected by the end-Triassic and Cretaceous-Paleogene mass extinctions, suggesting modern-type bioturbators that evolved during the Mesozoic Marine Revolution may have been more efficient at ameliorating climate-driven environmental stress, as hypothesised in modern ecosystems^22^.

The effects of reef-builders are more persistent through Palaeozoic mass extinctions, likely due to the diversity of reef-builders. For example, while stromatoporoids were a major component of early Palaeozoic reefs, their extinction at the end of the Devonian did not result in a collapse in reef-builder effects because rugose and tabulate corals occupied the same ecosystem engineering roles, and often within the same communities^44^. The persistence of reef-builder effects through the end-Permian mass extinction is particularly surprising given its associated coral reef collapse^50^. Reef-associated global diversity certainly significantly declined during this interval^50^. However, our formation-level results indicate this would have been primarily due to the loss of the number of reef ecosystems (Supplementary Table 3), rather than the inhibition of surviving reef-builders’ ecosystem engineering strength, as other non-coral reef-builder survivors in the early Triassic^44,50^ maintained communities where biodiversity was still, on average, higher than in non-reef communities (Fig. 2A). Notably, there is an apparent shift with the evolution of scleractinian corals, when effect sizes tend to crash most severely when they became Earth’s dominant reef-builders. This emphasizes the long-existing sensitivity of our modern corals^51^, which appear to be the only ecosystem engineering group that consistently loses strength of impact in times of environmental stress in hothouse climates (Fig. 6), reflecting modern observations that global warming is severely inhibiting the ecosystem engineering strength of reef-building corals^45^.

The evolution of taxonomic diversity of ecosystem engineers did not result in stronger influences on biodiversity (Fig. 4), in contrast to previous hypotheses^24^. This most likely indicates a decoupling between taxonomic and functional diversity of ecosystem engineers. If taxonomic radiations do not have parallel functional radiations that give rise to novel ecosystem engineering behaviours and innovations, ecosystem engineers will continue to impact resource availability and distribution in essentially the same ways, thus having limited impacts on community-level diversity. Decoupling of taxonomic and functional diversity appears to be common in the fossil record^52,53^, so this is perhaps unsurprising, but future work is needed to identify ecosystem engineer functional groups from the fossil record and then investigate their Phanerozoic functional diversity.

It is evident that ecosystem engineers and their effects are impacted by the environment. We do find support for a relationship between climate and ecosystem engineering strength, following modern observations that climatic extremes inhibit animal behaviours with crucial ecosystem engineering impacts^45^. Strong ecosystem engineering effect sizes tend to cluster in moderate global temperatures, suggesting a climatic thermal optimum that permits both bioturbators and reef-builders to most significantly promote biodiversity. Previously, it has been demonstrated that fossil biodiversity can be explained by global temperature trends, where peaks in biodiversity are strongly associated with moderate temperatures relative to cold and hot extremes^54^. In these optimal temperature ranges, biodiversity already has the potential to be high, and ecosystem engineers are able to boost biodiversity by impacting resource availability and distribution. Whereas, in climatic extremes, when the communities and ecosystem engineers themselves face physiological stress, the modification of resource flows has less impact on biodiversity. However, there are instances of weak and insignificant effect sizes in the same optimal temperature ranges (Fig. 6), indicating that thermal stress is not the sole driver of ecosystem engineering strength. The within-palaeoenvironment analyses also indicate some differences in effect sizes between lithologies and bathymetries (Supplementary Figs. 3, 4). While the trend of significantly positive effect sizes persists in constrained palaeoenvironments, we do find occasional significant differences in specific time periods. For example, only comparing carbonate environments to calculate effect sizes of reef-builders results in occasionally more dynamic effect size changes (e.g., at the Jurassic-Cretaceous boundary), and effect sizes in deep marine settings of bioturbators occasionally decline to significantly negative effects (such that biodiversity is higher where bioturbators are absent). These specific differences that arise in unique palaeoenvironments warrant further investigation, but ultimately, they suggest that an ecosystem engineering group’s effect is not always equal across all environments throughout Earth's history. Overall, ecosystem engineers’ abilities to increase biodiversity more likely depend on a holistic suite of extrinsic factors that control the background level of resources that they influence.

Both bioturbating and reef-building ecosystem engineers play an important role in global biodiversity curves by influencing ecological dynamics within their communities. These ecosystem engineers can facilitate a global rise in biodiversity when their diversity effect sizes are significantly positive, and they are present in a large number of communities. By combining the results of this study with recent advances in our understanding of the abundance of ecosystem engineer-influenced communities^44^, inferences can be made about how and when ecosystem engineers have become important drivers of the global-scale Phanerozoic biodiversity curve. The impact on global biodiversity is most clear with bioturbators, which became increasingly prevalent in more formations beginning in the Carboniferous^44^, while at the same time having persistent effects on biodiversity (Fig. 1). In the Cenozoic, particularly, biodiversity in formations with bioturbators remained relatively stable, while biodiversity in formations without bioturbators decreased. These results may, therefore, support a bioturbating ecosystem engineer-driven mechanism for the rise in biodiversity throughout the Cenozoic^2^, particularly if future work finds elevated origination rates strongly associated with bioturbating ecosystem engineers. Somewhat counterintuitively, reef-builders may not have this same global effect, despite hosting biodiversity hotspots. Because reef-builders do not have increasingly stronger effect sizes through time (Fig. 2B), and because reef ecosystems have also not become increasingly common through the Phanerozoic^44^, the ecosystem engineering effects of reef-builders are unlikely to be drivers of a long-term upwards trend in global biodiversity. However, we emphasize that this does not negate the ecological importance of reefs – that reef-builders have a persistently strong effect size on biodiversity over the entire Phanerozoic still underscores their long-term importance for influencing local ecology and hosting biodiversity hotspots.

Finally, our results also reveal numerous times throughout Earth history when changes in ecosystem engineer-associated biodiversity are out of phase with the rest of the marine biosphere (Figs. 1B, 2B), emphasizing the role that ecosystem engineers play in shaping the spatial structure of Phanerozoic biodiversity^6^. Consider the Jurassic-Cretaceous boundary, for example, when diversity plummets in formations with ecosystem engineers and increases in formations without ecosystem engineers. The biodiversity collapse in communities with bioturbators and reef-builders is reflected in a global decline in biodiversity^2^, but this “global” decline would have been limited to only specific regions, apparently unbalanced by the increase in diversity elsewhere. Overall, while environmental (e.g., climate- and tectonic-driven) variability certainly impacts regional diversity that sums to global curves^6^, our findings reveal that ecosystem engineers also play a critical role in driving community-level ecological dynamics that are reflected at the global scale. This ultimately underscores the importance of biotic interactions and community assembly for understanding the evolution of the Phanerozoic biosphere.

## Methods

### Data cleaning and processing

Marine Phanerozoic fossil occurrence data were downloaded from the Paleobiology Database (accessed 1 November 2023). The initial raw dataset was cleaned and stratigraphically binned following ref. ^55^ to remove problematic taxa, ichnotaxa, and other fossils with uncertain affinities, as well as fossil occurrences which could not be unambiguously assigned to a Phanerozoic stage. The dataset was then supplemented with known missing data for reef-builders in Cambrian Stage 10^56^. The dataset was also cleaned at the formation level to ensure that fossil occurrences were correctly grouped with their co-occurrences in their geologic formations. The list of formations in the dataset was first manually inspected to remove uncertain formations, such as those beginning with “?” or those entered as “unnamed.” Lithological descriptions as formation names were also removed, such as formations simply named “lower calcareous grit” which spanned multiple time intervals and a large spatial area. Formation synonyms were combined into a single formation name; for example, “Much Wenlock,” “Much Wenlock Limestone,” and “Wenlock Limestone” were all grouped into a single formation. Similarly, formations with spelling errors, differences in character cases, and different languages (e.g., “Nusplingen Limestone” and “Nusplingen Plattenkalk”) were appropriately combined into the appropriate single formations. We cross-referenced all formation re-naming and grouping with fossil occurrence coordinates and recorded ages to ensure that taxa were being correctly grouped together in space and time. We did not combine formations that were split into higher resolution units (e.g., fossils assigned to the “Lower Bringewood” were not combined with the “Bringewood”) to maintain the smallest possible units of co-existing taxa. Finally, we removed any fossil occurrences that were never initially assigned to a geological formation. After processing the data at the taxonomic, temporal, and formation levels, the final dataset contains 565,064 fossil occurrences of 27,507 genera across the entire Phanerozoic.

### Dataset assembly

The full dataset was used to identify bioturbators and reef-building genera following ref. ^44^. For bioturbators, we used the life habit, motility, and diet information assigned to fossil occurrences in the PBDB. We broadly use bioturbation here to include both biomixing – the mixing of solid-state sediment particles – and bioirrigation – the mixing of sediment solutes with the overlying water column^57^. First, we identified all infaunal genera by subsetting fossil occurrences that are described as occupying an infaunal tier in their life habit information. By identifying all infaunal genera, we collect any infaunal motile biomixer, such as shallow tier deposit feeders, as well as the non-motile infaunal bioirrigators, such as the stationary deep tier suspension feeders. We also identified grazers and sediment bulldozers by subsetting epifaunal, motile, deposit feeders/grazers from the life habit, motility, and diet information, respectively. We then manually screened this list of genera to check for and remove taxa that are not bioturbators but had been incorrectly or ambiguously ecologically described. In total, we identified 4,247 bioturbator genera, approximately 70% of which are infauna, with the remaining genera representing epifaunal grazers and sediment bulldozers. We focused on ten key reef-building metazoan groups from the Kiessling and Flugel (2002)^58^ that are represented in the PBDB with sufficient metadata and lack ecological and taxonomic uncertainty^44^: archaeocyathids, hexactinellids, stromatoporoids, reef-building hydrozoans, tube worms (serpulids and sabellarids), chaetetids, rudist bivalves, tabulate corals, rugose corals, and scleractinian corals. Furthermore, we note that we may lack some reef-building ecosystem engineers in this dataset, including non-metazoan reef-builders like stromatolites and calcareous algae, and future work could include and compare the effects of these other reef-builders. Finally, we note that this list includes a variety of reef functional types, from constructors of true reefs to biostromes to reef mounds^58^. Despite this functional diversity, all groups should have reef ecosystem engineering impacts at some level, from hydrodynamic effects to water column filtering. In total, we identified 1983 reef-building genera, the majority of which are either coral reef-builders (1482 genera) or sponge reef-builders (359 genera). Finally, we note that for both bioturbators and reef-builders, these identifications are based on the hypothesized propensity for the animal to do their ecosystem engineering behaviours. Direct evidence of ecosystem engineering processes is absent from the body fossil record, so these identifications are only an inference of ecosystem engineering. Here, we assume that if an animal can possibly do their typical ecosystem engineering behaviours (e.g., corals constructing true reefs, or infaunal bioturbators significantly mixing sediments), then those associated ecosystem engineering processes did occur.

### Subsampling protocol

Due to taphonomic filters and uneven sampling efforts, diversity trends constructed from the fossil record are subject to temporal and spatial biases, which we minimize with stage-level and formation-level subsampling. Temporal biases arise where there are large variations in the number of fossil occurrences preserved in each stage. To account for this bias, we standardize the number of fossil occurrences in our analyses through time by randomly subsampling 700 fossil occurrences (set below the smallest stage-level population size of fossil occurrences) in each stage through the Phanerozoic. A spatial bias also arises from uneven sampling efforts across the same-stage formations, such that very well-studied formations (i.e., those with a large number of collections in the PBDB) typically contain a larger number of fossils, which generally will have higher diversity compared to less well-studied formations (i.e., those with only a small number of collections in the PBDB). Because our analyses compare ecological metrics between formations, this is an important bias to minimise. We tested three different within-formation subsampling protocols: one treatment where 20 fossil occurrences are subsampled per formation, a second treatment where 5 PBDB collections are subsampled per formation, and a third where we do not apply any within-formation subsampling. For each of these treatments, we compared the relationship between our effect size results and the difference in the average number of collections in formations with ecosystem engineers and collections in formations without ecosystem engineers – in other words, the difference in sampling effort. To identify the within-formation subsampling protocol that best minimises the bias, we selected the best relationship between effect size and differences in sampling effort and then the protocol that returned the lowest *R*^2^ value, or the worst statistical fit. Ultimately, we determined that subsampling 20 occurrences per formation best minimises the spatial sampling bias (Supplementary Figs. 6, 7). We also note that the spatial sampling bias is much weaker for reef-builders than for bioturbators (Supplementary Figs. 6, 7). To further address the impact of spatial biases, we conducted a second set of analyses in which we divided our data into equal-area grid cells, subsampled 700 random fossil occurrences per stage, and then 10 random fossil occurrences per grid cell (Supplementary Figs. 8, 9). We find negligible differences between this protocol and the formation-level subsampling (Supplementary Fig. 10), likely because most of the fossil occurrences from the same formations are not geographically widespread. To test the impacts of under-sampled regions, we also conducted a set of effect size analyses on a dataset in which data from formations and equal area grid cells that do not contain 20 occurrences in each stage were removed from the dataset prior to analysis. Although we find some stage-by-stage differences in some threshold tests, this protocol has minimal statistical impact on broad Phanerozoic results (Supplementary Fig. 11). Ultimately, our final subsampling protocol takes 700 random fossil occurrences per stage, and from the formations represented in those subsampled occurrences, we take a further random 20 occurrences per formation. Finally, subsampling was repeated for 1000 iterations per stage. Results presented here are the means of those 1000 iterations, with uncertainty bars representing the mean ±1 standard deviation. Note that these subsampling procedures differ slightly for calculations of ecosystem engineer diversity and generic originations (see *Climatic and evolutionary correlates*).

### Effect size analyses

A modern ecological analysis assessing the effects of ecosystem engineers on biodiversity would make comparisons between localities where ecosystem engineers are present and localities where ecosystem engineers are absent^19,29,59^. The analogue for this in palaeoecology is to compare ecological metrics across same-age geological formations, which are the best representation of animals living in the same place at the same time. We note that formations are not a perfect “snapshot” of ecology unless they are *Lagerstätten*, so as with all analyses of community palaeoecology, there are some biases that will arise due to transport, death assemblages, and time averaging, which cannot be avoided due to the nature of the fossil record. We make our comparisons between formations of the same stratigraphic stages, which do span millions of years, but are the smallest temporal unit of the geologic timescale that we can conduct these types of analyses on using the PBDB. In our main analyses, we compare across different lithologies and bathymetries, as ecosystem engineers occupy a variety of palaeoenvironments in each stage (Supplementary Figs. 2,4). However, to determine that effect sizes are not driven by palaeoenvironmental difference, we also conducted effect size analyses constrained within siliciclastic, carbonate, shallow, and deep environments (Supplementary Figs. 3,5).

For each stage, data is divided into two pools: fossil occurrences in formations where either bioturbators or reef-builders are present, and fossil occurrences in formations where they are absent. We do note that in these absence pools, the other type of ecosystem engineer (and non-bioturbator or non-reef building ecosystem engineers) is present, so that this approach tests directly for the effects of a single ecosystem engineering group rather than ecosystem engineering as a whole. Within each formation in each stage, we calculate Shannon’s Diversity (*H*), which takes into account both richness and evenness^25^,

1 $$H={\sum}_{i}^{S}{p}_{i}\,{ln}\,{p}_{i}$$

where, in our analyses, *p*_*i*_ is the proportion of fossil occurrences present belonging to genus *i*, *S* is the total number of genera in the formation, and *H* is the Shannon’s Diversity index value. We include the ecosystem engineering genera themselves in these analyses because we assume that ecosystem engineers are subject to any ecological change caused by other ecosystem engineers in their communities. However, we also established that the removal of ecosystem engineers has minimal impact on calculated effect sizes (Supplementary Fig. 1) due to ecosystem engineers being less abundant than non-ecosystem engineering genera.

Finally, we compared *H* between formations with and without ecosystem engineers using the Hedges’ g statistic for effect size (*g*), which compares the standardised mean difference between two distributions of data:

2 $$g=\,\frac{{\bar{x}}_{1}-\,{\bar{x}}_{2}\,}{{s}^{*}}.$$

Here, ${\bar{x}}_{1}$ is the average *H* across the formations with ecosystem engineers, ${\bar{x}}_{2}$ is the average *H* across the formations without ecosystem engineers, and s^*^ is the pooled standard deviation^26^. The pooled standard deviation is defined as

3 $${s}^{*}=\,\sqrt{\frac{\left({n}_{1}-1\right){s}_{1}^{2}+\left({n}_{2}-1\right){s}_{2}^{2}}{{n}_{1}+\,{n}_{2}-2}},$$

where *n*_*1*_ is the number of formations containing ecosystem engineers, *n*_*2*_ is the number of formations without ecosystem engineers, *s*_*1*_ is the standard deviation of *H* across the formations with ecosystem engineers, and *s*_*2*_ is the standard deviation of *H* across formations without ecosystem engineers^26^. We calculate *g* for each stage, so the two distributions of *H* measurements are comprised of formations that are all stratigraphically the same age. When Hedges’ *g* is positive, it reflects diversity being higher where ecosystem engineers are present, and when Hedges’ *g* is negative, it reflects diversity being higher where ecosystem engineers are not present. Finally, Hedges’ *g* has rule-of-thumb thresholds for effect size^60^: weak effects are considered where 0.5 > |*g*| ≥ 0.2, moderate effects are considered where 0.8 > |*g*| ≥ 0.5, and strong effects are considered where effects are |*g*| ≥ 0.8. However, these thresholds are context-dependent, so it is important to consider the effect sizes in the context of other ecological studies of ecosystem engineers^61^. Other meta-analyses of ecosystem engineers use the same thresholds and may consider significance where |*g*| ≥ 0.2^29,59^, but to be conservative for the fossil record, we only consider effect sizes to be significant where they meet strong and moderate thresholds (|*g*| ≥ 0.5) and uncertainty bounds do not cross the weak-moderate threshold.

### Climatic and evolutionary correlates

We compare measured effect sizes with global temperatures and ecosystem engineer richness and origination to investigate whether effect sizes can be predicted by the evolutionary trajectories of ecosystem engineers and the Earth’s climate. Global ecosystem engineer richness was calculated as the number of unique bioturbating or reef-building genera present in each stage of the Phanerozoic. We also calculated the number of ecosystem engineering genera whose first appearance datum (FAD) is in each stage. In producing these two curves, we apply the same bootstrap subsampling procedure to our bioturbator and reef-builder effect sizes, although we normalize each stage to 150 fossil occurrences. To meet this subsampling quota, we exclude stages with insufficient data (Supplementary Table 5). We compared both richness and FAD calculations to effect sizes in each stage to assess whether the evolution of ecosystem engineer diversity is a reliable predictor of effect size variations throughout the Phanerozoic. We exclude *g* from the stages with insufficient data to meet the 150 fossil occurrences subsampling quota. We test the hypothesis that as ecosystem engineers taxonomically diversify, effect sizes will increase, and thus we calculated linear regressions to test the strength of these two correlates. We also compared effect sizes to global average temperatures from ref. ^62^. We took the million-year global average temperatures from this initial dataset and computed a mean global average temperature per stage. These stage-level global average temperatures were then compared to the effect sizes. We test the hypothesis that effect sizes should increase in some temperature optimum in moderate climates, following the metabolic theory of ecology^63^ and established relationships between fossil biodiversity and moderate climates^54^. We computed cross-validated LOESS curves for the relationship between climate and effect sizes of bioturbators and reef-builders.

#### Software and packages

We performed all data manipulation, cleaning and analyses using version 4.2.2. of the programming language R^64^. To process, clean, and stratigraphically bin PBDB data, we use the package *divDyn*^55^. We use the *tidyverse* packages for all data manipulation^65^. To conduct spatial subsampling, we use the R packages *palaeoverse*^66^ and *terra*^67^. To compute cross-validated LOESS curves, we use *fANCOVA*^68^. For data visualisation, we use the packages *ggplot2*^69^, *deeptime*^70^, and *wesanderson*^71^.

### Reporting summary

Further information on research design is available in the Nature Portfolio Reporting Summary linked to this article.

## Supplementary information


Supplementary Information
Reporting Summary
Transparent Peer Review file


## Data Availability

Phanerozoic fossil data were originally downloaded from the Paleobiology Database (paleobiodb.org) on 1 November 2023 using the following terms: time intervals = Cambrian through Holocene; environment = ‘any marine’; additional output options = ‘all’. After cleaning and processing this data, the full Phanerozoic datasets needed to reproduce these analyses are freely available in the Data folders in the associated GitHub repository (https://github.com/atcribb/Ecosystem-Engineers-Effects) and Zenodo repository (https://doi.org/10.5281/zenodo.19824423). All diversity and effect size results datasets generated in this study have been deposited in these repositories and can be obtained by downloading the relevant files in the Output folders (as outlined by the repository README). Downloading the repository and running each script according to the instructions given in the README will also generate each results dataset.

## Code availability

R scripts used in this study are available in the GitHub Repository https://github.com/atcribb/Ecosystem-Engineers-Effects and on Zenodo https://doi.org/10.5281/zenodo.19824423. These R scripts will reproduce each analysis and all figures in the main text and supplementary materials.

## References

[1] Sepkoski, J. J. A factor analytic description of the Phanerozoic marine fossil record. *Paleobiology* **7**, 36–53 (1981).

[2] Alroy, J. et al. Phanerozoic trends in the global diversity of marine invertebrates. *Science* **321**, 97–100 (2008). 18599780 10.1126/science.1156963

[3] Bush, A. M. & Payne, J. L. Biotic and abiotic controls on the Phanerozoic history of marine animal biodiversity. *Annu. Rev. Ecol. Evol. Syst.* **52**, 269–289 (2021).

[4] Alroy, J. The shifting balance of diversity among major marine animal groups. *Science* **329**, 1191–1194 (2010). 20813951 10.1126/science.1189910

[5] Sepkoski, J. J., Bambach, R. K., Raup, D. M. & Valentine, J. W. Phanerozoic marine diversity and the fossil record. *Nature* **293**, 435–437 (1981).

[6] Close, R. A., Benson, R. B. J., Saupe, E. E., Clapham, M. E. & Butler, R. J. The spatial structure of Phanerozoic marine animal diversity. *Science* **368**, 420–424 (2020). 32327597 10.1126/science.aay8309

[7] Sadler, P. M. Sediment accumulation rates and the completeness of stratigraphic sections. *J. Geol.* **89**, 569–584 (1981).

[8] Jablonski, D., Roy, K., Valentine, J. W., Price, R. M. & Anderson, P. S. The impact of the pull of the recent on the history of marine diversity. *Science* **300**, 1133–1135 (2003). 12750517 10.1126/science.1083246

[9] Womack, T. M., Crampton, J. S. & Hannah, M. J. The Pull of the Recent revisited: Negligible species-level effect in a regional marine fossil record. *Paleobiology* **46**, 470–477 (2020).

[10] Mayhew, P. J., Bell, M. A., Benton, T. G. & McGowan, A. J. Biodiversity tracks temperature over time. *Proc. Natl. Acad. Sci.* **109**, 15141–15145 (2012). 22949697 10.1073/pnas.1200844109 PMC3458383

[11] Sperling, E. A. et al. Breathless through time: Oxygen and animals across Earth’s history. *Biol. Bull.* **243**, 184–206 (2022). 36548971 10.1086/721754

[12] Zaffos, A., Finnegan, S. & Peters, S. E. Plate tectonic regulation of global marine animal diversity. *Proc. Natl. Acad. Sci.* **114**, 5653–5658 (2017). 28507147 10.1073/pnas.1702297114 PMC5465924

[13] Peters, S. E. Geologic constraints on the macroevolutionary history of marine animals. *Proc. Natl. Acad. Sci.* **102**, 12326–12331 (2005). 16105949 10.1073/pnas.0502616102 PMC1194909

[14] Ezard, T. H. G., Aze, T., Pearson, P. N. & Purvis, A. Interplay between changing climate and species’ ecology drives macroevolutionary dynamics. *Science* **332**, 349–351 (2011). 21493859 10.1126/science.1203060

[15] Song, H. et al. Thresholds of temperature change for mass extinctions. *Nat. Commun.* **12**, 4694 (2021). 34349121 10.1038/s41467-021-25019-2 PMC8338942

[16] Marshall, C. R. Explaining the Cambrian “Explosion” of animals. *Annu. Rev. Earth Planet. Sci.* **34**, 355–384 (2006).

[17] Vermeij, G.J. Evolution and Escalation: An Ecological History of Life (Princeton University Press). (1987).

[18] Jones, C. G., Lawton, J. H. & Shachak, M. Organisms as ecosystem engineers. *Oikos* **69**, 373 (1994).

[19] Romero, G. Q., Gonçalves-Souza, T., Vieira, C. & Koricheva, J. Ecosystem engineering effects on species diversity across ecosystems: a meta-analysis. *Biol. Rev.* **90**, 877–890 (2015). 25174581 10.1111/brv.12138

[20] Benson, R. B. J., Butler, R., Close, R. A., Saupe, E. & Rabosky, D. L. Biodiversity across space and time in the fossil record. *Curr. Biol.* **31**, R1225–R1236 (2021). 34637736 10.1016/j.cub.2021.07.071

[21] Hastings, A. et al. Ecosystem engineering in space and time. *Ecology Letters.* **10**, 10.1111/j.1461-0248.2006.00997.x (2007). 17257103

[22] Byers, J. E. Using ecosystem engineers to enhance multiple ecosystem processes. *Funct. Ecol.* **38**, 22–36 (2024).

[23] Byers, J. E. et al. Using ecosystem engineers to restore ecological systems. *Trends Ecol. Evol.* **21**, 493–500 (2006). 16806576 10.1016/j.tree.2006.06.002

[24] Erwin, D. H. Macroevolution of ecosystem engineering, niche construction and diversity. *Trends Ecol. Evol.* **23**, 304–310 (2008). 18457902 10.1016/j.tree.2008.01.013

[25] Shannon, C. E. A mathematical theory of communication. *Bell Syst. Tech. J.* **27**, 379–423 (1948).

[26] Hedges, L. V. Distribution theory for Glass’s estimator of effect size and related estimators. *J. Educ. Stat.* **6**, 107–128 (1981).

[27] Gutiérrez, J. L. & Jones, C. G. Physical ecosystem engineers as agents of biogeochemical heterogeneity. *Bioscience* **56**, 227–236 (2006).

[28] Romić, I. & Nakajima, Y. Ecosystem engineering as an energy transfer process: a simple agent-based model. *Theor. Ecol.* **11**, 175–187 (2018).

[29] Trepel, J. et al. Meta-analysis shows that wild large herbivores shape ecosystem properties and promote spatial heterogeneity. *Nat. Ecol. Evol.* **8**, 705–716 (2024). 38337048 10.1038/s41559-024-02327-6

[30] Gearty, W., Uricchio, L. H. & Lyons, S. K. Investigating the biotic and biotic drivers of body size disparity in communities of non-volant terrestrial mammals. *Glob. Ecol. Biogeogr.* 10.1111/geb.13913 (2024).

[31] Raup, D. M. & Sepkoski, J. J. Mass extinctions in the fossil record. *Science* **215**, 1501–1503 (1982). 17788674 10.1126/science.215.4539.1501

[32] Hofmann, R., Buatois, L. A., MacNaughton, R. B. & Mángano, M. G. Loss of the sedimentary mixed layer as a result of the end-Permian extinction. *Palaeogeogr. Palaeoclimatol. Palaeoecol.* **428**, 1–11 (2015).

[33] Kiessling, W. Geologic and biologic controls on the evolution of reefs. *Annu. Rev. Ecol. Evol. Syst.* **40**, 173–192 (2009).

[34] Cribb, A. T. & Bottjer, D. J. Complex marine bioturbation ecosystem engineering behaviors persisted in the wake of the end-Permian mass extinction. *Sci. Rep.* **10**, 1–8 (2020). 31937801 10.1038/s41598-019-56740-0 PMC6959249

[35] Feng, X. et al. Resilience of infaunal ecosystems during the Early Triassic greenhouse. *Earth. Sci. Adv.* **8**, 10.1126/sciadv.abo0597 (2022). PMC9242451 35767613

[36] Dimitrijević, D., Santodomingo, N. & Kiessling, W. Reef refugia in the aftermath of past episodes of global warming. *Coral Reefs* **43**, 1431–1442 (2024).

[37] Sanders, D. & Frago, E. Ecosystem engineers shape ecological network structure and stability: A framework and literature review. *Funct. Ecol.* 10.1111/1365-2435.14608 (2024).

[38] Lohrer, A. M., Thrush, S. F. & Gibbs, M. M. Bioturbators enhance ecosystem function through complex biogeochemical interactions. *Nature* **431**, 1092–1095 (2004). 15470385 10.1038/nature03042

[39] Buatois, L. A. et al. Quantifying ecospace utilization and ecosystem engineering during the early Phanerozoic—The role of bioturbation and bioerosion. *Sci. Adv.* **6**, eabb0618 (2020). 32851171 10.1126/sciadv.abb0618 PMC7428343

[40] Cribb, A. T. et al. Increase in metazoan ecosystem engineering prior to the Ediacaran–Cambrian boundary in the Nama Group, Namibia. *R. Soc. Open Sci.* **6**, 190548 (2019). 31598294 10.1098/rsos.190548 PMC6774933

[41] Darroch, S. A. F. et al. The trace fossil record of the Nama Group, Namibia: Exploring the terminal Ediacaran roots of the Cambrian explosion. *Earth-Sci. Rev.* **212**, 103435 (2021).

[42] Bottjer, D. J., Hagadorn, J. W. & Dornbos, S. Q. The Cambrian Substrate Revolution. *GSA Today* **10**, 1–7 (2000).

[43] Tarhan, L. G., Droser, M. L., Planavsky, N. J. & Johnston, D. T. Protracted development of bioturbation through the early Palaeozoic Era. *Nat. Geosci.* **8**, 865–869 (2015).

[44] Cribb, A.T. & Darroch, S.A.F. How to engineer a habitable planet: the rise of marine ecosystem engineers through the Phanerozoic. *Palaeontology* **67**, 10.1111/pala.12726 (2024).

[45] Wild, C. et al. Climate change impedes scleractinian corals as primary reef ecosystem engineers. *Mar. Freshw. Res.* **62**, 205–215 (2011).

[46] Monismith, S. G. Hydrodynamics of coral reefs. *Annu. Rev. Fluid Mech.* **39**, 37–55 (2007).

[47] Close, R.A., Benson, R.J., Kiessling, W. & Saupe, E.E. Reefal regions were biodiversity hotspots throughout the Phanerozoic. *Sci. Adv.* **11**, 10.1126/sciadv.adv9793 (2025). PMC12588287 41191763

[48] Schobben, M. et al. A nutrient control on marine anoxia during the end-Permian mass extinction. *Nat. Geosci.* **13**, 640–646 (2020).

[49] Hülse, D. et al. End-Permian marine extinction due to temperature-driven nutrient recycling and euxinia. *Nat. Geosci.* **14**, 862–867 (2021).

[50] Flugel, E. & Kiessling, W. Patterns of Phanerozoic Reef Crises. In Phanerozoic Reef Patterns (SEPM (Society for Sedimentary Geology)), pp. 691–733. 10.2110/pec.02.72.0691 (2002).

[51] Reddin, C. J., Kocsis, ÁT., Aberhan, M. & Kiessling, W. Victims of ancient hyperthermal events herald the fates of marine clades and traits under global warming. *Glob. Chang. Biol.* **27**, 868–878 (2021). 33230883 10.1111/gcb.15434

[52] Cribb, A.T., van de Velde, S.J., Berelson, W.M., Bottjer, D.J. & Corsetti, F.A. Ediacaran–Cambrian bioturbation did not extensively oxygenate sediments in shallow marine ecosystems. *Geobiology* **21**, 10.1111/gbi.12550 (2023). 36815223

[53] Foster, W.J., & Twitchett, R.J. Functional diversity of marine ecosystems after the Late Permian mass extinction event. **23**, 10.1038/NGEO2079 (2014).

[54] Boag, T. H., Gearty, W. & Stockey, R. G. Metabolic tradeoffs control biodiversity gradients through geological time. *Curr. Biol.* **31**, 2906–2913.e3 (2021). 33961786 10.1016/j.cub.2021.04.021

[55] Kocsis, ÁT., Reddin, C. J., Alroy, J. & Kiessling, W. The R package divDyn for quantifying diversity dynamics using fossil sampling data. *Methods Ecol. Evol.* **10**, 735–743 (2019).

[56] Lee, J., Dattilo, B. F., Mrozek, S., Miller, J. F. & Riding, R. Lithistid sponge-microbial reefs, Nevada, USA: Filling the late Cambrian “reef gap. *Palaeogeogr. Palaeoclimatol. Palaeoecol.* **520**, 251–262 (2019).

[57] Kristensen, E. et al. What is bioturbation? The need for a precise definition for fauna in aquatic sciences. *Mar. Ecol. Prog. Ser.* **446**, 285–302 (2012).

[58] Kiessling, W. & Flügel, E. Paleoreefs–A database on Phanerozoic reefs. In *Phanerozoic Reef Patterns*, Kiessling, W., Flugel, E. & Golonka, J. eds. (SEPM (Society for Sedimentary Geology)), pp. 77–92 10.2110/pec.02.72.0077 (2002).

[59] Guy-Haim, T. et al. Diverse effects of invasive ecosystem engineers on marine biodiversity and ecosystem functions: A global review and meta-analysis. *Glob. Chang. Biol.* **24**, 906–924 (2018). 29211336 10.1111/gcb.14007

[60] Cohen, J. Statistical Power Analysis for the Behavioral Sciences (Routledge) 10.4324/9780203771587. (1988).

[61] Durlak, J. A. How to select, calculate, and interpret effect sizes. *J. Pediatr. Psychol.* **34**, 917–928 (2009). 19223279 10.1093/jpepsy/jsp004

[62] Scotese, C. R., Song, H., Mills, B. J. W. & van der Meer, D. G. Phanerozoic paleotemperatures: The Earth’s changing climate during the last 540 million years. *Earth-Sci. Rev.* **215**, 103503 (2021).

[63] Allen, A. P., Brown, J. H. & Gillooly, J. F. Global biodiversity, biochemical kinetics, and the energetic-equivalence rule. *Science* **297**, 1545–1548 (2002). 12202828 10.1126/science.1072380

[64] R. Core Team. R: A language environment for statistical computing. at R Foundation for Statistical Computing. (2022).

[65] Wickham, H., Vaughan, D. & Girlich, M. tidyr: Tidy Messy Data. (2024).

[66] Jones, L. A. et al. palaeoverse: A community-driven R package to support palaeobiological analysis. *Methods Ecol. Evol.* **14**, 2205–2215 (2023).

[67] Hijmans, J., Brown, A. & Barbosa, M. Terra: Spatial Data Analysis. *R. Package Version* **1**, 8–96 (2026).

[68] Wang, X. fANCOVA: Nonparametric analysis of covariance. (2020).

[69] Wickham, H. ggplot2: Elegant Graphics for Data Analysis (Springer-Verlag). (2016).

[70] Gearty, W. Deeptime: an R package that facilitates highly customizable visualizations of data over geological time intervals. *Earth ArXiv*. 10.31223/X5841N (2024).

[71] Ram, K. & Wickham, H. Wesanderson: A Wes Anderson Palette Generator. (2023).

