# Supplementary material for: Marine biodiversity was greater in the presence of ecosystem engineers throughout the Phanerozoic
Source: Nat Commun. 2026 Jul 28;17:9166. doi: 10.1038/s41467-026-73628-6 (PMC13522536; doi:10.1038/s41467-026-73628-6)
Supplement: Supplementary file 1 [file 41467_2026_73628_MOESM1_ESM.pdf]

Supplementary materials for

Marine biodiversity was greater in the presence of ecosystem engineers throughout the Phanerozoic

Alison T. Cribb, Simon A.F. Darroch, William Gearty

*corresponding author: A.T.Cribb@soton.ac.uk

Table of contents

Table S1. Comparison of Shannon's Diversity in formations with (H_{BP}) and without (H_{BA}) bioturbators	……………………………..	3
Table S2. Results of effect size analysis for the presence of bioturbators	……………………………..	6
Table S3. Comparison of Shannon's Diversity (H) in formations with (H_{RA}) and without (H_{RP}) reef-builders	……………………………..	9
Table S4. Results of effect size analysis for the presence of reef-builders	……………………………..	12
Table S5. Bioturbator and reef-builder fossil occurrences in each stage and their inclusion in ecosystem engineer diversity calculations	……………………………..	15
Figure S1. Comparison of effect sizes with ecosystem engineers kept versus removed from their formations in diversity analyses	……………………………..	18
Figure S2. Distribution of palaeoenvironments in formations where bioturbators are present and absent	……………………………..	19
Figure S3. Effect sizes of bioturbators on Shannon's Diversity (H) constrained within equal palaeoenvironments	……………………………..	20
Figure S4. Distribution of palaeoenvironments of formations where reef-builders are present and absent	……………………………..	21
Figure S5. Effect sizes of reef-builders on Shannon's Diversity (H) constrained within equal palaeoenvironments	……………………………..	22
Figure S6. Sampling biases impacting bioturbator effect sizes	……………………………..	23
Figure S7. Sampling biases impacting reef-builder effect sizes	……………………………..	24
Figure S8. Equal-area grid cell spatiotemporal subsampling results for bioturbator effect sizes	……………………………..	25
Figure S9. Equal-area grid cell spatiotemporal subsampling results for reef-builder effect sizes	……………………………..	26
Figure S10. Comparison of effect sizes calculated for formation-based versus equal area grid cell-based spatial subsampling approaches	……………………………..	27
Figure S11. Comparison of threshold test effect size results with full dataset effect size results	……………………………..	28

Supplementary Table 1 | Comparison of Shannon's Diversity in formations with (H_{BP}) and without (H_{BA}) bioturbators

Period	Stage	Age (Ma)	*n1*	*n2*	H_{BP}	H_{BA}
Cambrian	Fortunian	533.90	17	18	1.56 ± 0.09	0.59 ± 0.09
	Stage 2	525.00	23	14	1.34 ± 0.08	0.76 ± 0.12
	Stage 3	517.75	41	70	1.12 ± 0.08	0.78 ± 0.07
	Stage 4	511.75	28	76	1.13 ± 0.10	0.86 ± 0.05
	Wulian	506.75	23	64	1.34 ± 0.09	0.82 ± 0.07
	Drumian	502.50	13	38	1.53 ± 0.07	1.02 ± 0.06
	Guzhangian	498.75	13	34	1.49 ± 0.13	0.78 ± 0.07
	Paibian	495.60	12	26	1.44 ± 0.09	1.06 ± 0.08
	Jiangshanian	492.60	16	29	1.54 ± 0.12	1.11 ± 0.10
	Stage 10	488.93	16	26	1.24 ± 0.10	0.88 ± 0.08
Ordovician	Tremadocian	481.97	52	114	1.22 ± 0.07	0.71 ± 0.05
	Floian	474.17	33	81	1.19 ± 0.10	0.93 ± 0.06
	Dapingian	470.34	20	80	1.37 ± 0.14	0.83 ± 0.07
	Darriwilian	463.80	63	132	1.11 ± 0.07	0.72 ± 0.05
	Sandbian	455.47	117	179	1.00 ± 0.05	0.59 ± 0.05
	Katian	448.98	117	260	1.05 ± 0.06	0.43 ± 0.04
	Hirnantian	444.14	20	68	1.37 ± 0.10	0.92 ± 0.07
Silurian	Rhuddanian	441.78	5	47	1.29 ± 0.17	1.27 ± 0.06
	Aeronian	439.54	9	61	1.29 ± 0.14	1.03 ± 0.06
	Telychian	435.76	20	92	1.53 ± 0.10	0.92 ± 0.06
	Sheinwoodian	431.78	13	44	1.86 ± 0.14	1.08 ± 0.08
	Homerian	428.68	6	44	2.24 ± 0.13	1.01 ± 0.07
	Gorstian	425.88	7	34	2.09 ± 0.15	1.12 ± 0.09
	Ludfordian	423.87	14	50	1.65 ± 0.14	0.92 ± 0.07
	Pridoli	420.87	23	76	1.35 ± 0.08	0.88 ± 0.07
Devonian	Lochkovian	415.70	33	99	1.11 ± 0.09	0.85 ± 0.05
	Pragian	411.46	20	96	1.71 ± 0.11	0.79 ± 0.05
	Emsian	402.41	53	158	1.12 ± 0.08	0.68 ± 0.05
	Eifelian	389.80	27	128	1.16 ± 0.10	0.71 ± 0.05
	Givetian	382.10	61	162	1.16 ± 0.09	0.42 ± 0.05
	Frasnian	375.00	46	155	1.14 ± 0.08	0.73 ± 0.05
	Famennian	365.20	34	109	0.95 ± 0.09	0.70 ± 0.05
Carboniferous	Tournaisian	353.02	26	127	1.37 ± 0.10	0.78 ± 0.05
	Visean	338.54	63	180	1.05 ± 0.07	0.53 ± 0.05
	Serpukhovian	326.87	29	56	1.39 ± 0.09	0.78 ± 0.07
	Bashkirian	319.28	31	54	1.50 ± 0.08	0.81 ± 0.08
	Moscovian	311.09	55	70	1.38 ± 0.06	0.59 ± 0.07
	Kasimovian	305.35	41	38	1.32 ± 0.08	0.66 ± 0.08

	Gzhelian	301.29	42	42	1.31 ± 0.08	0.56 ± 0.08
Permian	Asselian	296.21	39	79	1.11 ± 0.09	0.69 ± 0.06
	Sakmarian	292.02	59	113	1.02 ± 0.07	0.60 ± 0.06
	Artinskian	286.91	48	151	1.21 ± 0.08	0.57 ± 0.05
	Kungurian	278.84	83	111	1.02 ± 0.07	0.56 ± 0.08
	Roadian	271.79	66	88	1.16 ± 0.07	0.51 ± 0.07
	Wordian	266.78	68	87	1.16 ± 0.06	0.61 ± 0.07
	Capitanian	261.95	44	70	1.22 ± 0.09	0.71 ± 0.08
	Wuchiapingian	256.90	74	76	1.29 ± 0.08	0.40 ± 0.09
	Changhsingian	253.07	61	37	1.16 ± 0.08	0.64 ± 0.12
Triassic	Induan	250.89	42	34	1.21 ± 0.08	0.80 ± 0.10
	Olenekian	248.29	64	63	1.02 ± 0.07	0.83 ± 0.08
	Anisian	244.08	72	97	1.03 ± 0.07	0.66 ± 0.07
	Ladinian	239.23	50	74	1.23 ± 0.08	0.67 ± 0.06
	Carnian	232.15	79	74	0.92 ± 0.07	0.53 ± 0.07
	Norian	218.41	79	67	1.09 ± 0.06	0.51 ± 0.07
	Rhaetian	205.44	53	30	1.10 ± 0.07	0.66 ± 0.09
Jurassic	Hettangian	200.41	35	22	1.20 ± 0.07	0.69 ± 0.10
	Sinemurian	196.18	49	39	1.13 ± 0.07	0.65 ± 0.08
	Pliensbachian	188.55	66	61	1.24 ± 0.07	0.65 ± 0.07
	Toarcian	179.45	56	48	1.04 ± 0.08	0.85 ± 0.09
	Aalenian	172.80	21	26	1.38 ± 0.12	0.60 ± 0.10
	Bajocian	169.54	53	52	1.02 ± 0.07	0.58 ± 0.07
	Bathonian	166.73	54	44	1.26 ± 0.08	0.57 ± 0.09
	Callovian	163.41	50	43	1.15 ± 0.09	0.45 ± 0.10
	Oxfordian	158.16	82	66	1.14 ± 0.06	0.64 ± 0.08
	Kimmeridgian	152.01	45	53	1.26 ± 0.07	0.55 ± 0.07
	Tithonian	146.17	55	66	1.04 ± 0.07	0.59 ± 0.08
Cretaceous	Berriasian	140.40	36	35	0.95 ± 0.07	0.81 ± 0.07
	Valanginian	135.15	46	45	0.99 ± 0.08	0.97 ± 0.08
	Hauterivian	129.55	40	28	1.17 ± 0.08	0.81 ± 0.09
	Barremian	123.95	46	34	1.16 ± 0.07	0.84 ± 0.08
	Aptian	117.30	86	65	1.09 ± 0.06	0.56 ± 0.07
	Albian	106.85	115	93	0.93 ± 0.05	0.52 ± 0.06
	Cenomanian	97.20	124	88	0.90 ± 0.05	0.51 ± 0.07
	Turonian	91.65	99	61	1.00 ± 0.06	0.43 ± 0.08
	Coniacian	87.55	40	37	1.15 ± 0.07	0.57 ± 0.06
	Santonian	84.68	55	36	1.17 ± 0.07	0.68 ± 0.10
	Campanian	77.91	148	81	0.91 ± 0.05	0.53 ± 0.08
	Maastrichtian	69.11	113	81	1.09 ± 0.08	0.32 ± 0.10
Paleogene	Danian	63.85	62	18	1.18 ± 0.08	0.21 ± 0.15
	Selandian-Thanetian	58.83	89	22	1.09 ± 0.05	0.48 ± 0.13

	Ypresian	52.04	97	37	1.08 ± 0.06	0.48 ± 0.12
	Lutetian	44.55	97	35	1.09 ± 0.06	0.27 ± 0.10
	Bartonian	39.37	79	42	1.12 ± 0.08	0.13 ± 0.12
	Priabonian	35.81	144	64	0.97 ± 0.05	0.50 ± 0.09
	Rupelian	30.60	102	35	1.14 ± 0.06	0.25 ± 0.12
	Chattian	25.17	118	48	1.12 ± 0.05	0.59 ± 0.09
Neogene	Lower Miocene	19.52	190	83	0.88 ± 0.05	0.30 ± 0.08
	Middle Miocene	13.81	173	102	0.91 ± 0.05	0.27 ± 0.07
	Upper Miocene	8.48	163	80	0.91 ± 0.05	0.35 ± 0.10
	Pliocene	3.96	214	85	0.95 ± 0.05	0.22 ± 0.09
Quaternary	Pleistocene	1.30	180	72	0.94 ± 0.05	0.43 ± 0.11
	Holocene	0.01	10	8	1.75 ± 0.13	0.95 ± 0.13

Diversity metrics for formations with and without bioturbating ecosystem engineers for each stage in the Phanerozoic. *n1* = number of formations with at least one bioturbating ecosystem engineer present; *n2* = number of formations without any bioturbating ecosystem engineer genera. Shannon's Diversity given as mean value of 1000 subsampling iterations ± 1 standard deviation. H_{BP} = mean Shannon's Diversity across formations where bioturbators are present; H_{BA} = mean Shannon's Diversity across formations where bioturbators are absent. Stage names and mid-point ages correspond to those used in Kocsis et al.'s (2019) divDyn package in R.

Supplementary Table 2 | Results of effect size analysis for the presence of bioturbators

Period	Stage	Age (Ma)	Hedges' g: Shannon's Diversity	
Cambrian	Fortunian	533.90	1.40 ± 0.26	***
	Stage 2	525.00	0.82 ± 0.23	**
	Stage 3	517.75	0.45 ± 0.15	*
	Stage 4	511.75	0.39 ± 0.16	*
	Wulian	506.75	0.68 ± 0.16	**
	Drumian	502.50	0.84 ± 0.17	**
	Guzhangian	498.75	1.02 ± 0.25	**
	Paibian	495.60	0.55 ± 0.18	*
	Jiangshanian	492.60	0.57 ± 0.21	*
	Stage 10	488.93	0.45 ± 0.17	*
Ordovician	Tremadocian	481.97	0.72 ± 0.13	**
	Floian	474.17	0.32 ± 0.15	
	Dapingian	470.34	0.66 ± 0.20	*
	Darriwilian	463.80	0.55 ± 0.13	*
	Sandbian	455.47	0.55 ± 0.11	*
	Katian	448.98	0.94 ± 0.13	***
	Hirnantian	444.14	0.58 ± 0.16	*
Silurian	Rhuddanian	441.78	0.03 ± 0.24	
	Aeronian	439.54	0.35 ± 0.20	
	Telychian	435.76	0.79 ± 0.16	**
	Sheinwoodian	431.78	0.95 ± 0.22	**
	Homerian	428.68	1.77 ± 0.27	***
	Gorstian	425.88	1.21 ± 0.26	***
	Ludfordian	423.87	0.93 ± 0.23	**
	Pridoli	420.87	0.62 ± 0.15	*
Devonian	Lochkovian	415.70	0.35 ± 0.14	*
	Pragian	411.46	1.28 ± 0.21	***
	Emsian	402.41	0.61 ± 0.13	*
	Eifelian	389.80	0.62 ± 0.17	*
	Givetian	382.10	1.08 ± 0.18	***
	Frasnian	375.00	0.57 ± 0.15	*
	Famennian	365.20	0.32 ± 0.15	
Carboniferous	Tournaisian	353.02	0.77 ± 0.16	**
	Visean	338.54	0.76 ± 0.14	**
	Serpukhovian	326.87	0.81 ± 0.17	**
	Bashkirian	319.28	0.90 ± 0.17	**
	Moscovian	311.09	1.11 ± 0.17	***
	Kasimovian	305.35	0.84 ± 0.17	**
	Gzhelian	301.29	1.00 ± 0.18	***

Permian	Asselian	296.21	0.54 ± 0.15	*
	Sakmarian	292.02	0.59 ± 0.14	*
	Artinskian	286.91	0.91 ± 0.15	**
	Kungurian	278.84	0.56 ± 0.14	*
	Roadian	271.79	0.84 ± 0.15	**
	Wordian	266.78	0.72 ± 0.14	**
	Capitanian	261.95	0.63 ± 0.17	*
	Wuchiapingian	256.90	1.14 ± 0.19	***
	Changhsingian	253.07	0.63 ± 0.19	*
Triassic	Induan	250.89	0.56 ± 0.18	*
	Olenekian	248.29	0.23 ± 0.13	
	Anisian	244.08	0.45 ± 0.13	*
	Ladinian	239.23	0.72 ± 0.15	**
	Carnian	232.15	0.52 ± 0.14	*
	Norian	218.41	0.78 ± 0.15	**
	Rhaetian	205.44	0.55 ± 0.16	*
Jurassic	Hettangian	200.41	0.67 ± 0.17	**
	Sinemurian	196.18	0.65 ± 0.16	*
	Pliensbachian	188.55	0.78 ± 0.16	**
	Toarcian	179.45	0.24 ± 0.15	
	Aalenian	172.80	1.00 ± 0.25	**
	Bajocian	169.54	0.62 ± 0.15	*
	Bathonian	166.73	0.90 ± 0.18	**
	Callovian	163.41	0.86 ± 0.19	**
	Oxfordian	158.16	0.64 ± 0.14	**
	Kimmeridgian	152.01	0.90 ± 0.16	**
	Tithonian	146.17	0.58 ± 0.15	*
Cretaceous	Berriasian	140.40	0.21 ± 0.14	
	Valanginian	135.15	0.03 ± 0.13	
	Hauterivian	129.55	0.49 ± 0.17	*
	Barremian	123.95	0.46 ± 0.15	*
	Aptian	117.30	0.67 ± 0.13	**
	Albian	106.85	0.57 ± 0.12	*
	Cenomanian	97.20	0.54 ± 0.13	*
	Turonian	91.65	0.73 ± 0.15	**
	Coniacian	87.55	0.81 ± 0.15	**
	Santonian	84.68	0.61 ± 0.16	*
	Campanian	77.91	0.47 ± 0.13	*
	Maastrichtian	69.11	0.86 ± 0.16	**
Paleogene	Danian	63.85	1.16 ± 0.23	***
	Selandian-Thanetian	58.83	0.76 ± 0.19	**
	Ypresian	52.04	0.74 ± 0.18	**

	Lutetian	44.55	0.98 ± 0.15	***
	Bartonian	39.37	1.10 ± 0.18	***
	Priabonian	35.81	0.60 ± 0.14	*
	Rupelian	30.60	1.11 ± 0.19	***
	Chattian	25.17	0.66 ± 0.14	**
Neogene	Lower Miocene	19.52	0.73 ± 0.13	**
	Middle Miocene	13.81	0.85 ± 0.13	**
	Upper Miocene	8.48	0.70 ± 0.15	**
	Pliocene	3.96	0.90 ± 0.13	**
Quaternary	Pleistocene	1.30	0.64 ± 0.16	*
	Holocene	0.01	0.80 ± 0.22	**

Hedges' g values for the effect size of bioturbation on Shannon's Diversity in each stage of the Phanerozoic. Hedges' g values are average of 1000 subsampling iterations, with uncertainty given as ±1 standard deviation. Asterisks indicate strength of effect size: none for insignificant effect, * for weak effect ($|\text{Hedges' g} \pm 1\sigma| \geq 0.2$), ** for moderate effect ($|\text{Hedges' g} \pm 1\sigma| \geq 0.5$), and *** for strong effect ($|\text{Hedges' g} \pm 1\sigma| \geq 0.8$). Stage names and mid-point ages correspond to those used in Kocsis et al.'s (2019) divDyn package in R.

Supplementary Table 3 | Comparison of Shannon's Diversity (H) in formations with (H_{RP}) and without (H_{RA}) reef-builders

Period	Stage	Age (mya)	*n1*	*n2*	H_{RP}	H_{RA}
Cambrian	Fortunian	533.90	6	29	1.35 ± 0.21	1.05 ± 0.07
	Stage 2	525.00	4	33	1.58 ± 0.19	1.08 ± 0.08
	Stage 3	517.75	20	91	1.31 ± 0.13	0.83 ± 0.06
	Stage 4	511.75	19	85	1.42 ± 0.10	0.80 ± 0.05
	Wulian	506.75	13	74	1.27 ± 0.16	0.93 ± 0.06
	Drumian	502.50	10	41	1.63 ± 0.08	1.03 ± 0.06
	Guzhangian	498.75	2	45	1.84 ± 0.23	0.94 ± 0.07
	Paibian	495.60	1	37	2.20 ± 0.20	1.16 ± 0.06
	Jiangshanian	492.60	3	42	1.80 ± 0.14	1.23 ± 0.08
	Stage 10	488.93	1	42	2.1 ± 0.19	1.04 ± 0.06
Ordovician	Tremadocian	481.97	11	155	1.18 ± 0.14	0.87 ± 0.05
	Floian	474.17	8	106	1.18 ± 0.25	1.00 ± 0.05
	Dapingian	470.34	8	92	1.24 ± 0.31	0.93 ± 0.06
	Darriwilian	463.80	27	168	1.12 ± 0.11	0.82 ± 0.04
	Sandbian	455.47	55	241	1.18 ± 0.07	0.64 ± 0.05
	Katian	448.98	135	242	0.89 ± 0.05	0.54 ± 0.05
	Hirnantian	444.14	22	66	1.66 ± 0.08	0.77 ± 0.07
Silurian	Rhuddanian	441.78	17	35	1.58 ± 0.07	1.09 ± 0.07
	Aeronian	439.54	24	46	1.41 ± 0.08	0.85 ± 0.07
	Telychian	435.76	40	72	1.49 ± 0.08	0.75 ± 0.07
	Sheinwoodian	431.78	25	32	1.68 ± 0.10	0.90 ± 0.09
	Homerian	428.68	14	36	1.60 ± 0.10	0.98 ± 0.08
	Gorstian	425.88	17	24	1.62 ± 0.12	1.08 ± 0.10
	Ludfordian	423.87	22	42	1.33 ± 0.11	0.96 ± 0.09
	Pridoli	420.87	38	61	1.13 ± 0.07	0.92 ± 0.08
Devonian	Lochkovian	415.70	43	89	1.16 ± 0.07	0.78 ± 0.06
	Pragian	411.46	42	74	1.33 ± 0.08	0.75 ± 0.06
	Emsian	402.41	81	130	1.04 ± 0.06	0.62 ± 0.05
	Eifelian	389.80	72	83	0.96 ± 0.06	0.65 ± 0.06
	Givetian	382.10	116	107	0.86 ± 0.06	0.38 ± 0.06
	Frasnian	375.00	102	99	1.00 ± 0.05	0.59 ± 0.06
	Famennian	365.20	53	90	0.98 ± 0.08	0.65 ± 0.06
Carboniferous	Tournaisian	353.02	49	104	1.19 ± 0.08	0.75 ± 0.06
	Visean	338.54	123	120	0.85 ± 0.06	0.50 ± 0.06
	Serpukhovian	326.87	38	47	1.14 ± 0.08	0.93 ± 0.08
	Bashkirian	319.28	24	61	1.33 ± 0.11	1.02 ± 0.07
	Moscovian	311.09	39	86	1.20 ± 0.09	0.94 ± 0.07

	Kasimovian	305.35	37	42	1.25 ± 0.08	0.80 ± 0.08
	Gzhelian	301.29	29	55	1.40 ± 0.11	0.77 ± 0.08
Permian	Asselian	296.21	43	75	1.16 ± 0.08	0.65 ± 0.06
	Sakmarian	292.02	42	130	0.96 ± 0.09	0.71 ± 0.05
	Artinskian	286.91	55	144	1.13 ± 0.08	0.61 ± 0.05
	Kungurian	278.84	53	141	1.25 ± 0.08	0.54 ± 0.06
	Roadian	271.79	37	117	1.29 ± 0.12	0.74 ± 0.06
	Wordian	266.78	41	114	1.26 ± 0.10	0.79 ± 0.06
	Capitanian	261.95	37	77	1.42 ± 0.10	0.63 ± 0.07
	Wuchiapingian	256.90	44	106	1.41 ± 0.10	0.67 ± 0.07
	Changhsingian	253.07	30	68	1.46 ± 0.10	0.72 ± 0.09
Triassic	Induan	250.89	12	64	1.37 ± 0.19	0.99 ± 0.07
	Olenekian	248.29	10	117	1.48 ± 0.21	0.88 ± 0.06
	Anisian	244.08	20	149	1.19 ± 0.14	0.79 ± 0.05
	Ladinian	239.23	18	106	1.26 ± 0.12	0.86 ± 0.06
	Carnian	232.15	40	113	1.08 ± 0.11	0.63 ± 0.06
	Norian	218.41	45	101	1.22 ± 0.07	0.67 ± 0.06
	Rhaetian	205.44	18	65	1.43 ± 0.13	0.80 ± 0.07
Jurassic	Hettangian	200.41	16	41	1.15 ± 0.11	0.98 ± 0.07
	Sinemurian	196.18	23	65	0.94 ± 0.11	0.95 ± 0.07
	Pliensbachian	188.55	23	104	1.33 ± 0.13	0.91 ± 0.06
	Toarcian	179.45	13	91	1.43 ± 0.15	0.86 ± 0.06
	Aalenian	172.80	9	38	1.85 ± 0.17	0.74 ± 0.08
	Bajocian	169.54	16	89	1.26 ± 0.12	0.74 ± 0.06
	Bathonian	166.73	17	81	1.54 ± 0.14	0.86 ± 0.07
	Callovian	163.41	21	72	1.53 ± 0.13	0.59 ± 0.07
	Oxfordian	158.16	53	95	1.41 ± 0.07	0.57 ± 0.06
	Kimmeridgian	152.01	25	73	1.51 ± 0.10	0.69 ± 0.06
	Tithonian	146.17	27	94	1.40 ± 0.11	0.61 ± 0.06
Cretaceous	Berriasian	140.40	8	63	0.67 ± 0.16	0.90 ± 0.05
	Valanginian	135.15	16	75	1.34 ± 0.12	0.88 ± 0.06
	Hauterivian	129.55	14	54	1.32 ± 0.11	0.94 ± 0.07
	Barremian	123.95	16	64	1.45 ± 0.12	0.92 ± 0.05
	Aptian	117.30	45	106	1.33 ± 0.08	0.63 ± 0.05
	Albian	106.85	60	148	0.92 ± 0.07	0.73 ± 0.05
	Cenomanian	97.20	46	166	1.11 ± 0.09	0.66 ± 0.05
	Turonian	91.65	39	121	1.16 ± 0.10	0.72 ± 0.06
	Coniacian	87.55	18	59	1.09 ± 0.11	0.83 ± 0.05
	Santonian	84.68	33	58	1.11 ± 0.10	0.95 ± 0.07
	Campanian	77.91	64	165	1.12 ± 0.08	0.63 ± 0.05
	Maastrichtian	69.11	76	118	1.30 ± 0.09	0.34 ± 0.07
Paleogene	Danian	63.85	33	47	1.37 ± 0.10	0.77 ± 0.10

	Selandian-Thanetian	58.83	42	69	1.15 ± 0.08	0.90 ± 0.07
	Ypresian	52.04	38	96	1.40 ± 0.09	0.73 ± 0.06
	Lutetian	44.55	39	93	1.41 ± 0.10	0.75 ± 0.06
	Bartonian	39.37	37	84	1.51 ± 0.10	0.58 ± 0.08
	Priabonian	35.81	53	155	1.19 ± 0.09	0.74 ± 0.05
	Rupelian	30.60	37	100	1.29 ± 0.09	0.91 ± 0.07
	Chattian	25.17	50	116	1.26 ± 0.07	0.84 ± 0.06
Neogene	Lower Miocene	19.52	67	206	1.09 ± 0.08	0.62 ± 0.05
	Middle Miocene	13.81	51	224	1.06 ± 0.09	0.70 ± 0.05
	Upper Miocene	8.48	39	204	0.88 ± 0.12	0.81 ± 0.06
	Pliocene	3.96	70	229	1.25 ± 0.08	0.68 ± 0.05
Quaternary	Pleistocene	1.30	82	170	1.15 ± 0.07	0.65 ± 0.06
	Holocene	0.01	6	12	1.52 ± 0.16	1.34 ± 0.12

Diversity metrics for formations with and without metazoan reef-building ecosystem engineers for each stage in the Phanerozoic. Diversity measurements not taken for the metazoan reef gap in Cambrian Stage 10. *n1* = number of formations with at least one reef-building ecosystem engineer present; *n2* = number of formations without any reef-building ecosystem engineer genera. Shannon's Diversity given as mean value of 1000 subsampling iterations ± 1 standard deviation. H_{RP} = mean Shannon's Diversity across formations where reef-builders are present; H_{RA} = mean Shannon's Diversity across formations where reef-builders are absent. Stage names and mid-point ages correspond to those used in Kocsis et al. (2019)'s divDyn package in R.

Supplementary Table 4 | Results of effect size analysis for the presence of reef-builders

Period	Stage	Age (mya)	Hedges' g: Shannon's Diversity	
Cambrian	Fortunian	533.90	0.36 ± 0.27	
	Stage 2	525.00	0.67 ± 0.28	*
	Stage 3	517.75	0.63 ± 0.20	*
	Stage 4	511.75	0.92 ± 0.18	**
	Wulian	506.75	0.42 ± 0.23	
	Drumian	502.50	1.01 ± 0.18	***
	Guzhangian	498.75	1.21 ± 0.33	***
	Paibian	495.60	N/A	
	Jiangshanian	492.60	0.74 ± 0.20	**
	Stage 10	488.93	N/A	
Ordovician	Tremadocian	481.97	0.41 ± 0.20	*
	Floian	474.17	0.21 ± 0.32	
	Dapingian	470.34	0.36 ± 0.37	
	Darriwilian	463.80	0.41 ± 0.17	*
	Sandbian	455.47	0.76 ± 0.13	**
	Katian	448.98	0.50 ± 0.11	*
	Hirnantian	444.14	1.30 ± 0.18	***
Silurian	Rhuddanian	441.78	0.70 ± 0.16	**
	Aeronian	439.54	0.78 ± 0.16	**
	Telychian	435.76	1.02 ± 0.17	***
	Sheinwoodian	431.78	0.97 ± 0.21	**
	Homerian	428.68	0.82 ± 0.19	**
	Gorstian	425.88	0.65 ± 0.21	*
	Ludfordian	423.87	0.45 ± 0.17	*
	Pridoli	420.87	0.28 ± 0.14	
Devonian	Lochkovian	415.70	0.51 ± 0.14	*
	Pragian	411.46	0.78 ± 0.15	**
	Emsian	402.41	0.57 ± 0.11	*
	Eifelian	389.80	0.42 ± 0.12	*
	Givetian	382.10	0.65 ± 0.13	**
	Frasnian	375.00	0.57 ± 0.13	*
	Famennian	365.20	0.45 ± 0.14	*
Carboniferous	Tournaisian	353.02	0.57 ± 0.15	*
	Visean	338.54	0.49 ± 0.12	*
	Serpukhovian	326.87	0.27 ± 0.14	
	Bashkirian	319.28	0.38 ± 0.17	*
	Moscovian	311.09	0.32 ± 0.14	
	Kasimovian	305.35	0.55 ± 0.15	*

	Gzhelian	301.29	0.80 ± 0.19	**
Permian	Asselian	296.21	0.68 ± 0.15	**
	Sakmarian	292.02	0.33 ± 0.14	
	Artinskian	286.91	0.71 ± 0.14	**
	Kungurian	278.84	0.92 ± 0.16	**
	Roadian	271.79	0.69 ± 0.18	**
	Wordian	266.78	0.60 ± 0.15	*
	Capitanian	261.95	1.05 ± 0.21	***
	Wuchiapingian	256.90	0.92 ± 0.18	**
	Changhsingian	253.07	0.94 ± 0.20	**
Triassic	Induan	250.89	0.50 ± 0.29	*
	Olenekian	248.29	0.74 ± 0.28	*
	Anisian	244.08	0.48 ± 0.18	*
	Ladinian	239.23	0.49 ± 0.17	*
	Carnian	232.15	0.59 ± 0.17	*
	Norian	218.41	0.75 ± 0.15	**
	Rhaetian	205.44	0.81 ± 0.21	**
Jurassic	Hettangian	200.41	0.22 ± 0.17	
	Sinemurian	196.18	-0.02 ± 0.17	
	Pliensbachian	188.55	0.54 ± 0.18	*
	Toarcian	179.45	0.72 ± 0.22	**
	Aalenian	172.80	1.50 ± 0.32	***
	Bajocian	169.54	0.72 ± 0.20	**
	Bathonian	166.73	0.85 ± 0.21	**
	Callovian	163.41	1.25 ± 0.25	***
	Oxfordian	158.16	1.18 ± 0.16	***
	Kimmeridgian	152.01	1.04 ± 0.18	***
	Tithonian	146.17	1.09 ± 0.21	***
Cretaceous	Berriasian	140.40	-0.34 ± 0.24	
	Valanginian	135.15	0.58 ± 0.18	*
	Hauterivian	129.55	0.52 ± 0.18	*
	Barremian	123.95	0.76 ± 0.20	**
	Aptian	117.30	0.93 ± 0.15	**
	Albian	106.85	0.26 ± 0.12	
	Cenomanian	97.20	0.61 ± 0.15	*
	Turonian	91.65	0.56 ± 0.16	*
	Coniacian	87.55	0.34 ± 0.16	
	Santonian	84.68	0.19 ± 0.14	
	Campanian	77.91	0.61 ± 0.13	*
	Maastrichtian	69.11	1.18 ± 0.18	***
Paleogene	Danian	63.85	0.71 ± 0.19	**
	Selandian-Thanetian	58.83	0.31 ± 0.14	

	Ypresian	52.04	0.85 ± 0.16	**
	Lutetian	44.55	0.78 ± 0.16	**
	Bartonian	39.37	1.12 ± 0.21	***
	Priabonian	35.81	0.58 ± 0.14	*
	Rupelian	30.60	0.46 ± 0.15	*
	Chattian	25.17	0.52 ± 0.13	*
Neogene	Lower Miocene	19.52	0.59 ± 0.13	*
	Middle Miocene	13.81	0.45 ± 0.13	*
	Upper Miocene	8.48	0.09 ± 0.16	
	Pliocene	3.96	0.71 ± 0.13	**
Quaternary	Pleistocene	1.30	0.65 ± 0.13	**
	Holocene	0.01	0.17 ± 0.19	

Hedges' g values for the effect size of reef-builders on Shannon's Diversity in each stage of the Phanerozoic. Hedges' g values are not possible for the Paibian or Stage 10, where only one formation contains reef-building ecosystem engineers (so σ cannot be measured). Hedges' g values are average of 1000 subsampling iterations, with uncertainty given as ±1 standard deviation. Asterisks indicate strength of effect size: none for insignificant effect, * for weak effect (|Hedges' g ± 1σ| ≥ 0.2), ** for moderate effect (|Hedges' g ± 1σ| ≥ 0.5), and *** for strong effect (|Hedges' g ± 1σ| ≥ 0.80). Stage names and mid-point ages correspond to those used in Kocsis et al. (2019)'s divDyn package in R.

Supplementary Table 5 | Bioturbator and reef-builder fossil occurrences in each stage and their inclusion in ecosystem engineer diversity calculations

period	stage	bioturbator occurrences	bioturbators included in subsampling?	reef occurrences	reefs included in subsampling?
Cambrian	Fortunian	87	no	13	no
	Stage 2	73	no	3	no
	Stage 3	233	yes	71	no
	Stage 4	69	no	62	no
	Wulian	107	no	30	no
	Drumian	16	no	26	no
	Guzhangian	32	no	0	no
	Paibian	16	no	4	no
	Jiangshanian	52	no	1	no
	Stages 10	69	no	0	no
Ordovician	Tremadocian	127	no	10	no
	Floian	80	no	5	no
	Dapingian	58	no	9	no
	Darriwilian	182	yes	43	no
	Sandbian	325	yes	298	yes
	Katian	691	yes	1572	yes
	Hirnantian	26	no	234	yes
Silurian	Rhuddanian	13	no	321	yes
	Aeronian	23	no	241	yes
	Telychian	38	no	471	yes
	Sheinwoodian	26	no	287	yes
	Homerian	22	no	205	yes
	Gorstian	21	no	139	no
	Ludfordian	79	no	199	yes
	Pridoli	76	no	185	yes
Devonian	Lochkovian	63	no	492	yes
	Pragian	48	no	514	yes
	Emsian	242	yes	736	yes
	Eifelian	85	no	1129	yes
	Givetian	1183	yes	2925	yes
	Frasnian	310	yes	1911	yes
	Famennian	84	no	191	yes
Carboniferous	Tournaisian	99	no	391	yes
	Visean	469	yes	1406	yes
	Serpukhovian	200	yes	187	yes
	Bashkirian	273	yes	71	no
	Moscovian	467	yes	141	no
	Kasimovian	158	yes	85	no

	Gzhelian	309	yes	138	no
Permian	Asselian	149	no	337	yes
	Sakmarian	852	yes	291	yes
	Artinskian	221	yes	347	yes
	Kungurian	1074	yes	776	yes
	Roadian	455	yes	159	yes
	Wordian	385	yes	281	yes
	Capitanian	326	yes	222	yes
	Wuchiapingian	1335	yes	320	yes
	Changhsingian	2249	yes	252	yes
Triassic	Induan	1096	yes	9	no
	Olenekian	832	yes	13	no
	Anisian	1067	yes	177	yes
	Ladinian	462	yes	58	no
	Carnian	1439	yes	350	yes
	Norian	762	yes	621	yes
	Rhaetian	866	yes	410	yes
Jurassic	Hettangian	229	yes	45	no
	Sinemurian	366	yes	32	no
	Pliensbachian	1377	yes	45	no
	Toarcian	1299	yes	31	no
	Aalenian	694	yes	65	no
	Bajocian	717	yes	79	no
	Bathonian	1153	yes	230	yes
	Callovian	2059	yes	217	yes
	Oxfordian	1444	yes	929	yes
	Kimmeridgian	755	yes	454	yes
	Tithonian	1083	yes	613	yes
Cretaceous	Berriasian	185	yes	21	no
	Valanginian	477	yes	181	yes
	Hauterivian	728	yes	102	no
	Barremian	273	yes	241	yes
	Aptian	1125	yes	494	yes
	Albian	1608	yes	260	yes
	Cenomanian	3023	yes	398	yes
	Turonian	1685	yes	201	yes
	Coniacian	202	yes	90	no
	Santonian	638	yes	270	yes
	Campanian	3408	yes	561	yes
	Maastrichtian	13000	yes	1177	yes
Paleogene	Danian	3750	yes	247	yes

	Selandian-Thanetian	2182	yes	189	yes
	Ypresian	2548	yes	145	no
	Lutetian	3388	yes	132	no
	Bartonian	4910	yes	450	yes
	Priabonian	2751	yes	251	yes
	Rupelian	4165	yes	439	yes
	Chattian	2054	yes	719	yes
Neogene	Lower Miocene	6253	yes	1019	yes
	Middle Miocene	5066	yes	478	yes
	Upper Miocene	6482	yes	699	yes
	Pliocene	7592	yes	1211	yes
Quaternary	Pleistocene	8879	yes	3144	yes
	Holocene	236	yes	127	no

Number of bioturbator and reef-builder fossil occurrences in each stage. Noted in right hand columns is whether or not the stage is included in Figure 4 analyses for diversity curves and correlates. Stages in which fossil occurrences are less than 150 were not included in the analyses because there is insufficient data to subsampling across the entire Phanerozoic.

Supplementary Figure 1 | Comparison of effect sizes with ecosystem engineers kept versus removed from their formations in diversity analyses

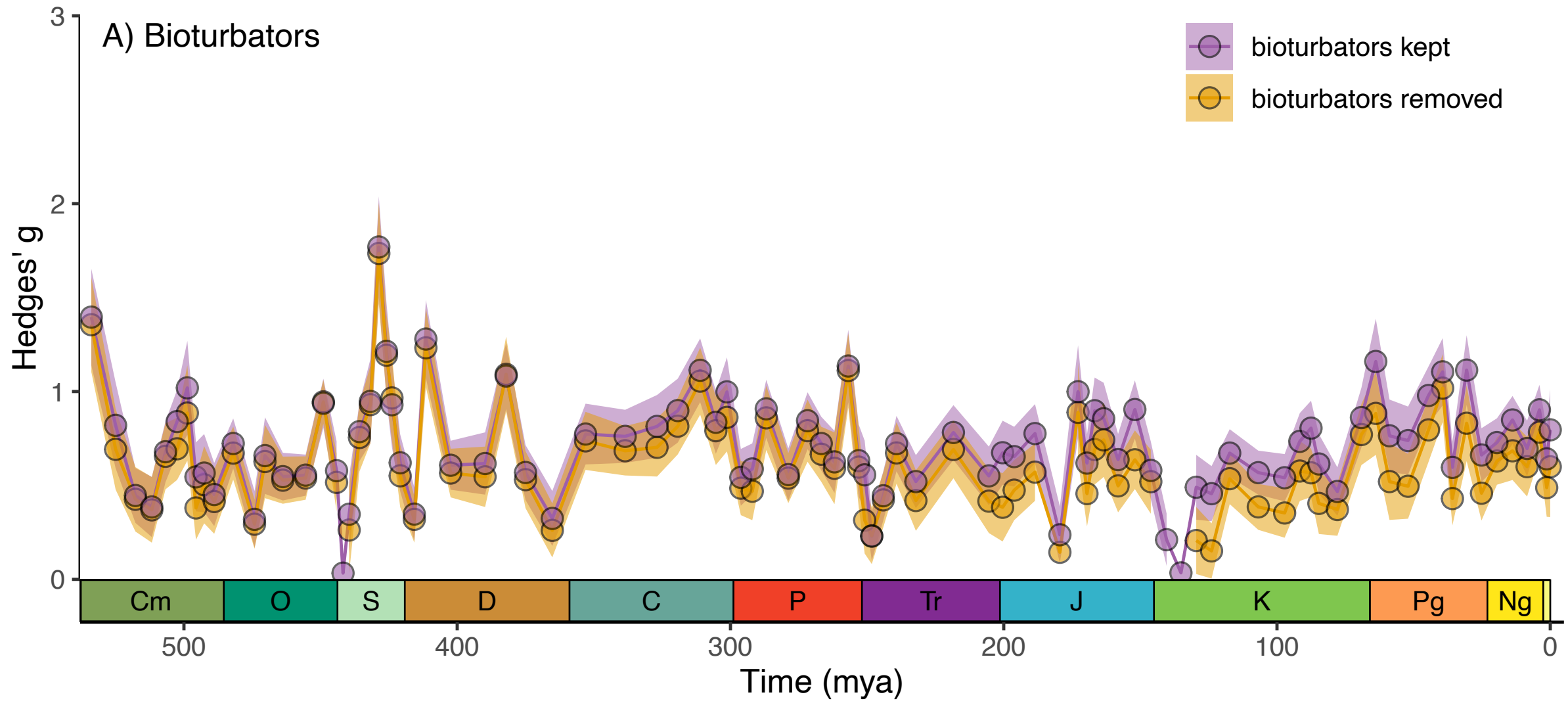

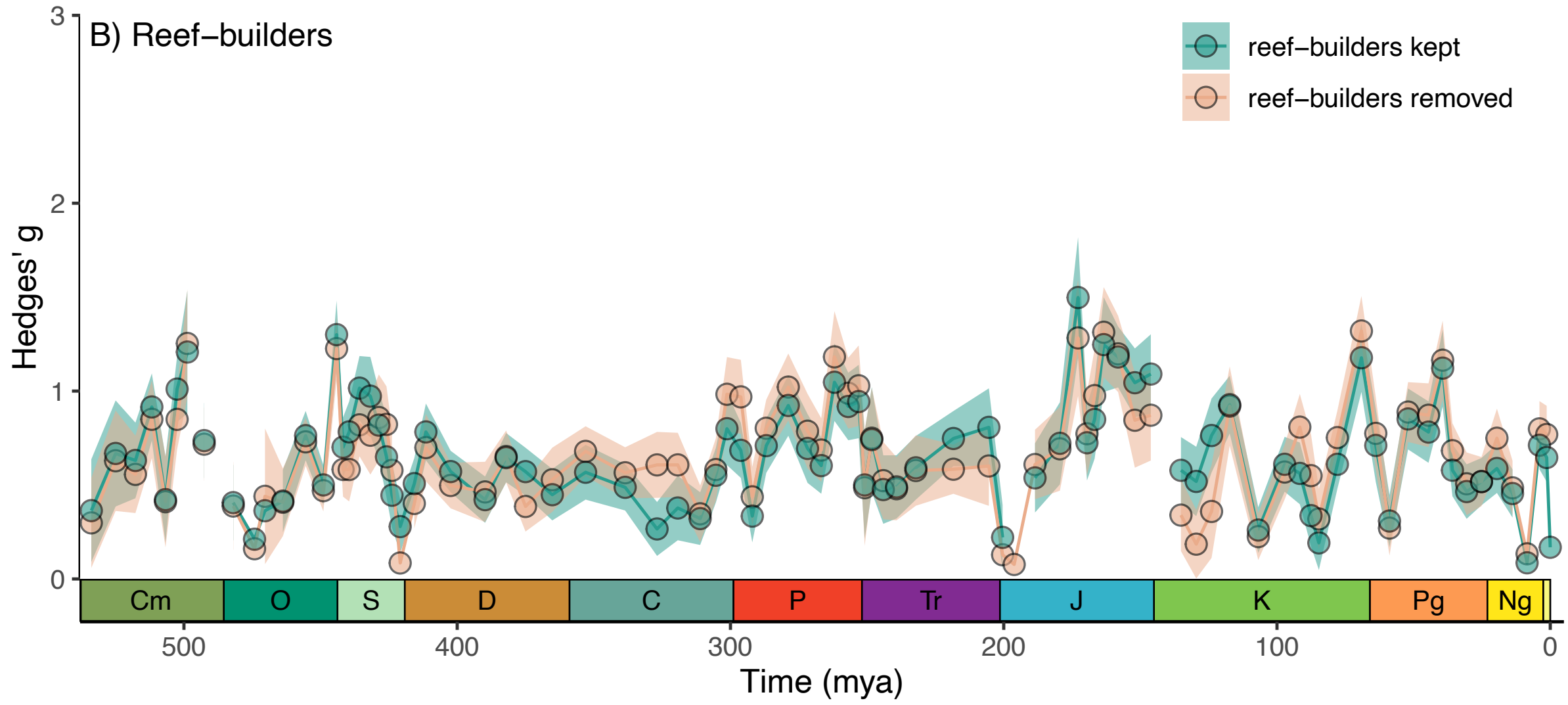

Comparison of effect sizes when A) bioturbators or B) reef-builders are removed from in-formation Shannon's Diversity calculations. Timescale abbreviations are: 'Cm' = Cambrian, 'O' = Ordovician, 'S' = Silurian, 'D' = Devonian, 'C' = Carboniferous, 'P'= Permian, 'Tr'= Triassic, 'J' = Jurassic, 'K' = Cretaceous, 'Pg' = Paleogene, 'Ng' =Neogene. Quaternary is represented by the final two points, but is too small to label on the x-axis timescale. Timescale generated by R package deeptime. Points are average of 1000 subsampling iterations per stage and shaded ribbons are ±1 standard deviation.

Supplementary Figure 2 | Distribution of palaeoenvironments in formations where bioturbators are present and absent

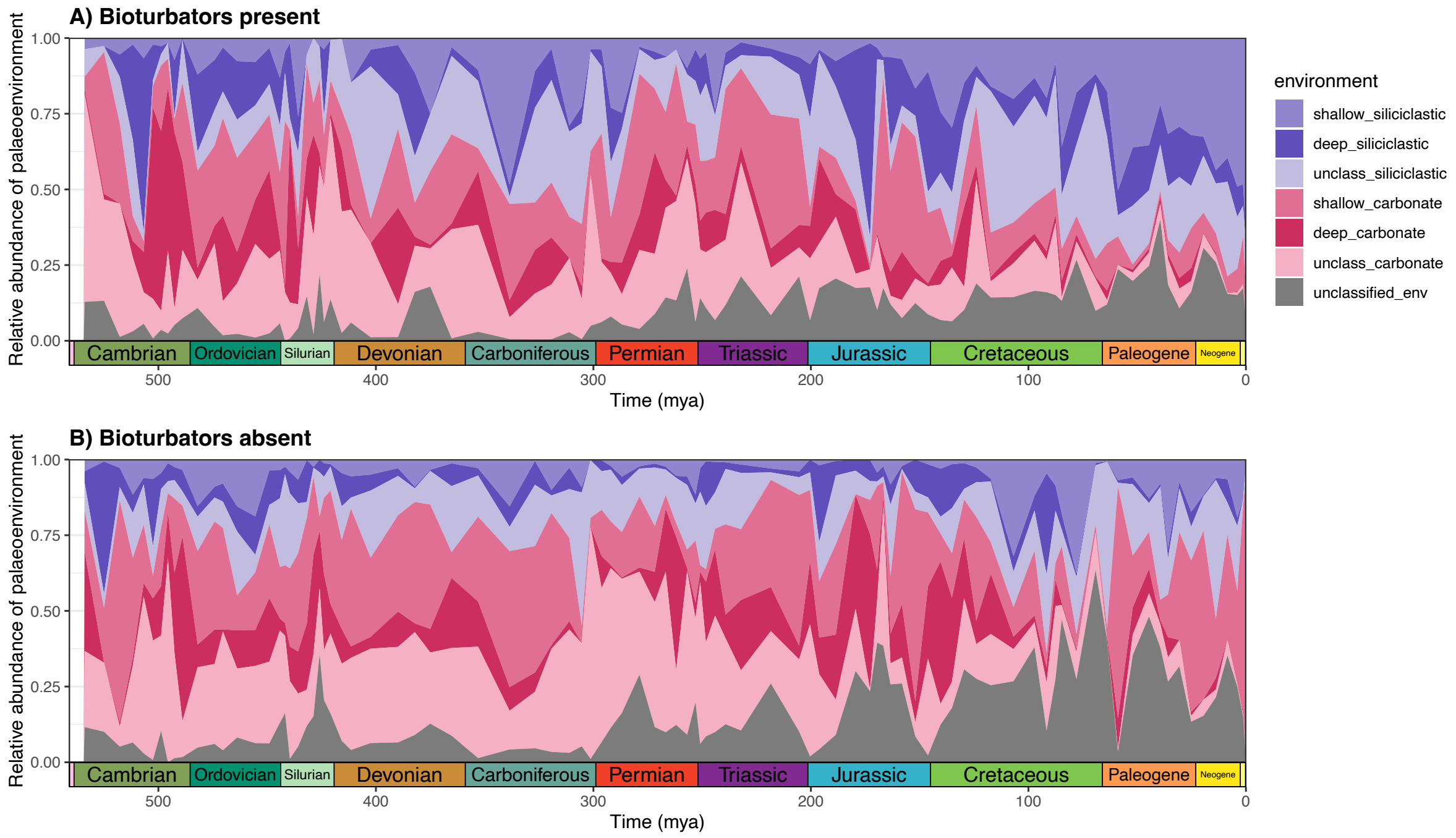

Distribution of palaeoenvironments in formations where bioturbators are present (A) or absent (B) at each stage in the Phanerozoic. Palaeoenvironments are classed as siliciclastic (purples) or carbonates (pinks), and shallow depths (medium), deep depths (dark), or unclassified depths (light). Unclassified environments are grey. Environmental classifications determined based on information in the PBDB following Kocsis et al. (2019).

Supplementary Figure 3 | Effect sizes of bioturbators on Shannon's Diversity (H) constrained within equal palaeoenvironments

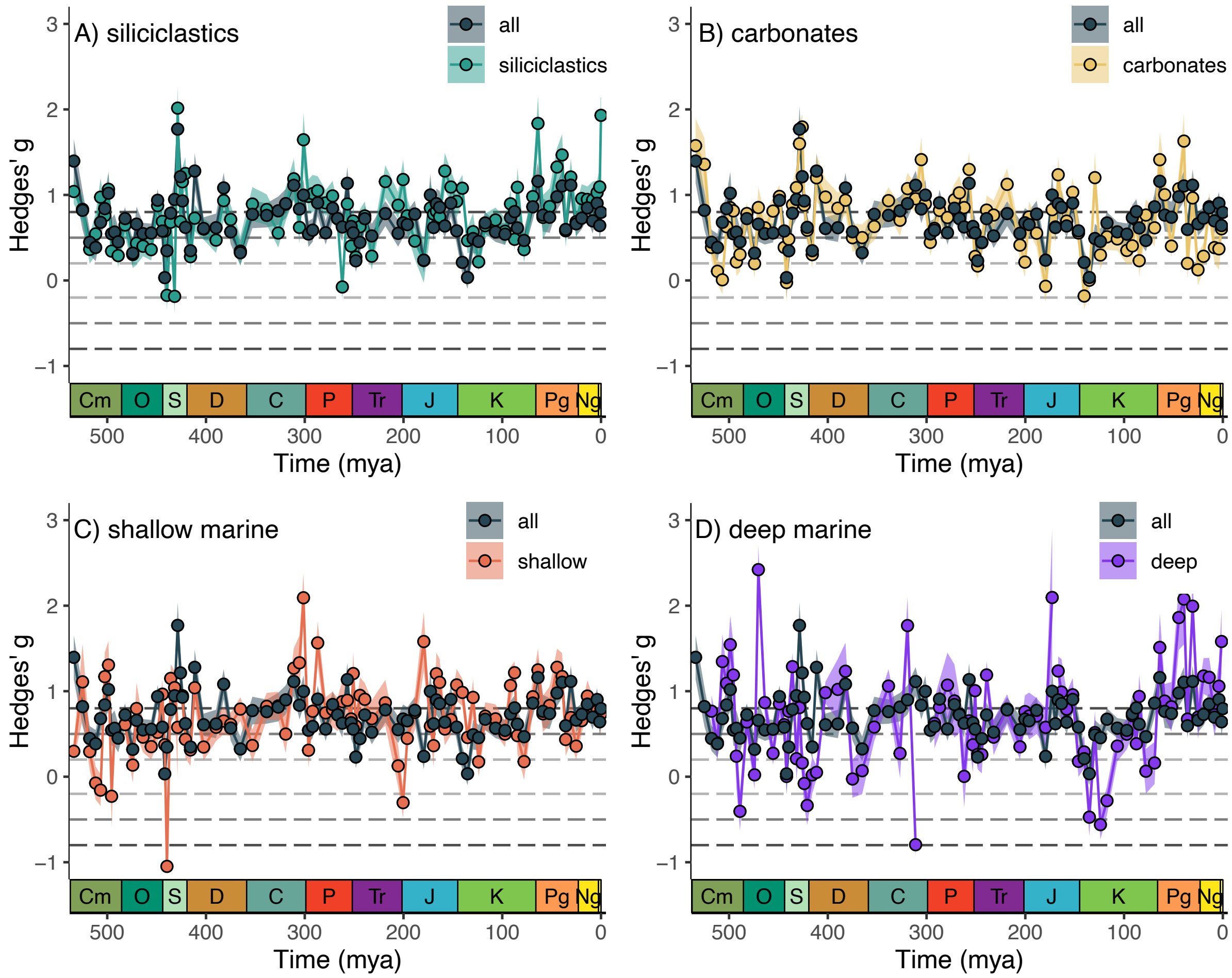

Effect sizes of bioturbators constrained in siliciclastics (A), carbonates (B), shallow depths (C), and deep depths (D). Each effect size analysis is compared to the effect sizes calculated for all environments which are presented in the main text and given on each panel as the dark blue curves. Despite often greater variability when constraining effect sizes to certain environments, positive effect sizes are still present.

Supplementary Figure 4 | Distribution of palaeoenvironments of formations where reef-builders are present and absent

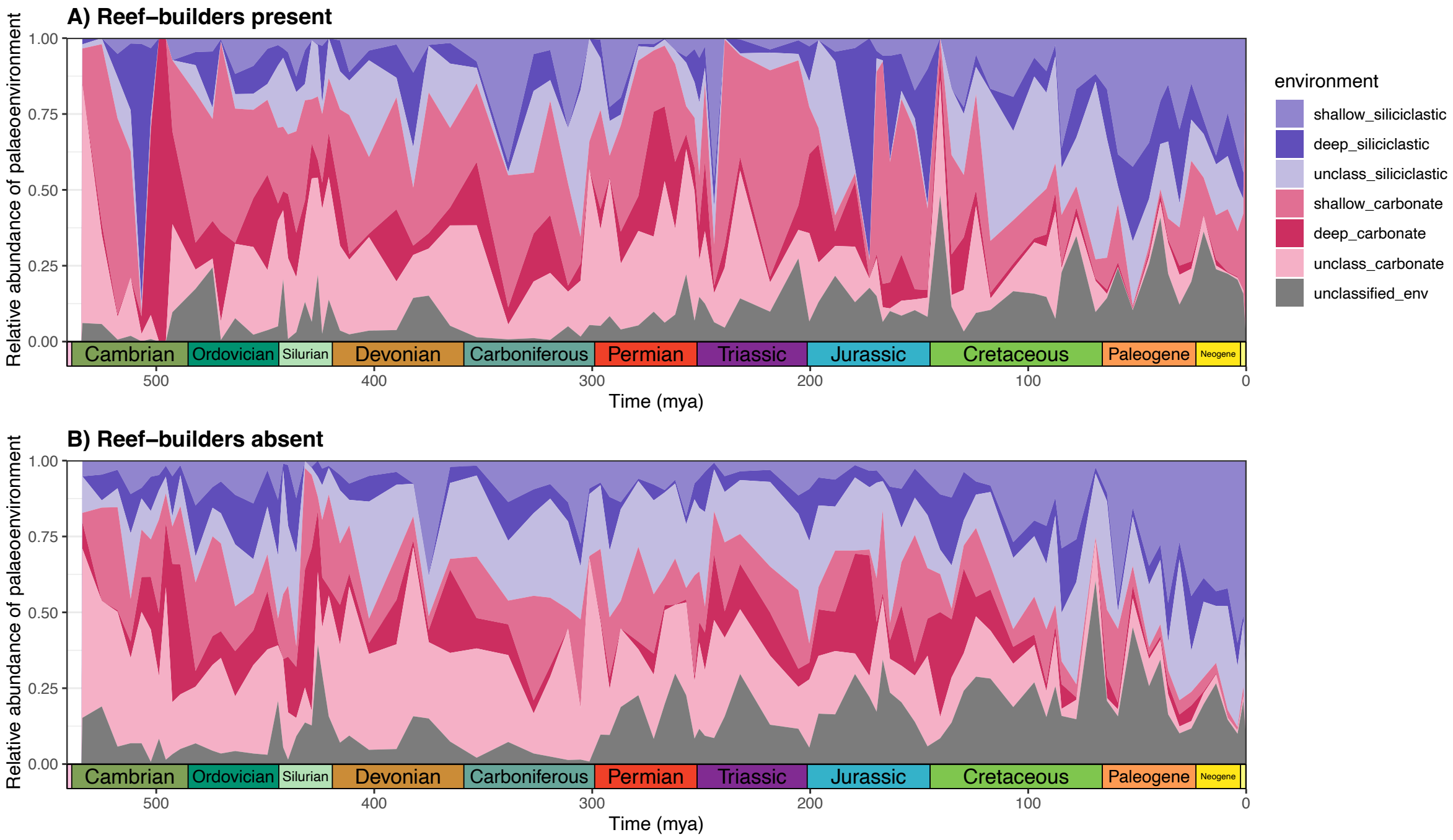

Distribution of palaeoenvironments in formations where reef-builders are present (A) or absent (B) at each stage in the Phanerozoic. Palaeoenvironments are classed as siliciclastic (purples) or carbonates (pinks), and shallow depths (medium), deep depths (dark), or unclassified depths (light). Unclassified environments are grey. Environmental classifications determined based on information in the PBDB following Kocsis et al. (2019).

Supplementary Figure 5 | Effect sizes of reef-builders on Shannon's Diversity (H) constrained within equal palaeoenvironments

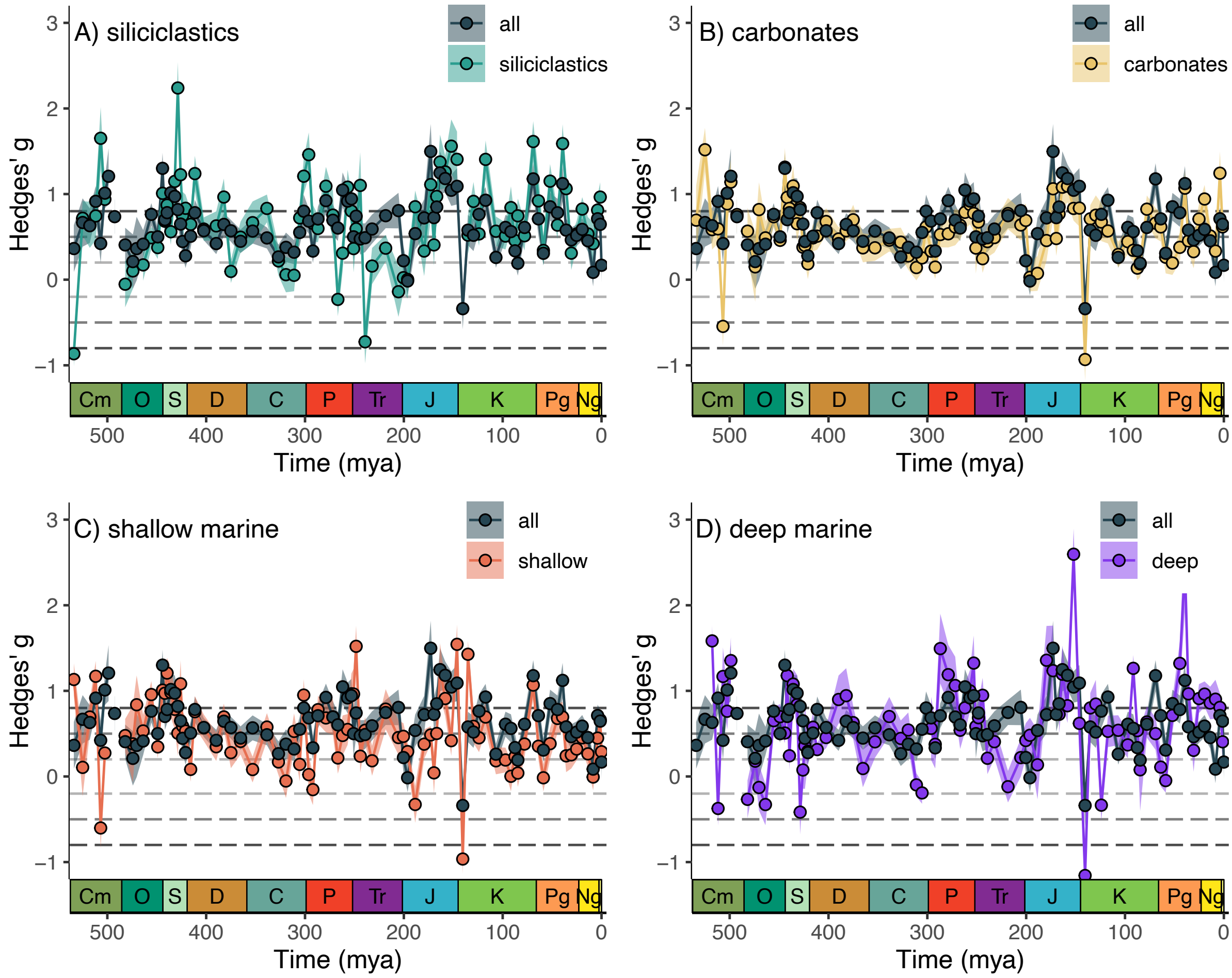

Effect sizes of reef-builders constrained in siliciclastics (A), carbonates (B), shallow depths (C), and deep depths (D). Each effect size analysis is compared to the effect sizes calculated for all environments which are presented in the main text and given on each panel as the dark blue curves. Despite often greater variability when constraining effect sizes to certain environments, positive effect sizes are still present. Points are average *g* of 1000 iteration and shaded coloured areas represent ±1 standard deviation error.

Supplementary Figure 6 | Sampling biases impacting bioturbator effect sizes

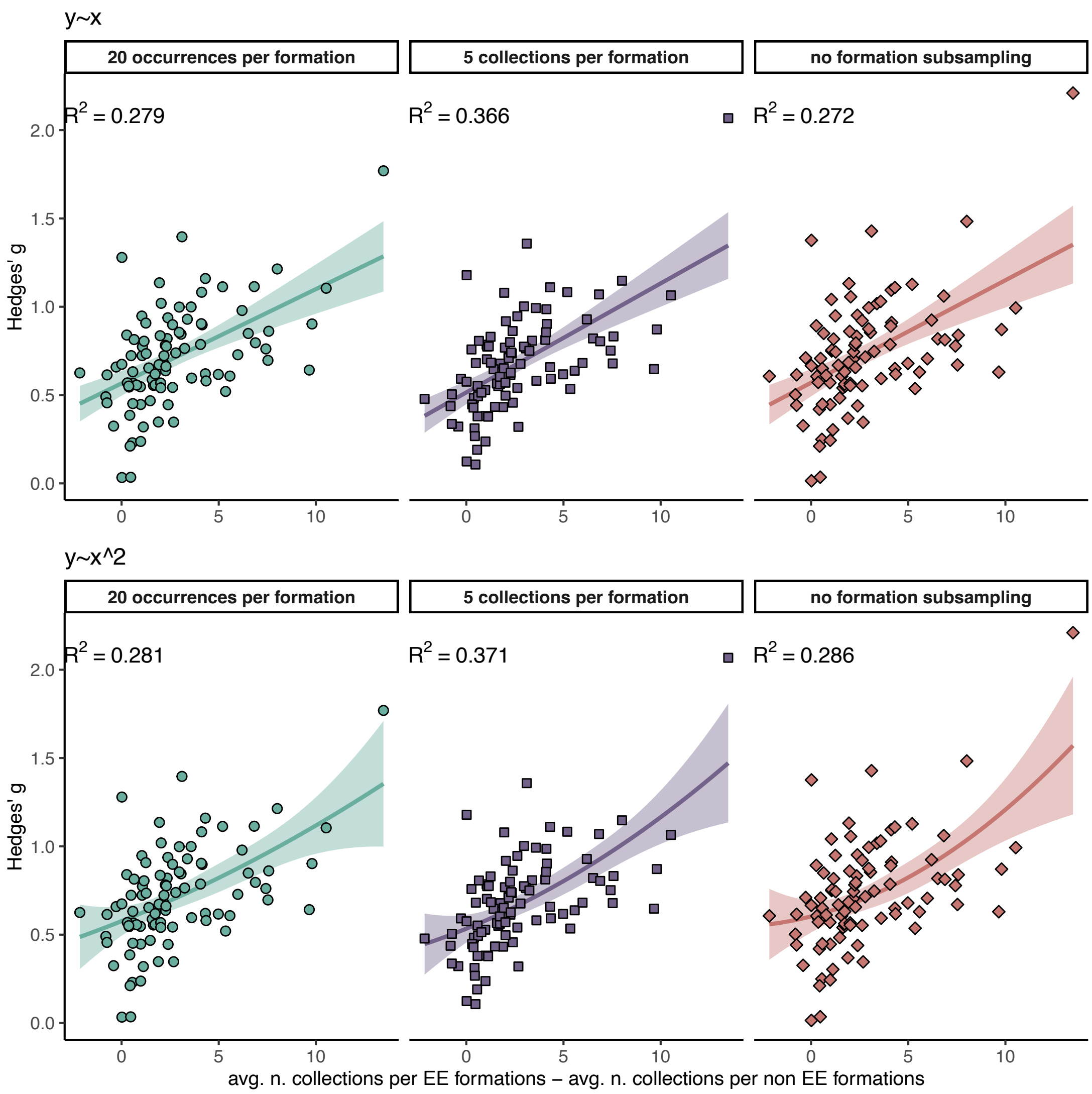

Sampling biases (quantified as the difference between average number of collections in formations with ecosystem engineers and the average number of collections in formations without ecosystem engineers per stage) versus effect size of bioturbators as Hedges' g for *H*. Points are average g for 1000 iterations against the difference between the average number of collections per ecosystem engineer formation and the average number of collections per non-ecosystem engineer formation (the quantified sampling bias). Based on relationships between sampling biases and effect sizes with no formation subsampling, we determined a polynomial fit (effect size ~ g^2) best accounts for this relationship (solid lines are polynomial regression with shaded color areas representing 95% confidence interval of fit). Comparing second order polynomial fits between the two subsampling protocol tests (bottom column), we determined 20 occurrences per formation best minimizes the sampling biases because it has the worst performing fit, or the highest R^2 value (R^2=0.281) versus the alternate method of 5 collections per formation (R^2=0.371).

Supplementary Figure 7 | Sampling biases impacting reef-builder effect sizes

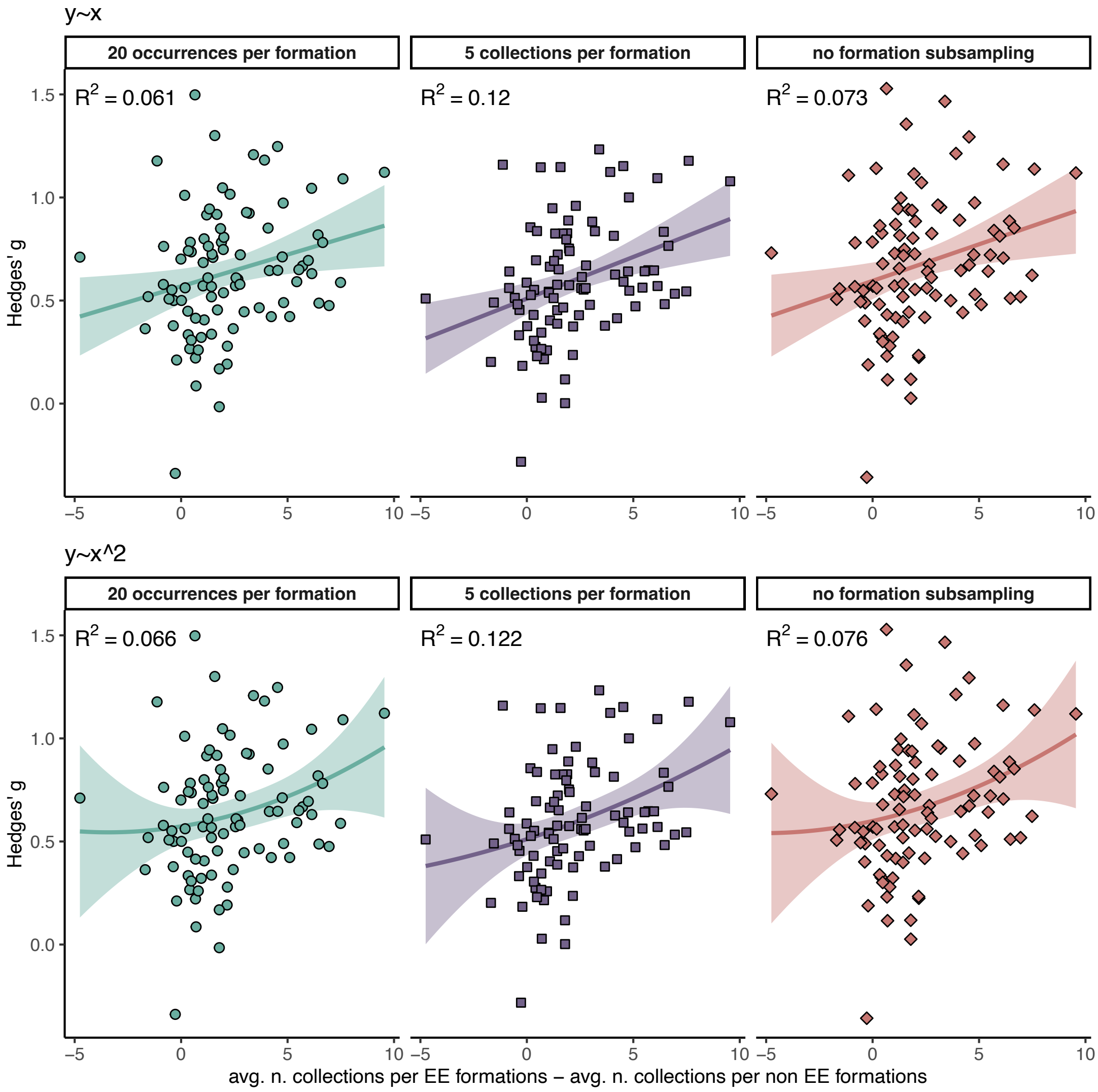

Sampling biases (quantified as the difference between average number of collections in formations with ecosystem engineers and the average number of collections in formations without ecosystem engineers per stage) versus effect size of reef-builders as Hedges' g for *H*. Following the same logic described in the Supplementary Figure 6 caption, we determined 20 occurrences per formation to be the most appropriate subsampling method to minimize sampling biases. Overall, note that within-formation sampling biases much less significantly impact reef-builder effect sizes than bioturbator effect-sizes. Points are average g for 1000 iterations against the difference between the average number of collections per ecosystem engineer formation and the average number of collections per non-ecosystem engineer formation (the quantified sampling bias). For polynomial regression, solid lines are the best polynomial regression fit with shaded color areas representing 95% confidence interval.

Supplementary Figure 8 | Equal-area grid cell spatiotemporal subsampling results for bioturbator effect sizes

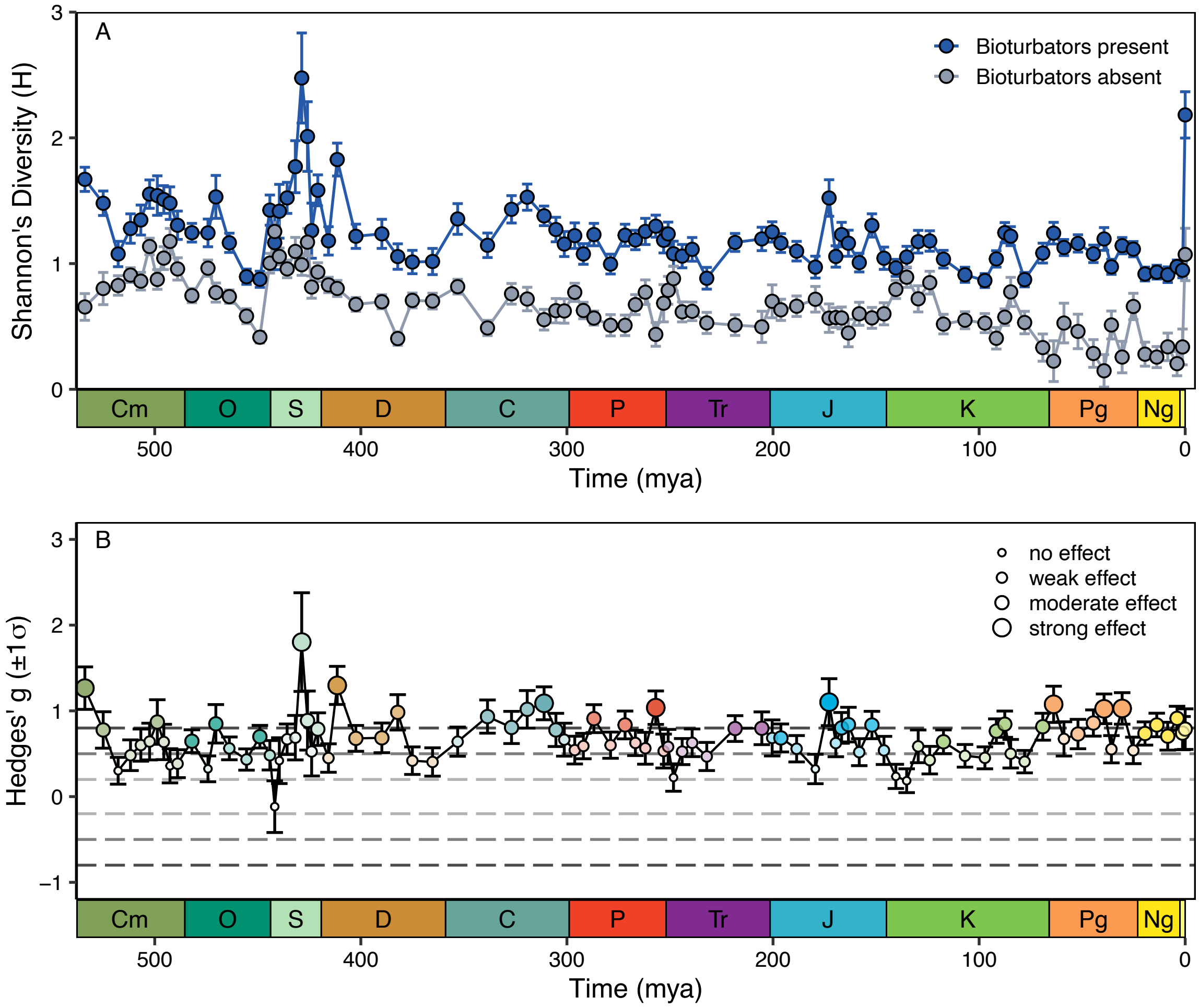

Effect size results following equal-area grid subsampling approach (see *Methods: subsampling protocol* for details) for bioturbators. A) Comparison of *H* in formations where bioturbators are present (blue) versus where they are absent (gray). Points are mean *H* of 1000 iterations with error bars as ±1 standard deviation. B) Effect sizes of the presence of bioturbators for each stage of the Phanerozoic. Points are mean g of 1000 iterations with error bars as ±1 standard deviation. Abbreviations and effect size threshold keys follow Figure 1 in the main text.

Supplementary Figure 9 | Equal-area grid cell spatiotemporal subsampling results for reef-builder effect sizes

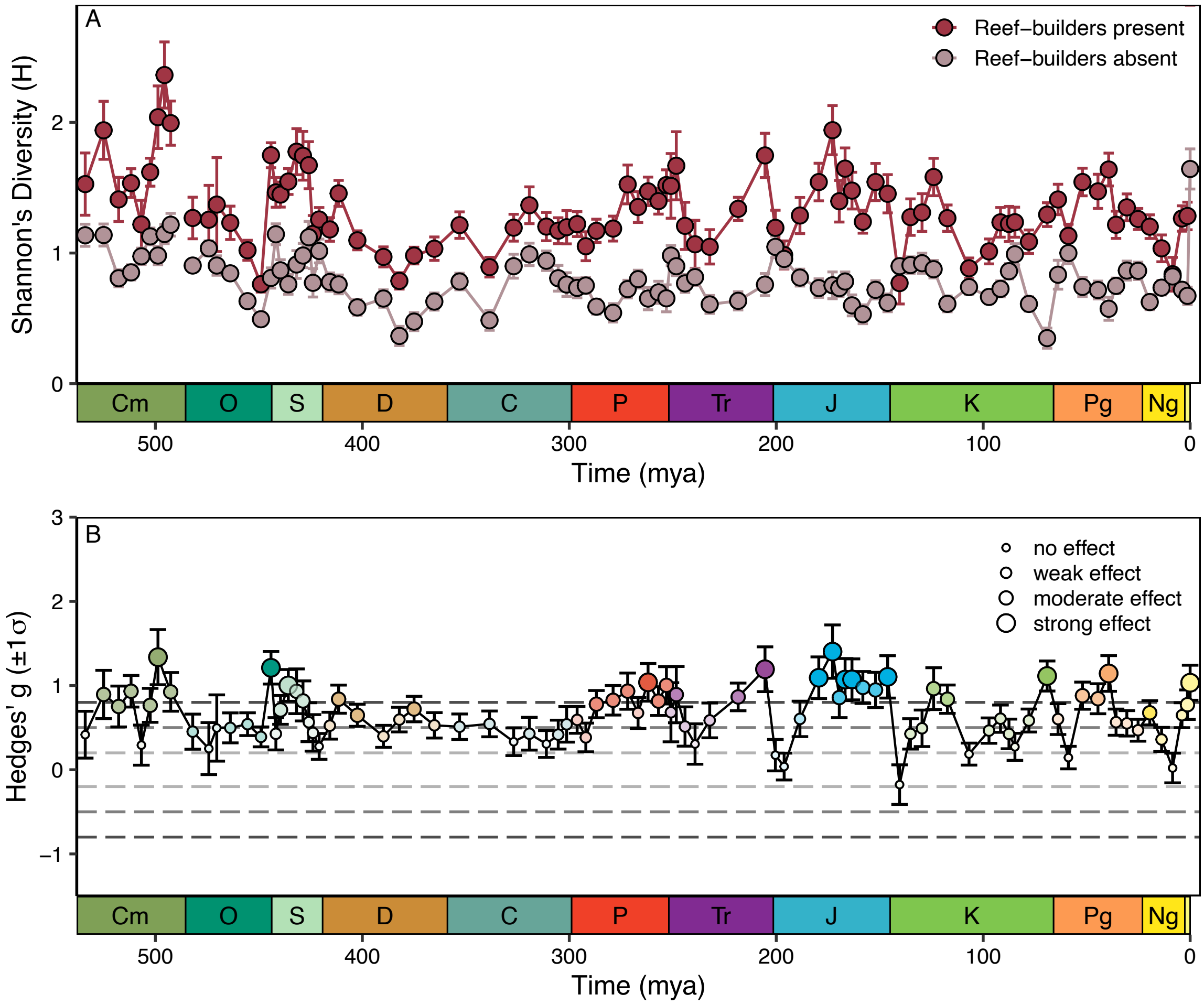

Effect size results following equal-area grid subsampling approach (see *Methods: subsampling protocol* for details) for reef-buildes. A) Comparison of *H* in formations where reef-builders are present (pink) versus where they are absent (brown). Points are average *H* of 1000 iterations with error bars as ±1 standard deviation. B) Effect sizes of the presence of reef-builders for each stage of the Phanerozoic. Points are average *g* of 1000 iterations with error bars as ±1 standard deviation. Abbreviations and effect size threshold keys follow Figure 2 in the main text.

Supplementary Figure 10 | Comparison of effect sizes calculated for formation-based versus equal area grid cell-based spatial subsampling approaches

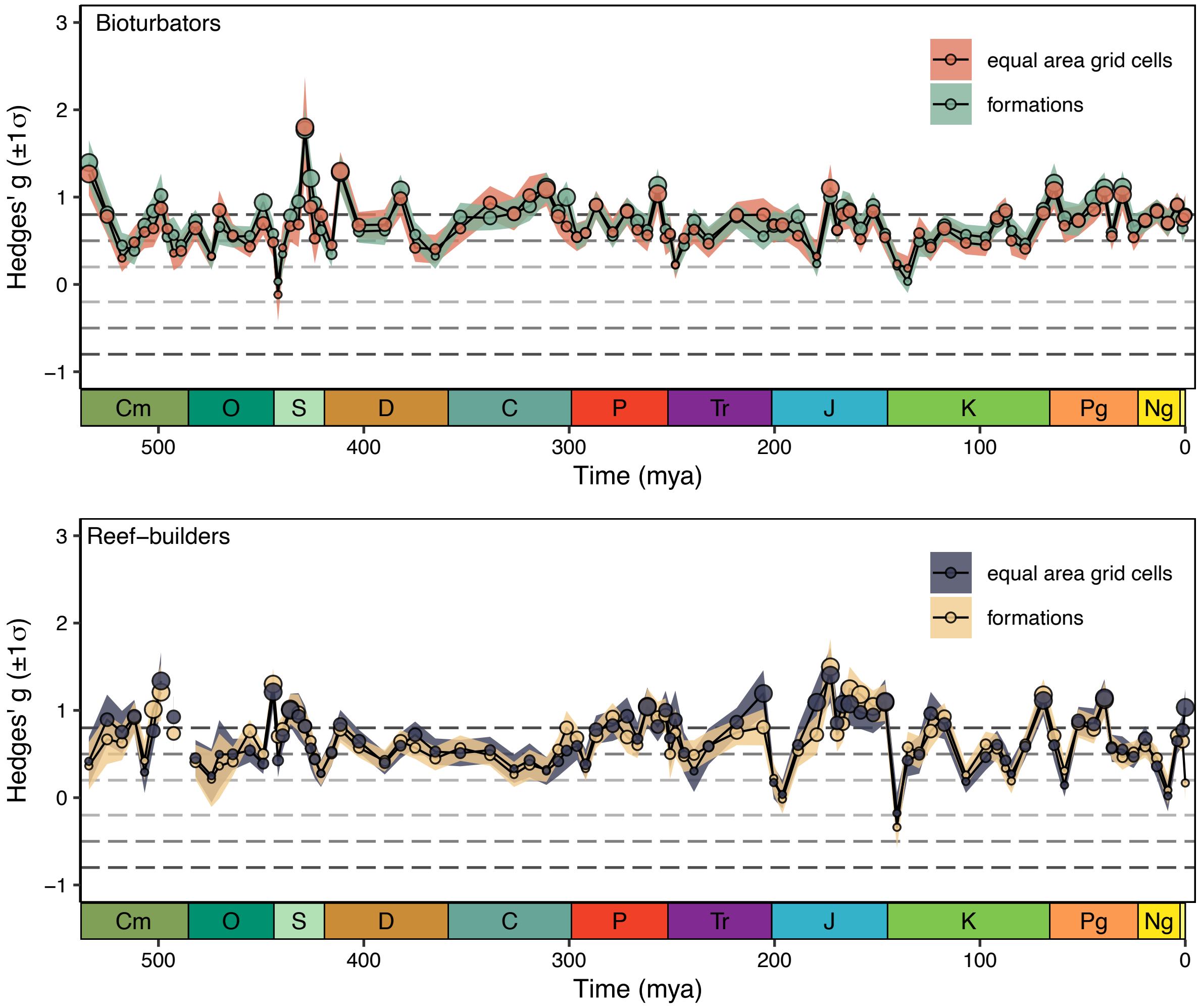

Comparison of effect sizes resulting from equal area grid cell subsampling versus (Supplementary Figures 8,9) within-formation subsampling (main text figures) approaches (see *Methods: subsampling protocol* for details). Points are mean results for 1000 subsampling iterations. Ribbons around points represent uncertainty bounds (±1sd) for 1000 subsampling iterations. We find effect sizes are not impacted by either subsampling approach, as there are no intervals with significant effect size differences in which uncertainty bounds do not overlap. Either subsampling approach is valid and yields the same results, but we choose to use within-formation subsampling due to lower computational costs otherwise associated with spatial R package dependences in the *palaeoverse* (Jones et al., 2023) functions used in the analysis.

Figure S11 | Comparison of threshold test effect size results with full dataset effect size results

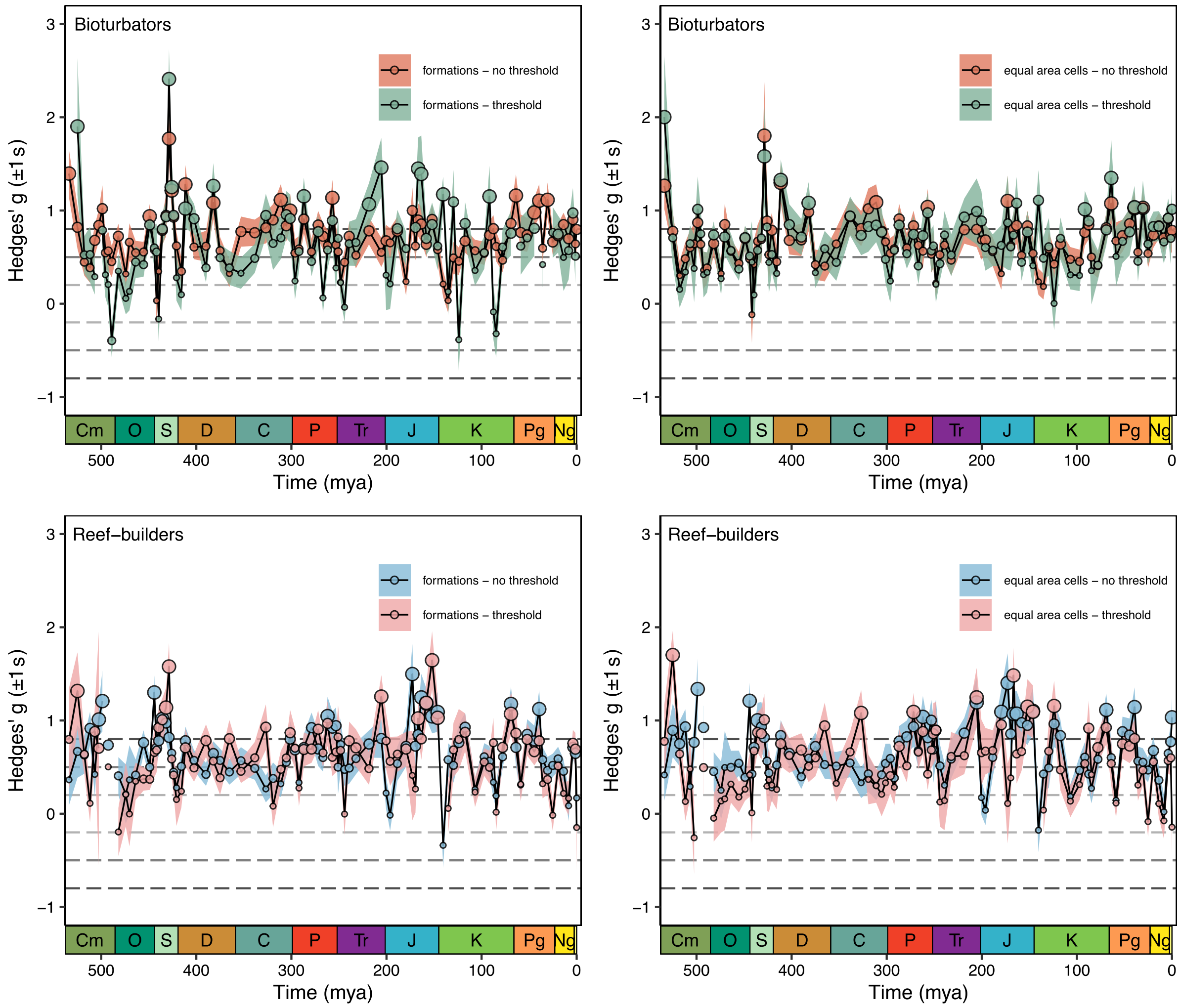

Results of "threshold tests" in which datasets were further processed to remove data from regions that do not meet a certain sampling threshold. For each stage, data were removed from either formations that did not contain at least 20 occurrences or equal-area grid cell regions that did not contain at least 20 occurrences. Analyses were then run as normal on these further processed datasets. While some stage-by-stage differences emerge (for example, there may be a few stronger effect size peaks when comparing across results), the broad Phanerozoic-level conclusions remain. Accounting for under-sampled regions does not mute strong effect sizes. Points are average *g* of 1000 subsampling iterations with shaded color regions representing the error of ±1 standard deviation.

References

1. Kocsis, Á.T., Reddin, C.J., Alroy, J., and Kiessling, W. (2019). The R package divDyn for quantifying diversity dynamics using fossil sampling data. Methods Ecol. Evol. *10*, 735–743. https://doi.org/10.1111/2041-210X.13161

2. Jones, L.A., Gearty, W., Allen, B.J., Eichenseer, K., Dean, C.D., Galván, S., Kouvari, M., Godoy, P.L., Nicholl, C., Dillon, E.M., et al. (2023). palaeoverse: A community-driven R package to support palaeobiological analysis. Methods Ecol. Evol. *14*, 2205–2215. https://doi.org/10.1111/2041-210X.14099.
