# Supplementary material for: Marine biodiversity was greater in the presence of ecosystem engineers throughout the Phanerozoic
Source: Nat Commun. 2026 Jul 28;17:9166. doi: 10.1038/s41467-026-73628-6 (PMC13522536; doi:10.1038/s41467-026-73628-6)
Supplement: Supplementary file 2 [file 41467_2026_73628_MOESM2_ESM.pdf]

nature portfolio

Corresponding author(s): Alison Cribb (A.T.Cribb@soton.ac.uk)

Last updated by author(s): Apr 27, 2026

Reporting Summary

Nature Portfolio wishes to improve the reproducibility of the work that we publish. This form provides structure for consistency and transparency in reporting. For further information on Nature Portfolio policies, see our Editorial Policies and the Editorial Policy Checklist.

Statistics

For all statistical analyses, confirm that the following items are present in the figure legend, table legend, main text, or Methods section.

n/a	Confirmed	
☐	☒	The exact sample size (*n*) for each experimental group/condition, given as a discrete number and unit of measurement
☐	☒	A statement on whether measurements were taken from distinct samples or whether the same sample was measured repeatedly
☐	☒	The statistical test(s) used AND whether they are one- or two-sided<br>*Only common tests should be described solely by name; describe more complex techniques in the Methods section.*
☐	☒	A description of all covariates tested
☐	☒	A description of any assumptions or corrections, such as tests of normality and adjustment for multiple comparisons
☐	☒	A full description of the statistical parameters including central tendency (e.g. means) or other basic estimates (e.g. regression coefficient) AND variation (e.g. standard deviation) or associated estimates of uncertainty (e.g. confidence intervals)
☐	☒	For null hypothesis testing, the test statistic (e.g. *F*, *t*, *r*) with confidence intervals, effect sizes, degrees of freedom and *P* value noted<br>*Give P values as exact values whenever suitable.*
☒	☐	For Bayesian analysis, information on the choice of priors and Markov chain Monte Carlo settings
☒	☐	For hierarchical and complex designs, identification of the appropriate level for tests and full reporting of outcomes
☐	☒	Estimates of effect sizes (e.g. Cohen's *d*, Pearson's *r*), indicating how they were calculated

Our web collection on statistics for biologists contains articles on many of the points above.

Software and code

Policy information about availability of computer code

Data collection	No software was used to collect the source data used in this study.
Data analysis	Analytical code was written in R (most recent version 4.2.2) using the following packages: divDyn (v. 0.8.2), ggplot2 (v. 3.5.1), deeptime (v. 1.0.1), tidyr (v. 1.3.1), wesanderson (v. 0.3.7), fANCOVA (v. 0.6-1), palaeoverse (v 1.4.0), and terra (v 1.8-93). All code is to conduct analyses are recreate figures are the following repository: https://doi.org/10.5281/zenodo.19824423

For manuscripts utilizing custom algorithms or software that are central to the research but not yet described in published literature, software must be made available to editors and reviewers. We strongly encourage code deposition in a community repository (e.g. GitHub). See the Nature Portfolio guidelines for submitting code & software for further information.

Data

Policy information about availability of data

All manuscripts must include a data availability statement. This statement should provide the following information, where applicable:

- Accession codes, unique identifiers, or web links for publicly available datasets
- A description of any restrictions on data availability
- For clinical datasets or third party data, please ensure that the statement adheres to our policy

Source data: Fossil occurrence data was downloaded from the Paleobiology Database (paleobiodb.org) on 1 November 2023. The call for this download was: >Taxonomic resolution: “genus”; >Time:”Cambrian” through “Holocene”; >Geologic context: Environment - “any marine”; > Output options: all. Raw downloaded data was cleaned following description in methodology. Reef-builder data was supplemented with Lee et al. (2019) (doi:10.1016/j.palaeo.2019.02.003) sponge

occurrences, as per reviewer request. Data is available in the .RData files on the Zenodo repository (https://doi.org/10.5281/zenodo.19824423).

Data availability: Source data are provided with this paper. The full Phanerozoic datasets needed to reproduce these analyses are freely available in the GitHub Repository https://github.com/atcribb/Ecosystem-Engineers-Effects and on Zenodo https://doi.org/10.5281/zenodo.19824423. All diversity and effect size results datasets generated in this study have been deposited in these repositories and can be obtained by downloading the relevant files (as outlined by the repository README). Downloading the repository and running each script according to the instructions given in the README will also generate each results dataset.

Code availability: R scripts used in this study are available in the GitHub Repository https://github.com/atcribb/Ecosystem-Engineers-Effects and on Zenodo https://doi.org/10.5281/zenodo.19824423. These R scripts will reproduce each analysis and all figures in the main text and supplementary materials.

Research involving human participants, their data, or biological material

Policy information about studies with human participants or human data. See also policy information about sex, gender (identity/presentation), and sexual orientation and race, ethnicity and racism.

Reporting on sex and gender	n/a
Reporting on race, ethnicity, or other socially relevant groupings	n/a
Population characteristics	n/a
Recruitment	n/a
Ethics oversight	n/a

Note that full information on the approval of the study protocol must also be provided in the manuscript.

Field-specific reporting

Please select the one below that is the best fit for your research. If you are not sure, read the appropriate sections before making your selection.

☐ Life sciences ☐ Behavioural & social sciences ☒ Ecological, evolutionary & environmental sciences

For a reference copy of the document with all sections, see nature.com/documents/nr-reporting-summary-flat.pdf

Ecological, evolutionary & environmental sciences study design

All studies must disclose on these points even when the disclosure is negative.

Study description	We conducted an effect size analysis for the effect of the presence of ecosystem engineers (bioturbating or reef-building taxa) on formation-level biodiversity. For each geologic stage in the Phanerozoic, we computed Hedges' g effect size statistics to quantify the effect of ecosystem engineers on biodiversity. We also tested the predictive strength of the evolution and diversity of ecosystem engineers and palaeoclimate (global average sea surface temperatures) on ecosystem engineer effects on biodiversity. For the former, we hypothesize a linear relationship and calculated linear regression coefficients, R-squared, and F-statistics using the lm() command in R. For predictive strength of palaeoclimate, we hypothesised a non-linear relationship where effect size is strongest at optimum moderate temperatures and weakest at climatic extremes, and testing relationship by computing cross-validated LOESS curves using the loess.as() command from the package fANCOVA.
Research sample	We downloaded marine fossil occurrences from the Cambrian to the Holocene from the Paleobiology Database. Reef-builder data was supplemented with Lee et al. (2019) (doi:10.1016/j.palaeo.2019.02.003) sponge occurrences, as per reviewer request. Data was taxonomically cleaned to remove data that could not be reliably analysed in ecological analyses, included occurrences at taxonomic resolution coarser than genus, problematica, and ichnotaxa. Because ecological metrics were calculated for all fossils within the same formation, any fossil occurrences not assigned a stratigraphic formation were removed during data cleaning. Fossil occurrence formation information was also manually cleaned to resolve formation synonyms, typos, and multiple languages, removing fossils only attributed to formations designated as uncertain entries or lithological descriptions.
Sampling strategy	To minimise temporal sampling biases in the fossil record, we normalised each stratigraphic stage in the effect size analysis to 700 random fossil occurrences, determined by selecting an occurrence quota below the least well-sampled geologic stage. To minimise spatial sampling biases that arise from very well-sampled formations generally having higher biodiversity, we tested three different within-formation subsampling scenarios, and determined that subsampling 20 random fossil occurrences per formation best minimises this spatial bias (determined by selecting the greatest R2 value in computed regressions between formation sampling effort and effect size). For supplementary analyses, following reviewer requests, we also conducted a set of spatial subsampling analyses that binned fossil data into equal-area bins in palaeogeographic space, and normalised 10 fossil occurrences per bin.
Data collection	The PBDB data was downloaded on 1 November 2023 and further processed by Alison Cribb. The paleoclimate data from Scotese et al. (2021) Earth Science Reviews was also downloaded and processed into stage-level time bins by Alison Cribb. Reef-builder data was supplemented with Lee et al. (2019) (doi:10.1016/j.palaeo.2019.02.003) sponge occurrences, as per reviewer request.
Timing and spatial scale	Fossil occurrence data spans from the Cambrian to the Holocene and is stratigraphically binned into geologic stages using the divDyn package in R. Shannon's Diversity (H) values are calculated for every formation in each stage, such that formations represent palaeocommunities, and Hedges' g statistics are calculated for each stage comparing the distribution of H values from formations

where ecosystem engineers are present to the distribution of H values from formations where ecosystem engineers are absent.

Data exclusions	No data fitting the research sample criteria (taxonomic and formation cleaning) were excluded from the main text analyses. Following reviewer requests, supplementary analyses were run as 'threshold' tests in which data from undersampled regions (<20 occurrences per formation or grid cell in each stage) were removed prior to analyses; these are not included in the main text but given as Supplementary Figure 11.
Reproducibility	All data and code are available in a single repository (https://doi.org/10.5281/zenodo.19824423) and on GitHub (https://github.com/atcribb/Ecosystem-Engineers-Effects/releases/tag/v1.2) which can be downloaded by users. This repository contains a README file with instructions to ensure reproducibility. Each analytical script that involved random subsampling has the same set seed used in the original analyses to ensure that the results can be entirely reproduced.
Randomization	To normalise temporal and spatial sampling biases, a standard number of fossil occurrences were randomly subset from each stage and each formation, repeated 1000 times per stage, and final results for each stage were taken as the mean of these 1000 random sampling iterations with ± 1 standard deviation as uncertainty.
Blinding	This research does not evolve experimental design that requires blinding.

Did the study involve field work? ☐ Yes ☒ No

Reporting for specific materials, systems and methods

We require information from authors about some types of materials, experimental systems and methods used in many studies. Here, indicate whether each material, system or method listed is relevant to your study. If you are not sure if a list item applies to your research, read the appropriate section before selecting a response.

Materials & experimental systems

n/a	Involved in the study	
☒	☐	Antibodies
☒	☐	Eukaryotic cell lines
☐	☒	Palaeontology and archaeology
☒	☐	Animals and other organisms
☒	☐	Clinical data
☒	☐	Dual use research of concern
☒	☐	Plants

Methods

n/a	Involved in the study	
☒	☐	ChIP-seq
☒	☐	Flow cytometry
☒	☐	MRI-based neuroimaging

Palaeontology and Archaeology

Specimen provenance	No specimens were studied for this research. Fossil datasets are derived from the open-source Paleobiology Database occurrence dataset. In the raw data file, each fossil occurrence used in this study is tagged with an occurrence number, which can be checked back in the Paleobiology Database to identify provenance.
Specimen deposition	Each fossil occurrence used in this study is tagged with an Paleobiology Database occurrence number, which can be checked back in the Paleobiology Database to identify the original manuscript that notes where specimens are now deposited. Data is freely accessible to all in the Paleobiology Database.
Dating methods	No new dates are provided.

☒ Tick this box to confirm that the raw and calibrated dates are available in the paper or in Supplementary Information.

Ethics oversight	No ethical approval was required for this study, as no fossil specimen were collected from the field or studied in museum collections. Fossil occurrences entered into the Paleobiology Database have been previously published and/or are available in museum collections, and have already been subject to ethical oversight from their respective funding agencies, publishers, and museums.

Note that full information on the approval of the study protocol must also be provided in the manuscript.

Plants

Seed stocks	n/a
Novel plant genotypes	n/a
Authentication	n/a
