# Supplementary material for: Marine biodiversity was greater in the presence of ecosystem engineers throughout the Phanerozoic
Source: Nat Commun. 2026 Jul 28;17:9166. doi: 10.1038/s41467-026-73628-6 (PMC13522536; doi:10.1038/s41467-026-73628-6)
Supplement: Supplementary file 3 [file 41467_2026_73628_MOESM3_ESM.pdf]

nature portfolio

Peer Review File

Marine biodiversity was greater in the presence of ecosystem engineers throughout the Phanerozoic

Corresponding Author: Dr Alison Cribb

This file contains all reviewer reports in order by version, followed by all author rebuttals in order by version.

Attachments originally included by the reviewers as part of their assessment can be found at the end of this file.

Version 0:

Reviewer comments:

Reviewer #1

(Remarks to the Author)
See attached.

(Remarks on code availability)
The study appears to be fully reproducible.

Reviewer #2

(Remarks to the Author)
This manuscript examines the effects of ecosystem engineers on marine biodiversity throughout the Phanerozoic. In particular, it emphasizes two primary types of ecosystem engineers that function on different levels: bioturbators in the sediment and reef builders above it in marine environments. The results show that both groups of ecosystem engineers have a positive effect on the Phanerozoic biodiversity.

The manuscript is well-written and easy to follow. The hypotheses are clearly stated. In the results and discussion, the authors comprehensively cover various aspects of biodiversity change associated with ecosystem engineers, including mass extinction events, climatic changes, and evolutionary trends.

The methods are detailed, with explanations and rationales for each statistical test, which ensures reproducibility. Various analytical approaches were implemented to address spatial and temporal sampling biases, and different sensitivity tests were employed to help mitigate these biases.

Overall, this manuscript significantly contributes to understanding the role of ecosystem engineers in marine biodiversity throughout the Phanerozoic.

General suggestions for improvement:

Lines 16-19: It sounds here that both groups of ecosystem engineers were analyzed together concerning their impact on the overall biodiversity. It would be helpful to clarify that the analyses were conducted separately for each group.

Lines 81-84: Consider citing more recent publication on reef refugia by Dimitrijevic et al. (2024) https://link.springer.com/article/10.1007/s00338-024-02548-y#citeas

Lines 211-213: Similar to the abstract, this sentence sounds like you have analyzed both groups together. Speaking of which, what would the patterns be if both ecosystem engineer groups were analyzed together?

Lines 248-249: Why “even” sponges when they play the same functional role in the ecosystem by providing a three-dimensional habitat? Also, did you, by any chance, compare the effect of corals only vs. sponges? It would be interesting to see the patterns of individual reef-building groups.

Lines 252-256: I do not understand the point you are trying to convey here regarding the reef builders. There were other reef builders before the scleractinian corals appeared in the fossil record, and Paleozoic corals also potentially hosted photosymbionts, see for example, Jung et al. (2024) https://www.nature.com/articles/s41586-024-08101-9
And Zapalski et al. (2017) https://www.sciencedirect.com/science/article/pii/S0031018217306855?via=ihub

Lines 257-258: why are sponges considered as “weaker” ecosystem engineers? They built prolific reefs in the Paleozoic and through the Early Triassic (see Kiessling 2009 https://www.annualreviews.org/content/journals/10.1146/annurev.ecolsys.110308.120251 and references therein).

Line 293-295: I wouldn’t say this is surprising at all. Sponge reefs persisted well into the Early Triassic (i.e., “Permian heritage”, See Flügel et al. 2002 Triassic reef patterns and Weidlich et al. 2003 https://pubs.geoscienceworld.org/gsa/geology/article-abstract/31/11/961/29201/Permian-Triassic-boundary-interval-as-a-model-for?redirectedFrom=fulltext) so that the functional role of reef builders was not compromised despite the coral reef gap. So there is, basically, the opposite situation from the one described in the previous lines of the discussion at the end-Devonian when the coral reefs functionally replaced stromatoporoid reefs.

Line 299: I suggest citing other references here and elsewhere apart from one own. For example, Kiessling, Flügel, etc.

Line 303-305: Or this signal is visible because they are the dominant reef builders at this time while other periods have multiple reef builders that mask the signal by replacing the functional roles. It would be great to test this hypothesis by analyzing the effects of individual reef-building groups.

Lines 409-412: I don’t quite understand the reasoning for selecting these 10 particular reef-building groups, and why do you consider some as more important than others, for example, you selected encrusting tube worms, but why not bryozoans or algae? An explanation of the reasoning why some reef-building organisms are excluded would be appreciated.

Also, not all reef-related organisms contribute to reef building, such as solitary scleractinian and solitary rugose corals. Even considering the “true” reef builders (i.e., colonial corals), their occurrences can often be part of the bottom-level communities and not build reefs. It would be great to highlight this in the discussion as part of the potential biases of the study.

Lines 412-413: Please be careful with terminology; you did not analyze reefs but rather reef builder occurrences. As I mentioned above, reef-building organisms do not necessarily build reefs, and it should be clarified that reef-building occurrences are not synonymous with reefs.

Lines 423-425: Please clarify why the standardized subsampling number was set to 700.

Lines 465-467: It’s unclear if the dataset with bioturbators includes reef builders if the pool without bioturbators has reef builders, and vice versa. I recommend rephrasing this section for clarity and labeling the datasets with small Roman numerals or letters.

Figures:
Please explain the error bars in the captions.
Supplementary Figure 1: are the shaded areas confidence intervals? Please state so in the figure caption.
Supplementary Figures 2 and 4 are hard to read; you could try with proportional polygon plots.

Minor comments:

- Supplementary Table 4 is marked as 3
- Line 83: should be environmental stress
- Line 455: Lagerstätten is misspelled (i.e., ä is in the wrong place), and the word's first letter should be capitalized.
- Line 701: there is a point missing at 0.8 in the figure caption

(Remarks on code availability)

Reviewer #3

(Remarks to the Author)
Reviewer’s report on manuscript by Cribb et al., entitled: “Marine Phanerozoic biodiversity increased in presence of ecosystem engineers”.
1) General comment: This is the interesting paper, which has analyzed fossil communities with and without ecosystem engineers to calculate the impact of the presence of ecosystem engineers throughout the Phanerozoic. Although ecosystem engineers were thought to have affected ancient ecosystems and biodiversity, quantitative estimation had not yet been explored. The author’s study indicates that ecosystem engineers are significantly associated with increased biodiversity base on Paleobiology Database.

2) Lines 46-49: “Moreover, as recent advances have underscored the importance of local- and regional-scale biotic changes in driving global Phanerozoic biodiversity trends15,16, there is a need to understand the importance and impacts of these key biotic interactions.”
Comment: Although the authors state that local- and regional-scale biotic changes impact biodiversity, this sentence is unnecessary because the paper does not address local- and regional-scale biotic changes.
3) Lines 106-110: “There are notable effect size peaks in the Katian during the Ordovician (g=0.94±0.13) in the Homerian (g=1.77±0.27) during the Silurian, in the Pragian (g=1.28±0.21) and Givetian (g=1.08±0.12) during the Devonian, in the Wuchiapingian during the Permian (g=1.14±0.19), and in the Danian (g=1.16±0.23) during the Paleogene. While there are significantly strong positive effect sizes…”.
Comment: What are the notable effect sizes and significantly strong positive effect sizes? Why are the Cambrian Guzhangian (g = 1.02 ± 0.25) and Jurassic Aalenian (g = 1.00 ± 0.25) not included here"
4) Lines 138-141” Effect sizes rapidly decline to insignificant or even weakly negative values most notably during the early Jurassic (Hettangian g=0.22±0.17; Sinemurian g=-0.02±0.17, the Berriasian (g=-0.34±0.23), the Albian (g=0.26±0.12), and the Santonian (g=0.19±0.13) (Supplementary Table 4).”
Comment: Isn’t the Upper Miocene effect size (g = 0.09 ± 0.16) included here?
5) Lines 192-195” While we do not find a clear, robust relationship between temperature and effect sizes, the strongest effects of bioturbators do occur in moderate global temperatures in the range ~16-22°C, and effect sizes decrease as the climate warms or cools towards extremes (Figure 6B).”
Comment: The authors state that 'they did not find a clear relationship between temperature and effect size.' However, in lines 25-26, the authors mention that ecosystem engineers impact climate. There is no clear impact, but climate modulates ecosystems. What does this mean?
6) Lines 238-240: “For example, the Homerian effect size peak is most likely driven by the invasion of deposit feeding ecosystem engineer.”
Comment: Precisely, which groups contributed as deposit feeding ecosystem engineer?
7) Lines 247-249: “We find this is not limited to coral ecosystem engineers; even sponge reef ecosystems apparently had significant impacts on biodiversity.”
Comment: Does this simply mean that stromatoporoid sponges dominating Devonian reefs also indicate a positive effect on biodiversity? Devonian stromatoporoid reefs include tabulate corals as well. Could these reef ecosystems be called sponge reef ecosystems?
8) Lines 249-251: “Our results illustrate positive effect sizes during intervals of the Cambrian when reefs are made entirely of hexactinellid sponges, and also during the Hirnantian, …”
Comment: The hexactinellid sponge-bearing Cambrian reefs included dominant microbial components. Did the hexactinellid sponges primarily affect biodiversity? The transition from microbial-dominated to metazoan-dominated reefs occurred in the Ordovician. To what extent did the increase in ecosystem engineers, such as metazoan reef builders, impact biodiversity during this interval?
9) Lines 268-270: “For example, much of the weak and insignificant effect sizes during the Cretaceous are associated with the rise of rudist bivalve reefs39, which were apparently less efficient ecosystem engineers in terms of driving increased biodiversity (Figure 2). “
Comment: The rudist bivalves are relatively simple and they do not create “true reefs” in the same way that corals and sponges did.
10) Lines 293-300: “The persistence of reef effects through the end-Permian mass extinction is particularly surprising given its associated coral reef collapse44. Reef-associated global diversity certainly significantly declined during this interval. However, our formation-level results indicate this would have been primarily due to the loss of the number of reef ecosystems (Supplementary Table 3), rather than the inhibition of surviving reef-builders’ ecosystem engineering strength, as other non-coral reef-builder survivors in the early Triassic39 maintained communities where biodiversity was still, on average, higher than in non-reef communities (Figure 2A).”
Comment: What does the inhibition of surviving reef-builders’ ecosystem engineering strength means? Very a few sponges are known from the microbial reefs during the early Triassic. Did these rare sponges contribute as ecosystem engineers?
11) “Hedges’ g values are not possible for the Paibian, where only one formation contains reef-building ecosystem engineers (so s cannot be measured), and for Stage 10, when a metazoan reef gap occurred. “
Comment: Is the metazoan reef gap is Stage 10 real? Lithistid sponge-bearing microbial reefs are known to exist.
12) Supplementary Table 3, “n1 =6 of the Cambrian Fortunian”.
Comment: Which metazoans are identified as metazoan reef builders? Archaeocyaths were not present in the Cambrian Fortunian.
13) Supplementary Table 3”
Comment: What types of reef-builders were identified in n1=12 and 10 of the Triassic Induan and Olenekian? Was a metazoan reef gap was previously thought to exist in these intervals?

1) L205; “moderate global climate between ~20-23°C (Figure 5D). “ (Figure 5D) should be changed to (Figure 6D).
2) Supplementary Figure 4” Palaeoenvironments classed as siliciclastic (purples) or carbonates (pinks), and shallow depths (medium), deep depths (dark), or unclassified depths (light).
3) Supplementary Table 1, “HBA = mean Shannon’s Diversity across formations where bioturbators are present;” HBA revise into HBP.
4) Supplementary Table 3 , “Comparison of Shannon’s Diversity (H) in formations with (HRA) and without (HRP) reef-builders” HRA and HRP revise into HRP and HRA
5) Supplementary Table 3. “Results of effect size analysis for the presence of reef-builders. “Supplementary Table 3 revise into Supplementary Table 4

(Remarks on code availability)

Reviewer #4

(Remarks to the Author)
This manuscript presents a compelling investigation into the role of bioturbating and reef-building ecosystem engineers on marine biodiversity across the Phanerozoic. The analysis leverages fossil occurrence data and modern ecological methods to provide a quantitative assessment of the biodiversity impacts of ecosystem engineers over geologic time. The study's strengths include its novel focus, rigorous statistical treatment, and integration of paleoecological and climatic perspectives. Additionally, the authors provide a well-rounded discussion, highlighting the limitations of their analysis while emphasizing key findings: (1) Consistent and significant positive relationship between the presence of bioturbating and reef-building ecosystem engineers and biodiversity across most of the Phanerozoic. (2) The two groups of ecosystem engineers responded differently to mass extinction events, with their effects on biodiversity varying depending on evolutionary context and ecological conditions. (3) While the diversification of these groups does not appear to directly explain the observed trends, the authors suggest that climate likely played a role in modulating their influence on biodiversity, particularly under conditions of moderate global temperatures. This thorough consideration of both the strengths and constraints of the analysis enhances the robustness of the study's conclusions.
The authors show methodological rigor: i.e. ubsampling protocols and effect size analyses are well-detailed, ensuring transparency and reproducibility. Moreover, the the results emphasize the fundamental role of biotic interactions in shaping marine biodiversity and provide insights into how ecosystem engineering influences biodiversity under varying climatic conditions. Moreover, the figures effectively display trends and results across the Phanerozoic, though some refinements could enhance their interpretability (see suggestions below). Thus, I believe the authors should be proud of their detailed analyses and robust discussion. The integration of paleoclimate and biodiversity dynamics provides a comprehensive narrative that will resonate with both paleoecologists and macroecologists. Addressing these minor suggestions will further strengthen the clarity and accessibility of this excellent study.

Suggestions for Minor Changes:
Adding Age/Time to Geological Periods:
Include the approximate age range of each geological period mentioned throughout the text (e.g., "Cambrian (~541–485 Ma)") to assist readers unfamiliar with the names in geologic time scales.
Figures 1 and 2 could be merged. Combining Figures 1 and 2 into a single figure with consistent formatting for both bioturbators and reef-builders would streamline comparisons. Hedges' g Plot could be simplified, i.e. standardize the size of data points and use gradients or horizontal lines to emphasize effect size trends, shown per see on the Hedges's g value on the y axis. This adjustment would maintain clarity while reducing visual clutter.
Figure 4 (Global Generic Richness):
Clarify whether "global generic richness" is measured or directly observed. If derived from models, make this explicit.
Confirm whether the term "generic richness" consistently refers to "genera richness" or species-level data. A consistent term will avoid confusion.
Figure 6 (Bioturbators vs. Reef Builders):
Add a clear title indicating the figure's focus on comparing bioturbators and reef builders, including a brief note about their respective impacts.

(Remarks on code availability)
The code provided by the authors is clear, well-structured, and easy to follow. Furthermore, all necessary materials for reproducibility, including data, scripts, and detailed instructions, are readily available

Version 1:

Reviewer comments:

Reviewer #1

(Remarks to the Author)
I would like to thank the authors for engaging with my comments so thoroughly and constructively, and apologise for the delay in my review of the revised version of this manuscript. All issues I initially raised have been satisfactorily addressed through editing, additional analyses, or thoughtful responses. Whilst I cannot speak for the author's responses to the other reviewer's comments (as I have not reviewed them in the interest of expediting the return of my review), from my point of view, the manuscript is ready for publication. I recommend it for acceptance.

If the other reviewer is not satisfied with the author's responses and so an additional round of revisions becomes necessary, then I think a great addition to this manuscript to bulletproof the results would be additional sensitivity tests where occurrences are rasterised and grid cells that don't meet some quality criteria (e.g., minimum number of occurrences, minimum number of genera, etc.) are filtered out before the 700 occurrences are drawn from each stage as part of the normal pipeline. Something similar for the formation analyses would be reassuring as well (i.e., the application of quality criteria to the formations before the dataset is analysed). HOWEVER, I stress these sensitivity tests are not essential. Just something I think would be worth checking if there are other issues that necessitate a second round of revisions.

Congratulations to the authors for a job well done!

Best wishes,
Tom Roper-Smith
Museum of Natural History
University of Oxford

(Remarks on code availability)
Based upon my assessment of the code, the results of this study are reproducible. The README file is informative and the code properly annotated.

Reviewer #2

(Remarks to the Author)
The authors have addressed the comments appropriately, and I believe this manuscript is ready for publication in its current form.

(Remarks on code availability)
All good.

Reviewer #3

(Remarks to the Author)
I have carefully reviewed the revised manuscript and the authors’ responses to the reviewers’ comments. The manuscript has been revised in accordance with most of the reviewers’ suggestions. I also agree with the authors’ decisions regarding the points where they did not fully follow the reviewers’ comments.

(Remarks on code availability)

Open Access This Peer Review File is licensed under a Creative Commons Attribution 4.0 International License, which permits use, sharing, adaptation, distribution and reproduction in any medium or format, as long as you give appropriate credit to the original author(s) and the source, provide a link to the Creative Commons license, and indicate if changes were made.
In cases where reviewers are anonymous, credit should be given to 'Anonymous Referee' and the source.
The images or other third party material in this Peer Review File are included in the article’s Creative Commons license, unless indicated otherwise in a credit line to the material. If material is not included in the article’s Creative Commons license and your intended use is not permitted by statutory regulation or exceeds the permitted use, you will need to obtain permission directly from the copyright holder.

To view a copy of this license, visit https://creativecommons.org/licenses/by/4.0/

Reviewer 1

Thank you for inviting me to review this manuscript and please accept my apologies for my delayed response. If you have any questions about any of my comments, I will endeavour to reply right away to speed up the rest of the review process.
This paper by Cribb and colleagues describes the results of an exciting new set of analyses using a refined version of the PBDB (the standard for such analyses) which demonstrate that ecosystem engineers, specifically reef-builders and bioturbators, have a significant impact on the taxonomic diversity of the geological formations they originate from across the Phanerozoic. As far as I am aware, the results of such ambitious analyses have never been published before, which would make this paper a worthy addition to the *Nature Communications* Portfolio. I would like to congratulate the authors for their excellent writing, clear figures, and overall great work.

We thank you sincerely for reviewing the manuscript and providing such helpful and constructive feedback. Following revisions made in response to your comments, we believe that the manuscript is significantly improved. Please find our detailed responses below.

I only have two issues with the paper as currently written that require addressing before I can recommend it for acceptance for publication (the rest of my comments below are very minor). These are outlined in detail below but in summary they are:

1. Whether the within-formation subsampling the authors use mitigates the spatial biases in the PBDB as well as the more common practice of geographic grid cell subsampling,

Changes made: Thank you for this suggestion. We provide more detail below, but in summary, we have no conducted two different subsampling procedures in the manuscript. The first is our original procedure, which subsamples at the formation level to account for collection efforts. The second follows your recommendation, in which we bin each fossil occurrence at each time stage into equal area bins and normalize sampling such that each equal area bin contains the same number of fossils. Comparing these two procedures, we find insignificant differences – likely arising because the majority of our formations are not geographically widespread. We have opted to keep the former within-formation subsampling procedure in the main text analyses, as we think this better reflects and more directly addresses a critical bias which would impact our results, which is that greater sampling in a geologic unit (and, for example, in very ‘famous’ geologic units such as the Wenlock Formation in the UK) leads would lead to increased biodiversity, rather than an effect of ecosystem engineers. The equal area bin subsampling results are provided as well in the supplemental.

2. Whether the variation in palaeoenvironment composition through time between the formations that have ecosystem engineers and those that don't is having an impact on effect size.
Changes made: Thank you for this comment! We go into further detail below but to sum: we agree with your comments, although we emphasize that variations in the effect sizes when they are isolated in certain palaeoenvironments does not detract from the overall message, which is that positive effect sizes are persistent throughout the Phanerozoic. Further work exploring *why* some palaeoenvironments cause smaller or larger positive effect sizes will be a promising avenue, and we are appreciative of the ideas! We have clarified our text in a number of places throughout the manuscript to clarify our interpretations about the palaeoenvironments. Further details are in our comments below.

If the authors have any questions about how best to approach this, I am happy for them to get in touch with me directly, as I think this will be a very impactful study when published.
Best wishes,
Tom Smith
Department of Earth Sciences
University of Oxford

Specific comments:
Introduction:
Line 36: I don't think "Diversity has been generally increasing" encompasses the full spectrum of recent results. Close et al's (2020*, Science*, reference 15) spatially standardised analyses recover a noisy pattern with comparable spikes throughout the Phanerozoic. Given the rest of the paragraph focusses on the first key feature (that diversity rises and falls), maybe tweak this sentence to reflect that perceptions of diversity depend on how data are standardised or something else?
Changes made: Thank you for the suggestion – we agree this text originally did not fully reflect how broadest observations of marine diversity have been scrutinised and re-examined in light of various biases, including those investigated by Close et al. (2020) and effects such as the "Pull of the Recent"and stratigraphic completeness. We have revise this text to now read:

"With global datasets such as the Paleobiology Database and new statistical treatments of the fossil record, two broad key observations of the marine Phanerozoic biodiversity record have emerged: diversity over time has been subject to numerous rises and falls, and diversity appears to have been increasing. **Since early endeavours to document the history of marine biodiversity (Sepkoski et al. 1981), these observations have been subject to a long history of scrutiny, including the demonstration of the impacts of spatial sampling biases (Close et al. 2020), the effects of changing stratigraphic completeness (Sadler 1981), and the extent to which the observed increase in biodiversity is an artifact of the "Pull of the Recent" (Jablonski et al. 2003; Womack et al 2020). Taking the scrutinized**

reconstructions of ancient biodiversity dynamics as true biological signals, much of this dynamism has been attributed to interactions between life and the abiotic environment, such as biosphere responses to changes in global temperatures, oxygen levels, continental configuration, and sea level. Climate change, for example, is strongly linked to macroevolutionary dynamics, most dramatically during mass extinctions and their subsequent recovery intervals."

Lines 50-53: A bit of a pedantic point (sorry!) but I think it is important to explicitly state that ecosystem engineers differentiate themselves from other organisms in that they change the resource availability for other organisms, not just themselves. Maybe just add 'for other organisms' to the end of the sentence?
Changes made: Thank you for the suggestion. Definitely not a pedantic point, and we agree! We have edited this text to make your suggested clarification.

Line 64: typo? "key drivers of increasing biodiversity"?
Changes made: Thank you for pointing this out – we have edited the text accordingly.

Results – effects on Phanerozoic biodiversity:
Lines 93-95: reference to Figures 1 and 2 needed.
Changes made: Thank you for the comment. We have added references to Figures 1 and 2 where appropriate.

95-96: I think it could be worth defining weak, significant, and strong effect sizes up top. Perhaps "These associations are reflected in strong (g≥0.8 with non-overlapping uncertainty, significant (g≥0.5), and weak (g≥0.2) positive effect sizes? I know this is in the Methods of the paper but I think having it here will help.
Changes made: Thank you for the suggestion! We agree this clarification at the top of the results helps with interpretation. We think this is best introduced just before the effect sizes are directly discussed in the manuscript, so we have added the following text:

"These significantly positive effect sizes **(with non-overlapping uncertainty bounds, strong = $g \geq 0.8$; moderate = $g \geq 0.5$; weak = $g \geq 0.2$)** arise early in the Cambrian...."
Results: Effects on Phanerozoic biodiversity, Lines 117-118

Lines 96-99: I appreciate the author's candour here and this is a completely subjective opinion (so please feel free to ignore) but I feel qualifying statements (especially those referring the supplementary figures) are best saved for the end of the sections they pertain to. I'd suggest moving these lines to the end of the subsection, so readers have a better idea as to what they pertain to.
Changes not made: Thank you for the comment and suggestion. We understand your point, although we kindly disagree, preferring to address these points and qualify results in the face of two of the most obvious caveats head on.

Lines 102-105: Similar to above, I feel pointing readers to supplemental figures showing the impact of palaeoenvironment before digging into the main data figures is a bit confusing. I'd recommend moving this aside to the end of the paragraph.
Changes not made: Thank you for the comment, but please see our response above.

Line 114: maybe quantify the difference between effect sizes required between a pair of stages for the latter to be considered notable?
Changes made: Thank you for the comment. Originally, this was clarified in the sentence following noting these peaks, but we see this would be more clear if we introduced the qualification first. We have therefore swapped these sentences around, and the text now reads as follows:
"**While there are a significantly strong positive effect sizes in numerous stages throughout the Phanerozoic (Figure 1B; Supplementary Table 2), there are several notable effect size peaks in which diversity in formations with bioturbators becomes**

much greater than formations without bioturbators, such that effect sizes are strong ($g \geq 0.8$) and significantly different to the previous stage (non-overlapping uncertainty). These effect size peaks occur in the Katian (~453-445 Ma) (g=0.94±0.13) during the Ordovician, in the Homerian (~431-427 Ma) (g=1.77±0.27) during the Silurian, in the Pragian (~413-410 Ma) (g=1.28±0.21) and Givetian (~388-382 Ma) (g=1.08±0.12) during the Devonian, in the Wuchiapingian (~259-254 Ma) during the Permian (g=1.14±0.19), and in the Danian (~66-62 Ma) (g=1.16±0.23) during the Paleogene." *Results: Effects on Phanerozoic biodiversity, Lines 110-120*

Line 125: reference to Figures 2 needed.
Changes made: Thank you for pointing this out. Reference to supplemental tables of effect sizes is also needed here, so have updated the text accordingly.

Lins 129-131: similar to the paragraph about bioturbators, I'd move the nod to the supplemental results looking at the impact of palaeoenvironment to the end of the paragraph.
Changes made: Thank you for the suggestion. Here, we agree with your points, as we had already introduced this caveat early, and so we have moved this text to the end of the paragraph.

Results – effects during mass extinctions:
Lines 149-150: I think the description of the effect sizes of bioturbators across the end-Ordovician mass extinction needs changing to reflect the crash before the boundary and steady increase across it.
Changes made: Thank you for the suggestion – we agree this would be a clearer presentation and discussion of the results. We have revised the text to the following: "In contrast, effect sizes for the presence of bioturbators decline to insignificant effects early, declining to insignificant effects between the Hirnantian and Rhuddanian, although increasing back to significantly positive effects by the Telychian (Figure 3A)."

Line 164: "significant moderately positive" is a bit confusing. Significant positive is enough if you explicitly specify what each effect size category is comprised of at the beginning of the Results.
Changes made: Thank you for the suggestion. As we have made the suggested changes to the start of the Results to introduce the significance thresholds, we have revised this text to just say "significantly positive."

General: I wonder if there is a way this section can be re-written to make it less repetitive and more concise. This is just a suggestion, so please feel free to ignore,

but I think these results would be much easier to digest if you discussed bioturbators and reef-builders separately and didn't describe each curve so granularly (the figures are clear enough to make such descriptions unnecessary in my opinion). For each, you could state over which boundaries each of them increases/decreases/stays the same and then wrap up with the summary.

Changes made: We agree and thank the reviewer for the prompt. We have substantially shortened the Results section – in particular the granular description of the curves – while adding summary text at the top of each sub-heading that hopefully conveys the most important points. We have also shortened and condensed parts of the Results that deal with extinction events and correlations with biodiversity/climate; we considered amalgamating these with the previous sub-section, but ultimately, we feel that structuring our results to clearly align with our 3 original hypotheses was the most intuitive way of organizing the text.

Discussion:

General: I think the discussion is well-written and doesn't overreach. However, I do think some space needs to be given to a discussion about the interaction between time, environment (shallow versus deep, carbonate versus siliciclastic), and effect size. Looking at supplementary figures 3 and 5 (which I feel belong in the main text, personally), formations with bioturbators and without reef-builders seem to be split 50-50 between siliciclastic and carbonate sediments in more stages than those with reef builders and without bioturbators (which seen to lean carbonate).

This strikes me as something that could impact the analyses and needs addressing in some way. Below I suggest fitting a GLMM to the data but I am open to other solutions.

Line 246: "reef-builder" missing 's'.
Changes made: Thank you for pointing this out. We have fixed this text.

Line 343: "more particularly" maybe drop the more?
Changes made: Thank you – we agree and have removed "more" from this sentence.

Methods – subsampling protocol:
General: I think I understood what the authors did here (first sampling 700 fossil occurrences for each stage, then from all the formations represented within this 700-occurrence sample, sampling 20 occurrences of each) but it's unclear. Was the random sampling of the formation occurrences conducted with replacement within each time bin? A bit of extra detail would be great.
Changes made: Thank you for the question. We are happy to clarify this text. To answer your question – yes, we opt for rarefaction at both steps and replace within bin and within formations. After describing how we selected our subsampling procedures, we added the follow text to clarify (particularly keeping in mind readers with less of a detailed knowledge of subsampling procedures):

"Ultimately, our optimised subsampling protocol takes 700 random fossil occurrences per stage, and from the formations represented in those subsampled occurrences, we take a further random 20 occurrences per formation." *Lines 481-484*

Within-formation subsampling: While I think the authors protocol is effective in terms of standardising the number of occurrences per formation, I am unsure as to how effective it is at controlling for the impact of the species-area effect, given that geological formations vary in the size of the geographic areas they preserve. Therefore, I am a little concerned that there may still be spatial biases lurking in the results. This is easily addressed, however! The authors could add of a rasterization step into the subsampling pipeline after time-binning the data but before standardising the number of occurrences per formation. This would necessitate the dropping of the stage-level subsampling but, given that the number of occurrences will be standardised by later steps in the pipeline, I don't think this will be an issue. Alternatively, they could run a sensitivity test that swaps the within-formation subsampling for a within-grid-cell subsampling alternative (again, it would likely become necessary to drop the stage subsampling step) and then compares the results of the two for differences. Either way, I'd like to see this tested.

Changes made:

Thank you for the suggestion – we agree that this is an important bias to mitigate, so we have included spatial subsampling. More in line with the latter half of your suggestion, we apply the following approach: in each stage, we 1) subsample 700 occurrences, 2) grid our data into equal-area grid cells, and 3) using their

palaeocoordinates (Palaeoverse::palaeorotate, PALEOMAP model), 10 collections per grid cell are subsampled (here, not allowing for repeat collection subsampling). We find these parameters best minimised uncertainty and generally follow recent approaches (e.g., Close et al.). From this subsampled data, we conduct our analyses to calculate Shannon's Diversity in formations with and without ecosystem engineers and then conduct Hedges' g. We repeat this 1000 times per stage to obtain the final mean±1 standard deviation:

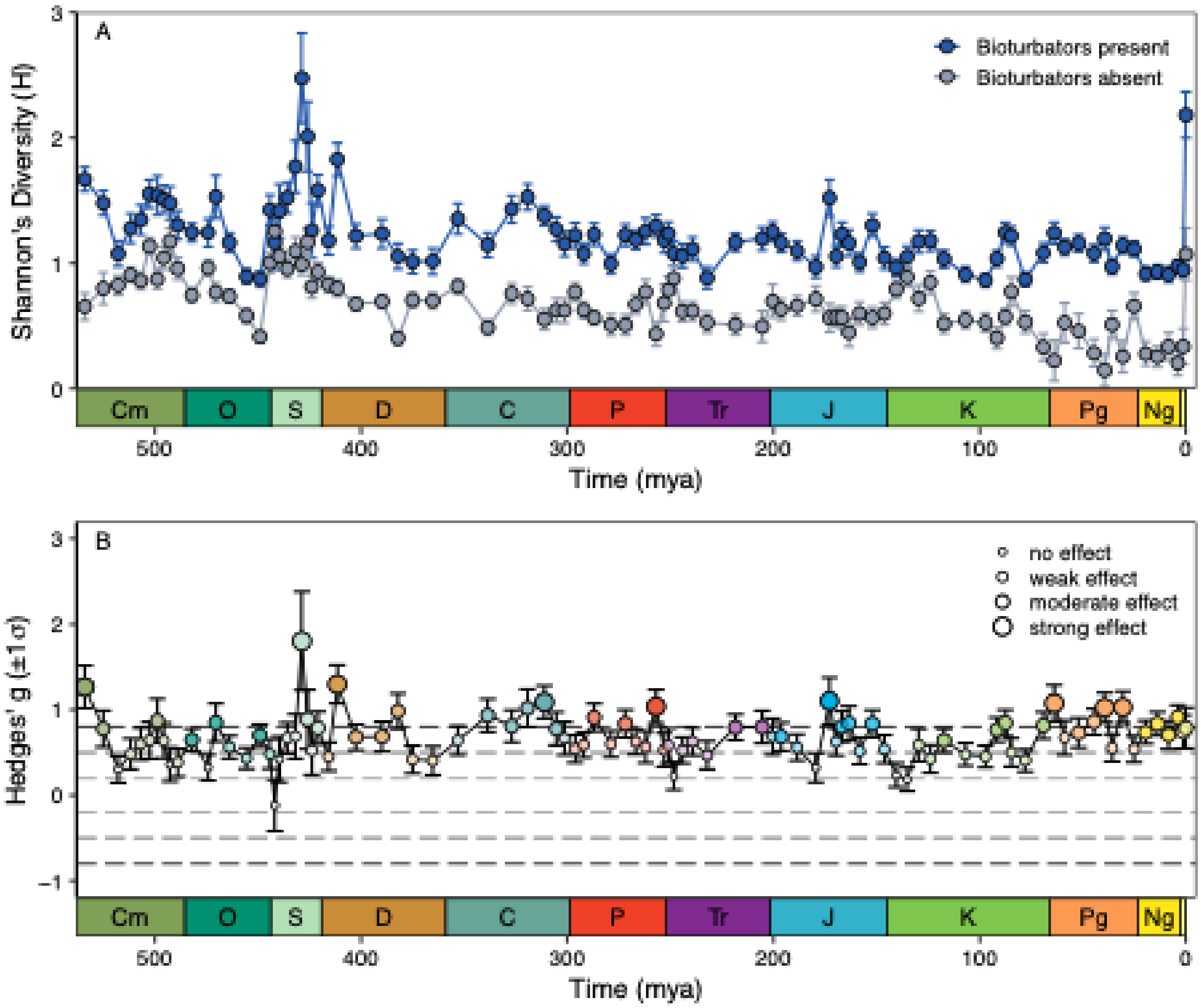

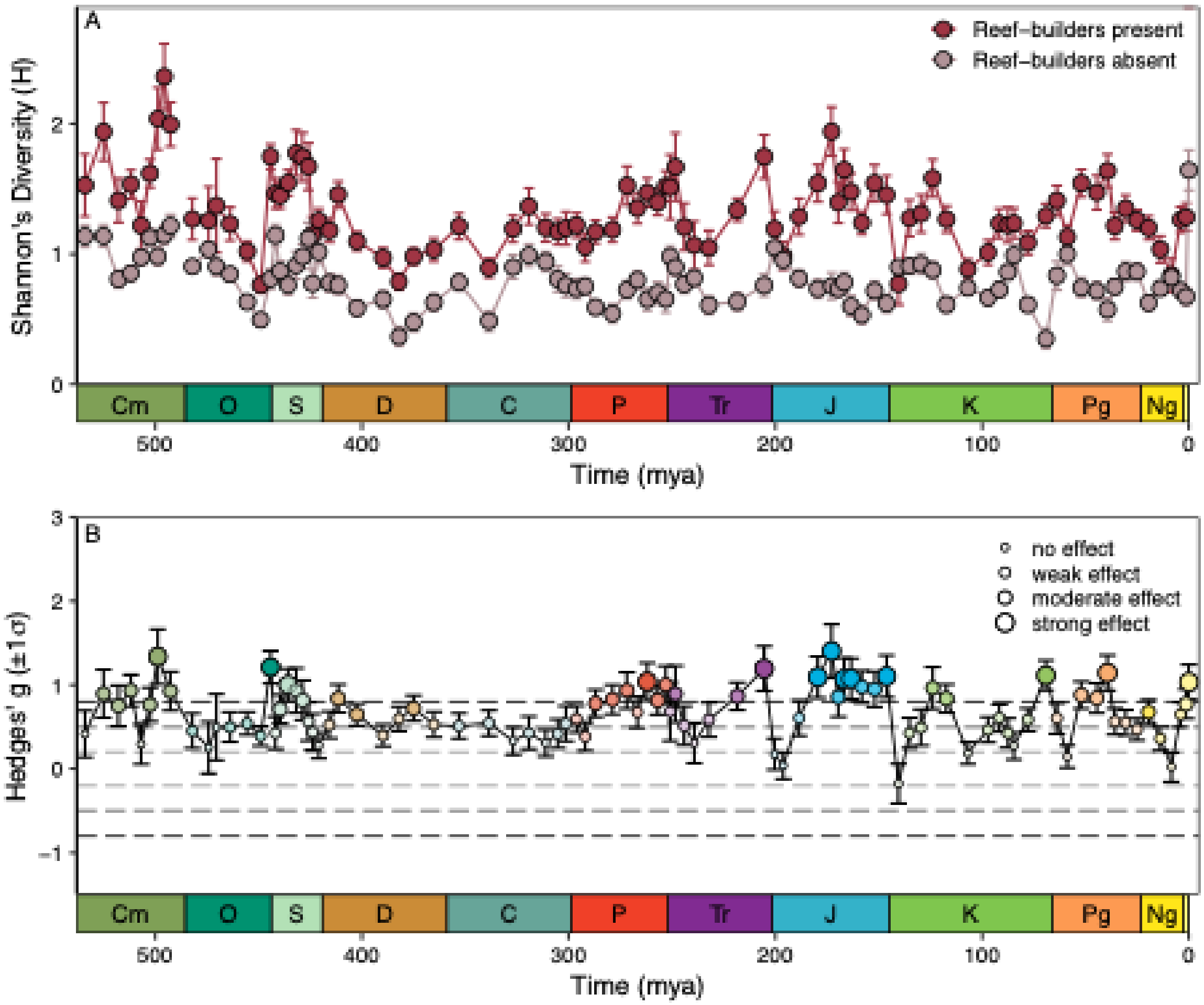

We find insignificant differences between these the within-grid cell and within-formation subsampling protocols see comparison figures below), most likely because these formations (or at least, the data within them) fortunately do not have very large geographic ranges. We therefore have opted to keep the within-formation subsampling as our main text figures and have added the within-equal area grid cell subsampling to the supplemental

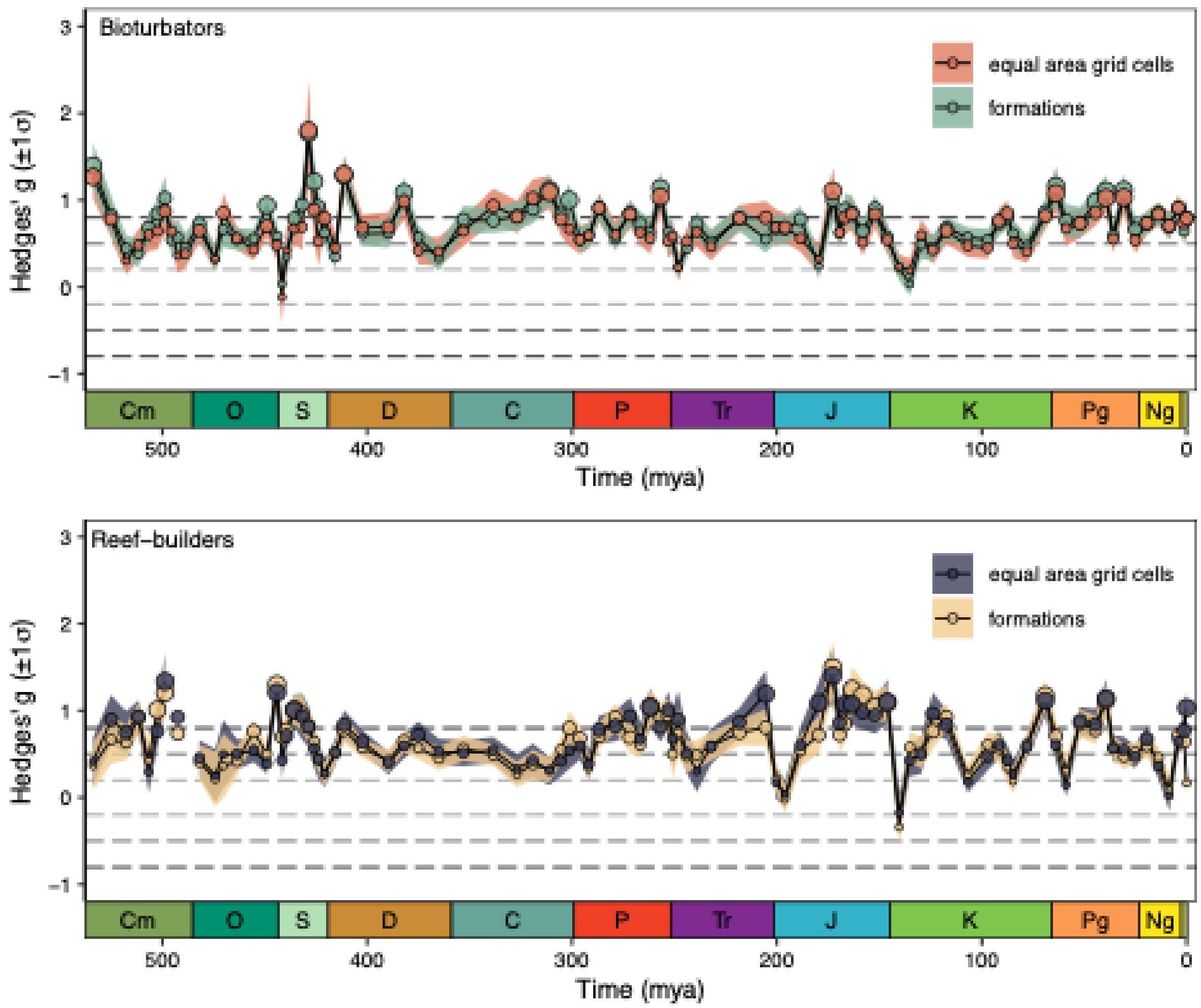

We have added the following text to the methods to address these updates:
"To further address the impact of spatial biases, we conducted a second set of analyses in which we divided our data into equal-area grid cells, subsample 700 random fossil occurrences per stage, and then 10 random fossil occurrences per grid cell (Supplementary Figures 8,9). We find negligible differences between this protocol and the formation-level subsampling (Supplementary Figure 10), likely because most of the fossil occurrences form the same formations are not geographically widespread." *Lines 476-481*

Methods – effect size analysis:
General: is there enough data to fit a/some GLMM(s)? If possible, I think it would strengthen the results of this study to be able to see the variance in Shannon's Diversity Index apportioned to ecosystem engineer diversity while controlling for the impact of global average temperature, water depth, and palaeoenvironment. From the looks of Supplementary Figures 3 and 5, the latter two may have an impact which is currently unaccounted for. A GLMM approach would also allow you to model geographic location of samples as a random effect if you rasterised the data as suggested above. I should stress that this is not something that I think is

mandatory – the only aspect of this that I think *needs* addressing is the role palaeoenvironment plays.

Changes not made: Thank you for the suggestions – unfortunately this approach is not statistically feasible for our dataset, as effect sizes are aggregated at the stage-level and cannot be rasterised because they represent the comparison between two distributions of formation-level data (in other words, effect sizes are aggregate data that lack a spatial component). Moreover, we would be hesitant to over-interpret the Scotese et al. (2021) GATs in this way as they only represent global average temperatures and should not be extrapolated to rasterised ecological data. One could, theoretically, use GLMMs to investigate mixed effects on ecosystem engineering impacts if each formation is associated with ecosystem engineering diversity, community-level non-ecosystem engineer diversity, bathymetry, lithology, and local climate variables (through either local geochemical proxies or paired to palaeoclimate reconstructions). This would be a really promising avenue for future research, but would require extensive additional data compilations and analyses, and so we believe it is currently out of scope for this study. We absolutely appreciate the idea, though.

Changes made: With regard to the palaeoenvironment effects on Hedges' g, we agree that palaeoenvironment certainly does have some impact on Hedges' g. However, we still find that constraining within equivalent environments and bathymetry does not *reduce the effect sizes to insignificance*, which we still interpret to mean that ecosystem engineers do have an effect on biodiversity, but is not mutually exclusive to palaeoenvironments modulating or enhancing ecosystem engineers. To clarify our interpretations, we have included the following text:

"**While we find some differences in the overall trends of effect sizes through the Phanerozoic found between different palaeoenvironments, positive effect sizes are still present when accounting for palaeoenvironmental differences, so we cannot attribute impacts on biodiversity to lithology or bathymetry alone** (Supplementary Figures 2-5)." *Results: Effects on Phanerozoic Biodiversity, Lines 99-102*

"**Our within-palaeoenvironment analyses also indicate some differences in effect sizes between lithologies and bathymetries (Supplementary Figure 3, 4). While the trend of significantly positive effect sizes persists in constrained palaeoenvironments, we do find occasional significant differences. For example, only comparing carbonate environments to calculate effect sizes of reef-builders results in occasionally more dynamic effect size changes, and effect sizes in deep marine settings of bioturbators more often decline to significantly negative. These specific differences that arise in unique palaeoenvironments warrant further investigation, but suggest that an ecosystem engineering group's effect is not always equal across all environments throughout Earth history."** *Discussion, Lines 352-360*

Line 464: I actually think Supplementary Figures 3 and 5 (maybe 4 and 6 as well) belong in the main text, perhaps combined into a single figure somehow. Just a personal opinion but I think there's something to them worth discussing (see above).
Changes not made: Thank you for the suggestion, but attempts to combine these figures into a single main text figure is difficult in terms of clarity. We opt to keep these in the supplement, especially considering they are not as often discussed in the main text when evaluating our hypotheses. If the editor disagrees, we would defer to their comments and opinion.

Methods – climactic and evolutionary correlates:
General: as with above, I think there is a place for rasterization in this workflow to ensure spatial biases aren't compromising these results.
Changes not made: We again thank you for the suggestion. As explained above, because our effect sizes are aggregated within time bins, they lack spatial data, so this is not feasible for our dataset. Additionally, we would need spatial climate data.

Figures:
Figure 3: I'd suggest standardising either the duration of the time series or the number of sampled points across the 5 panels. At the moment, comparisons between the 5 are hindered by the differences in the above. Also, can you shrink the y-axis to maybe -1.25 – 1.25? Will make it easier to view changes in effect size through time, especially if 0 is at the midline.
Thank you for the suggestions. We have partially updated the figures:

Changes not made: We use different time intervals around each of the extinction boundaries based on general consensus of how long the extinction interval is (in many ways, only the K-Pg is a true hard boundary). For example, the end-Devonian has widely considered to be two-pulsed (whether or not this true or just the Signor-Lipps effect). In the wake of the end-Permian mass extinction, recovery is considered to last perhaps up to 10 million years. We therefor chose the stages to encapsulate the widest extinction and subsequent recovery intervals.

Changes made: We have updated the figure to expand the ranges of the end-Triassic mass extinction, to more accurate reflect that changes happen prior to the Rhaetian. We have also changed the y axes to -2 – 2 for clarity (anything below this does not leave room for the key in Panel B).

Figure 4. Panel B y-axis label cut off.
Changes made: Thank you for pointing this out! We've also noted the same issue in Figure 5. We have fixed the axis so that the text is not cut off.

Figure 6: Panels A & C,I like the colours but personally, think I'd find it easier to track the relationship between temperature and the effect size if average temperature was plotted as a separate curve. Just a thought!

Changes not made: Thank you for the suggestion! After discussion between ourselves and colleagues, we respectfully and opt to keep the current version. We note that the temperature curve for that data is available in the original Scotese papers as well, so it is not entirely necessary to produce a temperature curve here, as the focus is still the effect sizes.

Reviewer 2

This manuscript examines the effects of ecosystem engineers on marine biodiversity throughout the Phanerozoic. In particular, it emphasizes two primary types of ecosystem engineers that function on different levels: bioturbators in the sediment and reef builders above it in marine environments. The results show that both groups of ecosystem engineers have a positive effect on the Phanerozoic biodiversity.

The manuscript is well-written and easy to follow. The hypotheses are clearly stated. In the results and discussion, the authors comprehensively cover various aspects of biodiversity change associated with ecosystem engineers, including mass extinction events, climatic changes, and evolutionary trends.

The methods are detailed, with explanations and rationales for each statistical test, which ensures reproducibility. Various analytical approaches were implemented to address spatial and temporal sampling biases, and different sensitivity tests were employed to help mitigate these biases.

Overall, this manuscript significantly contributes to understanding the role of ecosystem engineers in marine biodiversity throughout the Phanerozoic.

Thank you very much for taking the time to review our manuscript. We so appreciate the question, comments, and ideas you have contributed in your review, and we are excited about the improvements made to the manuscript in response to your feedback.

General suggestions for improvement:

Lines 16-19: It sounds here that both groups of ecosystem engineers were analyzed together concerning their impact on the overall biodiversity. It would be helpful to clarify that the analyses were conducted separately for each group.
Changes made: Thank you for your suggestion. We agree this benefits from clarification, and we have now edited the text to read:
"Here, we investigate **how biodiversity was impacted by reef-building or bioturbating ecosystem engineers** at each stage of the Phanerozoic, including their ability to maintain biodiversity over mass extinctions, and identify extrinsic conditions that modulate their impacts."

Lines 81-84: Consider citing more recent publication on reef refugia by Dimitrijevic et al. (2024) https://link.springer.com/article/10.1007/s00338-024-02548-y#citeas
Changes made: Thank you for this suggested reference! We agree this is an excellent and perfectly appropriate reference for this line, so it is now included in the manuscript.

Lines 211-213: Similar to the abstract, this sentence sounds like you have analyzed both groups together. Speaking of which, what would the patterns be if both ecosystem engineer groups were analyzed together?

Changes not made: Thank you for the interesting question! If bioturbators and reef-builders are grouped together in the analysis, the following stage-level Shannon's Diversity values and Hedges' *g* were calculated:

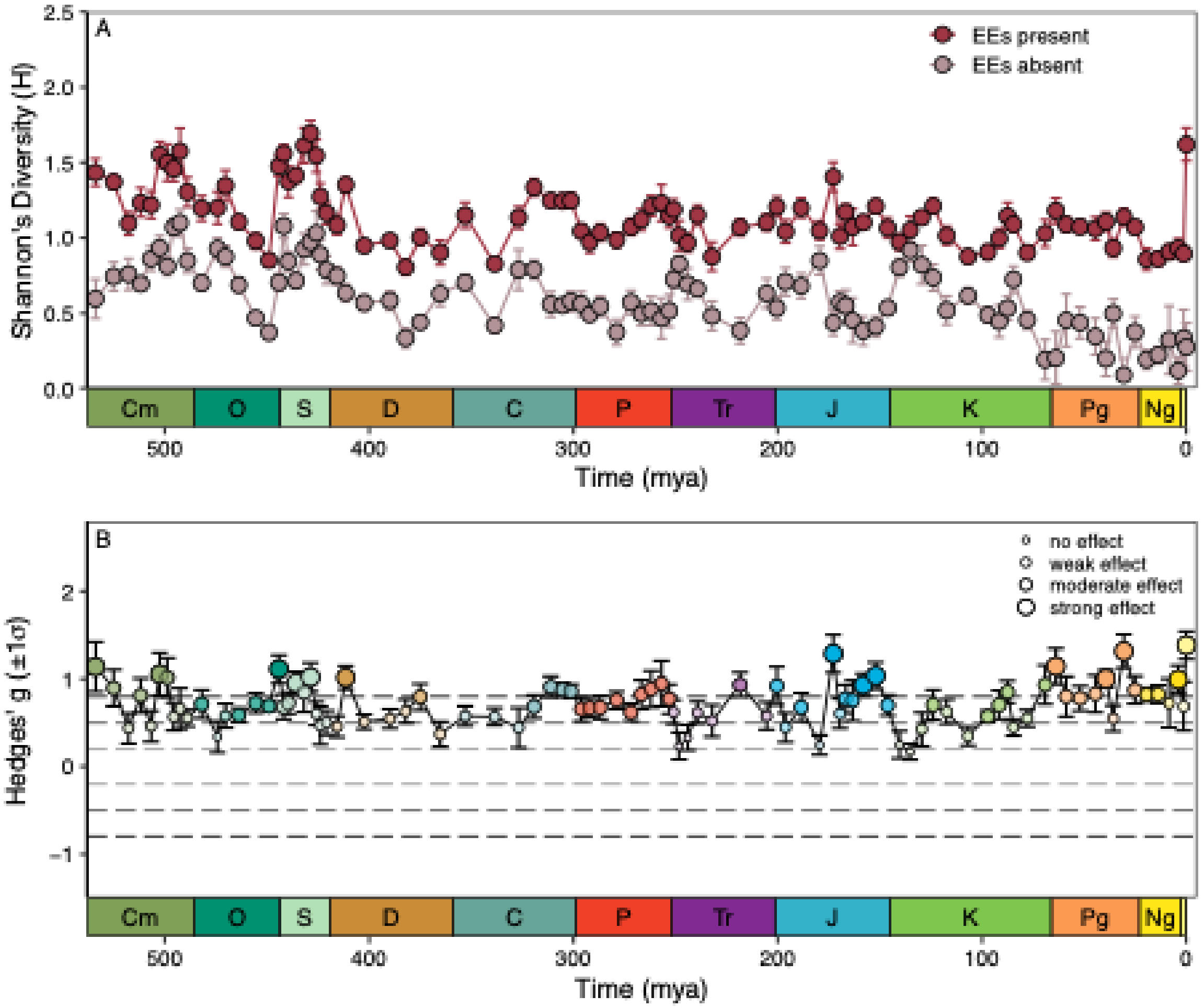

These results are consistent with the broad conclusions of these ecosystem engineers driving persistent significantly strong positive effect sizes throughout the Phanerozoic. The effect sizes are, unsurprisingly, a sort of middle-ground between the bioturbator and reef-builder effect sizes. We agree this is interesting, but we opt to keep it out of the manuscript. Reef-builders and bioturbators operate as ecosystem engineers through very different behaviours and processes. A strong bioturbator may, for example, stimulate phosphorus recycling or change the redox landscape of the sediments, in contrast to reef-builders' activities of habitat construction and increasing spatial environment heterogeneity (as non-exhaustive examples). Because these ecosystem engineering processes are so different between bioturbators and reef-builders, we believe these results are most appropriate kept separate.

Lines 248-249: Why "even" sponges when they play the same functional role in the ecosystem by providing a three-dimensional habitat? Also, did you, by any chance, compare the effect of corals only vs. sponges? It would be interesting to see the patterns of individual reef-building groups.

Changes not made: Thank you for these questions. In these lines, we aim to contrast corals to sponges, as corals have traditionally been more extensively discussed as ecosystem engineers both in the modern ecosystem engineering literature (see, for example, original discussions of "physical ecosystem engineering" in marine systems by CL Jones). However, we agree that the use of "even" here may diminish the ecosystem engineering impacts of sponges, so we have removed the word from this text. With regard to your question about comparing sponge impacts versus coral impacts, although this is certainly an interesting research question, we find it is out of scope for this particular paper. In the context of our research questions here, we would then need to compare not only sponges and corals, but all of the other reef-building ecosystem engineering groups to each other, and then the bioturbator functional groups (e.g., bioturbating suspension feeders, deposit feeders, grazers, and predators). This would be interesting in determining if some ecosystem engineering groups are more impactful than others, but so many additional analyses to make these comparisons would add sufficient length to this manuscript that we feel it is out of scope and would require a separate dedicated paper.

Lines 252-256: I do not understand the point you are trying to convey here regarding the reef builders. There were other reef builders before the scleractinian corals appeared in the fossil record, and Paleozoic corals also potentially hosted photosymbionts, see for example, Jung et al.
(2024) https://www.nature.com/articles/s41586-024-08101-9
And Zapalski et al.
(2017) https://www.sciencedirect.com/science/article/pii/S0031018217306855?via=ihub

Changes made: Thank you for your comment. We absolutely agree that Paleozoic corals and non-coral reef builders would have been key ecosystem engineers for hosting biodiversity hotspots, as we explain these lines of text. Our goal with this section is to justify these conclusions with the effect size analyses, as we have generally found a lack of discussion and perception of non-scleractinian corals as "strong" ecosystem engineers, perhaps because of their dominance in modern ecosystems. Arising from Kiessling & Flugel (2002)'s PaleoReefs database reef type classifications, there tend to be functional contrasts made between metazoan reef-builders that construct true reefs (rigid frameworks), reef mounds (abundant skeletal organisms but lacking a rigid framework), and biostromes (little to not depositional relief). In our experience, this has led to a strong underlying hypothesis in the field that organisms like rudist bivalves (see Reviewer 3's comment #9 for example) and stromatoporoids are "lesser" ecosystem engineers. This section is, therefore, here to demonstrate that non-coral ecosystem engineers were still incredible important in the

Cambrian and during coral reef gaps. While this may be obvious to ecologists and palaeoecologists whose work focuses on reefs, it is the co-authors' experiences in the broad field that this needs dedicated justification and explaining.

With regard to Paleozoic coral photosymbionts, we maintain that neither of these papers are entirely conclusive about Paleozoic corals in general. Moreover, this text is specifically referencing modern-type corals, which inarguably did not evolve until the emergence of Scleractinia. We agree this could be made more clear and does not need to be mutually exclusive with Paleozoic corals that may have had photosymbionts.

We have revised this text in line with these comments:
"Both bioturbators and reef-builders were thus ecologically important ecosystem engineers early in the Phanerozoic, perhaps earlier than expected given that modern-type bioturbation (e.g., large three-dimension network burrows and extensive deep sedimentary mixed layers) and reef-builders (**e.g., aragonitic, branching- and framework-building, zooxanthellate scleractinian corals**) did not arise until the Mesozoic. This indicates that engineers that may be hypothesised to have weaker impacts – **such as smaller, shallower invertebrate burrowers and reef mound- and biostrome-constructors** – still played critical ecological roles in the Paleozoic, and their impacts should be considered in the context of their own contemporaneous ecosystems and Earth systems states."

Lines 257-258: why are sponges considered as "weaker" ecosystem engineers? They built prolific reefs in the Paleozoic and through the Early Triassic (see Kiessling 2009 https://www.annualreviews.org/content/journals/10.1146/annurev.ecolsys.110308.120251 and references therein).
Change text
Changes made: Thank you for your question. See our comments above, and we hope that revised text sufficiently resolves your critiques.

Line 293-295: I wouldn't say this is surprising at all. Sponge reefs persisted well into the Early Triassic (i.e., "Permian heritage", See Flügel et al. 2002 Triassic reef patterns and Weidlich et al. 2003 https://pubs.geoscienceworld.org/gsa/geology/article-abstract/31/11/961/29201/Permian-Triassic-boundary-interval-as-a-model-for?redirectedFrom=fulltext) so that the functional role of reef builders was not compromised despite the coral reef gap. So there is, basically, the opposite situation from the one described in the previous lines of the discussion at the end-Devonian when the coral reefs functionally replaced stromatoporoid reefs.
Changes not made: Thank you for your comments. We respectfully disagree that no collapse in effect size is not surprising across the EPME, even with the persistence of non-coral reef-building ecosystem engineers that provided the necessary ecological resilience when corals were lost. We argue that the persistence of effect

sizes is still surprising due to 1) the environmental impacts of the EPME, and 2) the severe decline in metazoan reef-builder diversity during the EPME (as is clear in, for example, Flúgel & Kiessling (2002) , as we cite).

Line 299: I suggest citing other references here and elsewhere apart from one own. For example, Kiessling, Flügel, etc.
Changes made: Thank you for the suggestion. We agree and have added the relevant citations fo Flugel & Kiessling in line with references earlier in this section.

Line 303-305: Or this signal is visible because they are the dominant reef builders at this time while other periods have multiple reef builders that mask the signal by replacing the functional roles. It would be great to test this hypothesis by analyzing the effects of individual reef-building groups.
Changes not made: Thank you for the comment. Again, we do find this interesting but we feel out of scope for this paper that is focusing broadly on ecosystem engineering and includes bioturbators, and we are dedicating other manuscripts to questions like these comparing groups to understanding the evolution of ecosystem engineering 'strength,' so to speak (see our previous comments).

Lines 409-412: I don't quite understand the reasoning for selecting these 10 particular reef-building groups, and why do you consider some as more important than others, for example, you selected encrusting tube worms, but why not bryozoans or algae? An explanation of the reasoning why some reef-building organisms are excluded would be appreciated.
Changes made: Thank you for your question. First, we use the same dataset that we used in Cribb & Darroch (2024), and this is largely for consistency across projects. These reef-building groups were originally chosen from the reef taxonomic groups included in the PaleoReefs dataset: "Micbrobes; algae; foraminifera; stromatoporoids; hydrozoans; chaetetids; archaeocyaths; "pharetronid" sponges; siliceous psonges; corals; pelecypods; worms; vermetid gastropods, problematic tubes; "*Tubiphytes" (=Shamovella)*; bryozoans; brachiopods; and pelmatozoans." We omit microbes, algae, and foraminifera, as we only focus on metazoan reef-builders. We also remove any problematic taxonomic affinities and outdated taxonomic affinities (e..g, Pelmatozoa). We did not include brachiopods due to a lack of information or agreement about propensity for reef-building in certain groups (we do note that some brachiopods though are included as bioturbating reef ecosystem engineers due to ecospace information). We did not include bryozoans due to their primary encrusting characteristics over three-dimensional reef-building. Finally, we only included taxonomic groups for which we could obtain PBDB data with sufficient stratigraphic metadata. This ultimately left us with our ten reef-building groups. We note that a fairly similar approach was taken by R. Close et al (2025), so this dataset will ensure comparability and reproducibility across projects and research groups. We have clarified our approach in text:

"We focused on ten key reef-building metazoan groups **from the PaleoReefs[52] database that are represented in the PBDB with sufficient metadata and lack ecological and taxonomic uncertainty**[40]: archaeocyathids, hexactinellids, stromatoporoids, reef-building hydrozoans, tube worms (serpulids and sabellarids), chaetetids, rudist bivalves, tabulate corals, rugose corals, and scleractinian corals. We note that this group includes reef-builders that construct a range of reef types, including true reefs, reef mounds, and biostromes[52]. **Furthermore, we note that we may lack some reef-building ecosystem engineers in this dataset, including non-metazoan reef-builders like stromatolites and calcareous algae, and future work could include and compare the effects of these other reef-builders. Finally, we note that this list includes a variety of reef functional types, from constructors of true reefs, to biostromes, to reef mounds.** Despite this functional diversity, all groups should have reef ecosystem engineering impacts at some level, from hydrodynamic effects to water column filtering."

Also, not all reef-related organisms contribute to reef building, such as solitary scleractinian and solitary rugose corals. Even considering the "true" reef builders (i.e., colonial corals), their occurrences can often be part of the bottom-level communities and not build reefs. It would be great to highlight this in the discussion as part of the potential biases of the study.
Changes made: Thank you for the comment. We agree – and this is also true for bioturbators, which were identified based on their ecological affinities for the possibility of mixing sediments but, because we use their body fossils and not trace fossils, does not necessarily mean the bioturbators were always doing their most effective hypothesized ecosystem engineering process. To make this point, we have added the following text when discussing our dataset assembly:
"**Finally, we note that for both bioturbators and reef-builders, these identifications are based on the hypothesized propensity for the animal to do their ecosystem engineering behaviours. Direct evidence of ecosystem engineering processes is absent from the body fossil record, so these identifications are only an inference of ecosystem engineering. Here, we assume that if an animal can possibly do their typical ecosystem engineering behaviours (e.g., corals constructing true reefs, or infaunal bioturbators significantly mixing sediments), then those associated ecosystem engineering processes did occur."**

Lines 412-413: Please be careful with terminology; you did not analyze reefs but rather reef builder occurrences. As I mentioned above, reef-building organisms do not necessarily build reefs, and it should be clarified that reef-building occurrences are not synonymous with reefs.
Changes made: Thank you for the comment. We agree, and we have located any instances when we say reef or reef ecosystem instead of reef-builder and have appropriately changed and clarified the text.

Lines 423-425: Please clarify why the standardized subsampling number was set to 700.
Changes made: Thank you for the suggestion. We have added the following text: "To

account for this bias, we standardize the number of fossil occurrences in our analyses through time by randomly subsampling 700 fossil occurrences **(set below the smallest stage-level population size of fossil occurrences)** in each stage through the Phanerozoic."

Lines 465-467: It's unclear if the dataset with bioturbators includes reef builders if the pool without bioturbators has reef builders, and vice versa. I recommend rephrasing this section for clarity and labelling the datasets with small Roman numerals or letters.

Changes made: Thank you for the suggestion. We note that we do refer to the dataset statistics later in this section with x_i, n_i, and s_i, where i=1 for the pool of formations with ecosystem engineers and i=2 for the pool of formations without ecosystem engineers, and we use a BP/RP and BA/RP notation in the supplemental to note Bioturbators/Reef-builders Present or Bioturbators/Reef-builders Present, so our datasets are notated for our equations. To your first question, we have clarified with the following text:

"For each stage, data is divided into two pools: fossil occurrences in formations where either bioturbators or reef-builders are present, and fossil occurrences in formations where they are absent. **We do note that in these absence pools, the other type of ecosystem engineer (and non-bioturbator or non-reef building ecosystem engineers) are present, so that this approach tests directly for the effects of a single ecosystem engineering group rather than ecosystem engineering as a whole."**

Figures:

Please explain the error bars in the captions.

Changes made: Thank you for the suggestion. We have added this to the captions where necessary.

Supplementary Figure 1: are the shaded areas confidence intervals? Please state so in the figure caption.

Changes made: Thank you for the question. Yes, these are the mean ±1 sd, just as the error bars in the main text figures. We have added this to the caption.

Supplementary Figures 2 and 4 are hard to read; you could try with proportional polygon plots.

Changes made: Thank you for the suggestion. We are not entirely sure what is meant by proportional polygon plots, but we have updated these using geom_area() instead of the stacked bar graphs. We hope this is more clear.

Minor comments:

• Supplementary Table 4 is marked as 3

Changes made: Thank you for pointing this out. We have updated the table correctly.

• Line 83: should be environmental stress

Changes made: Thank you for pointing out this typo. We have corrected the text.

• Line 455: Lagerstätten is misspelled (i.e., ä is in the wrong place), and the word's first letter should be capitalized.
Changes made: Thank you for correcting this. We have updated this text.

• Line 701: there is a point missing at 0.8 in the figure caption
Changes made: Thank you for noting this typo. We have corrected this.

Reviewer #3 (Remarks to the Author):

Reviewer's report on manuscript by Cribb et al., entitled: "Marine Phanerozoic biodiversity increased in presence of ecosystem engineers".
1) General comment: This is the interesting paper, which has analyzed fossil communities with and without ecosystem engineers to calculate the impact of the presence of ecosystem engineers throughout the Phanerozoic. Although ecosystem engineers were thought to have affected ancient ecosystems and biodiversity, quantitative estimation had not yet been explored. The author's study indicates that ecosystem engineers are significantly associated with increased biodiversity base on Paleobiology Database.

Thank you for your comments! We greatly appreciate the time you have taken to review the manuscript. We found your questions interesting and insightful, and your feedback has improved the quality of the manuscript. Below, we have responded to your comments and questions, providing examples of the updated, edited, or added manuscript content where relevant.

2) Lines 46-49: "Moreover, as recent advances have underscored the importance of local- and regional-scale biotic changes in driving global Phanerozoic biodiversity trends15,16, there is a need to understand the importance and impacts of these key biotic interactions."
Comment: Although the authors state that local- and regional-scale biotic changes impact biodiversity, this sentence is unnecessary because the paper does not address local- and regional-scale biotic changes.

Changes not made: Thank you for the feedback. We respectfully disagree and have opted to keep this text, as ecosystem engineering impacts are local in scale and our analyses focus on community-level ecological analyses and change, in contrast to more 'traditional' palaeontological approaches (particularly using the PBDB) of globally aggregated data to construct biodiversity times series. We feel this is important context for rooting this work in the focus of regional-, local-, and community-scale palaeoecological dynamics focus rather than the focus of drivers of globally aggregated biodiversity curves.

3) Lines 106-110: "There are notable effect size peaks in the Katian during the Ordovician ($g=0.94\pm0.13$) in the Homerian ($g=1.77\pm0.27$) during the Silurian, in the Pragian ($g=1.28\pm0.21$) and Givetian ($g=1.08\pm0.12$) during the Devonian, in the Wuchiapingian during the Permian ($g=1.14\pm0.19$), and in the Danian ($g=1.16\pm0.23$) during the Paleogene. While there are significantly strong positive effect sizes…".
Comment: What are the notable effect sizes and significantly strong positive effect sizes? Why are the Cambrian Guzhangian ($g = 1.02 \pm 0.25$) and Jurassic Aalenian ($g = 1.00 \pm 0.25$) not included here"

Changes made: Thank you for the question. The Guzhangian and Aalenian are not included in this list because the lower uncertainty bound on both *g* values are below 0.8. We define our thresholds for significance in *Methods: Effect size analyses*:

"Finally, Hedges' *g* has rule-of-thumb thresholds for effect size[53]: weak effects are considered where $0.5 > |g| \geq 0.2$, moderate effects are considered where $0.8 > |g| \geq 0.5$, and strong effects are considered where effects are $|g| \geq 0.8$. However, these thresholds are context-dependent, so it is important to consider the effect sizes in the context of other ecological studies of ecosystem engineers [54]. Other meta-analyses of ecosystem engineers use the same thresholds and may consider significance where $|g| \geq 0.2$ [25,52], but to be conservative for the fossil record, **we only consider effect sizes to be significant where they meet strong and moderate thresholds ($|g| \geq 0.5$) and uncertainty does not cross the weak-moderate threshold**."

4) Lines 138-141" Effect sizes rapidly decline to insignificant or even weakly negative values most notably during the early Jurassic (Hettangian g=0.22±0.17; Sinemurian g=-0.02±0.17, the Berriasian (g=-0.34±0.23), the Albian (g=0.26±0.12), and the Santonian (g=0.19±0.13) (Supplementary Table 4)."
Comment: Isn't the Upper Miocene effect size (g = 0.09 ± 0.16) included here?

Changes made: Thank you for the comment and question. Yes – this is not an exhaustive list of insignificant or weak effect sizes but instead note large magnitudes of change to low effect sizes between preceding stages, but we agree the Upper Miocene should be included in this list. We have edited the text accordingly.

5) Lines 192-195" While we do not find a clear, robust relationship between temperature and effect sizes, the strongest effects of bioturbators do occur in moderate global temperatures in the range ~16-22°C, and effect sizes decrease as the climate warms or cools towards extremes (Figure 6B)."
Comment: The authors state that 'they did not find a clear relationship between temperature and effect size.' However, in lines 25-26, the authors mention that ecosystem engineers impact climate. There is no clear impact, but climate modulates ecosystems. What does this mean?

Thank you for question – we would like to clarify. In lines 25-26, we state: "our results reveal that climate can modulate ecosystem engineer impacts, with both bioturbators and reef-builders exhibiting strongest effect sizes within an optimal moderate temperature range" meaning climate can control on how impactful ecosystem engineers are. As we explain in the discussion, this most likely arises because ecosystem engineers either cannot increase resource availability to other organisms in stressed environments, or the resources they do impact have little relative importance in the face of severe physiological stress driven by temperatures and oxygen. We emphasize that this does not mean, and we do not conclude, that we find any evidence that ecosystem engineers impact climate. As further explained in the main text, the cross-validated

LOESS curves suggest a non-linear relationship between ecosystem engineering impact and climate, but we believe the burden of proof for determining the causes of ecosystem engineering impact magnitude should be quite high, which is why we take a cautious relatively conservative interpretation of the results.

Changes made: We have edited lines 25-26 in the abstract slightly:

"Finally, our results reveal that climate **may** modulate ecosystem engineer impacts, with both bioturbators and reef-builders exhibiting strongest effect sizes within an optimal moderate temperature range."

6) Lines 238-240: "For example, the Homerian effect size peak is most likely driven by the invasion of deposit feeding ecosystem engineer."
Comment: Precisely, which groups contributed as deposit feeding ecosystem engineer?

Changes made: Thank you for your question. The primary group is nuculoid bivalves, an order of generally rapidly-moving deposit feeding bivalves (Liljedahl 1994). We have updated the text: "For example, the Homerian effect size peak is most likely driven by the invasion of deposit feeding ecosystem engineers, which include **rapidly burrowing deposit-feeding bivalves**"

7) Lines 247-249: "We find this is not limited to coral ecosystem engineers; even sponge reef ecosystems apparently had significant impacts on biodiversity."
Comment: Does this simply mean that stromatoporoid sponges dominating Devonian reefs also indicate a positive effect on biodiversity? Devonian stromatoporoid reefs include tabulate corals as well. Could these reef ecosystems be called sponge reef ecosystems?

Changes made: Thank you for the question. We interpret this question arising from our lack of clarity about reef-builders, reefs, and reef ecosystems terms, as Reviewer 2 rightfully pointed out. We have edited this text to read the following:

"We find this is not limited to coral ecosystem engineers; **even sponge reef-builders such as hexactinellids and stromatoporoids** apparently had significant impacts on biodiversity."

We hope this clarifies that we are specifically referring to sponge reef-builders, which yes, include stromatoporoids. We do not distinguish between the effects of sponges versus corals (we believe this is out of scope for this particular manuscript, as we defend in our responses to Reviewer 2). But to your specific question, yes – the stromatoporoid sponges that dominate Devonian reefs do have positive associations with biodiversity, but as a whole, this time period only has weak effect sizes, which curiously contrasts our traditional understandings of the Devonian "reef peak" during this time period.

8) Lines 249-251: “Our results illustrate positive effect sizes during intervals of the Cambrian when reefs are made entirely of hexactinellid sponges, and also during the Hirnantian, …”
Comment: The hexactinellid sponge-bearing Cambrian reefs included dominant microbial components. Did the hexactinellid sponges primarily affect biodiversity? The transition from microbial-dominated to metazoan-dominated reefs occurred in the Ordovician. To what extent did the increase in ecosystem engineers, such as metazoan reef builders, impact biodiversity during this interval?

Changes made: Thank you for the question. Our analyses do not allow us to contribute effect sizes to one group over another, so we cannot confirm whether it is the hexactinellid sponges that primarily impact biodiversity over the other types of reef-builders (this would be an interesting study comparing ecosystem engineering effects of different reef-builders, but we believe it is out of scope for this particular manuscript, as we have also explained in our responses to Reviewer 2). We do not analyse microbial reef-builders because we lack data from the PBDB, so this effect is entirely driven by metazoan reef-builders. We note that we have confirmed throughout the manuscript already that we are focusing on reef-builder metazoans (e.g., the first line of the discussion), but we have also clarified this in the introduction and methods:

Introduction – “Here, we investigate the role that marine bioturbators and **reef-building metazoans** – two of the most important ecosystem engineering groups in modern oceans [13] – have had on biodiversity, providing the first quantitative test of the hypothesis that ecosystem engineers over the Phanerozoic acted as key drivers in increased biodiversity [20]”

Methods: Dataset assembly – “We focused on ten key **reef-building metazoan groups** [39]: archaeocyathids, hexactinellids, stromatoporoids, hydrozoans, tube worms (serpulids and sabellarids), chaetetids, rudist bivalves, tabulate corals, rugose corals, and scleractinian corals [51].”

9) Lines 268-270: “For example, much of the weak and insignificant effect sizes during the Cretaceous are associated with the rise of rudist bivalve reefs39, which were apparently less efficient ecosystem engineers in terms of driving increased biodiversity (Figure 2). “
Comment: The rudist bivalves are relatively simple and they do not create “true reefs” in the same way that corals and sponges did.

Changes made: Thank you for your comment, and we agree, and we have added the following text:

“For example, much of the weak and insignificant effect sizes during the Cretaceous are associated with the rise of rudist bivalve reefs [39], which were apparently less efficient ecosystem engineers in terms of driving increased biodiversity, **likely because they did not**

build ‘true reef’ structures with similar levels of complexity relative to same-age corals and sponges”

10) Lines 293-300: “The persistence of reef effects through the end-Permian mass extinction is particularly surprising given its associated coral reef collapse44. Reef-associated global diversity certainly significantly declined during this interval. However, our formation-level results indicate this would have been primarily due to the loss of the number of reef ecosystems (Supplementary Table 3), rather than the inhibition of surviving reef-builders’ ecosystem engineering strength, as other non-coral reef-builder survivors in the early Triassic39 maintained communities where biodiversity was still, on average, higher than in non-reef communities (Figure 2A).”
Comment: What does the inhibition of surviving reef-builders’ ecosystem engineering strength means? Very a few sponges are known from the microbial reefs during the early Triassic. Did these rare sponges contribute as ecosystem engineers?

Thank you for your interesting question! The inhibition of ecosystem engineering strength would refer to some sort of environmental-driven effect that causes an individual ecosystem engineer to lose its ability to significantly impact resource availability. As an example here, we note that this is being observed today for modern reefs (Wild et al., 2011). The ecosystem engineers in question here are actually serpulid worms – they seem to be quite cosmopolitan in the Induan, and we have found in other research that they were the primary metazoan reef-builders in the wake of the end-Permian mass extinction. Serpulid reefs still occur today – probably the most famous of which in Loch Creran, Scotland – and they have similar habitat-forming ecosystem engineering effects to patch-forming coral reefs. In the Olenekian, the hexactinellid sponges re-appear, along with the *Tubiphytes* “hydrozoa” which is enigmatic but likely represents some encrusting reef-building sponge or coralline algae. We discuss these early Triassic reef-builders in Cribb & Darroch (2024) To your question, yes – these all would have contributed to their communities as ecosystem engineers, despite being rare.

11) “Hedges’ g values are not possible for the Paibian, where only one formation contains reef-building ecosystem engineers (so s cannot be measured), and for Stage 10, when a metazoan reef gap occurred. “
Comment: Is the metazoan reef gap is Stage 10 real? Lithistid sponge-bearing microbial reefs are known to exist.

Changes made: Thank you for raising this point! We have read papers by Lee et al. showing these sponge-bearing reefs. As with the Paibian, we cannot calculate a Hedges’ g value for Stage 10 because the Notch Peak Formation is the only reef-bearing formation. We have updated the text in the caption of this table: “Hedges’ g values are not possible for the Paibian **or Stage 10**, where only one formation contains reef-building ecosystem engineers (so σ cannot be measured).” This does not change our effect size results, but we have updated the text to remove reference to a Stage 10

reef gap. We have also updated the datasets to include the Notch Peak Formation data Supplementary Table 3 to update Shannon's Diversity in formations with and without reef-builders (H_{RP} = 2.1±0.19, H_{RA} = 1.04±0.06), and Figure 2 to reflect these results.

12) Supplementary Table 3, "n1 =6 of the Cambrian Fortunian".
Comment: Which metazoans are identified as metazoan reef builders? Archaeocyaths were not present in the Cambrian Fortunian.

Thank you for the question. Yes, there are no Archaeocyaths in the Fortunian. These are all hexactinellid sponges, primarily belonging to the order Reticulosa.

13) Supplementary Table 3"
Comment: What types of reef-builders were identified in n1=12 and 10 of the Triassic Induan and Olenekian? Was a metazoan reef gap was previously thought to exist in these intervals?

Thank you for this question. See our response above about the serpulid reefs in the Induan and the sponge reefs in the Olenekian. There is only a coral reef gap in the Early Triassic – this is a common misconception largely to do with disagreements about the definition of reefs, but there are certainly non-coral metazoan reef builders that persist in the Early Triassic in addition to the microbial reefs.

1) L205; "moderate global climate between ~20-23°C (Figure 5D). " (Figure 5D) should be changed to (Figure 6D).

Changes made: Thank you for pointing out this error. We have amended the text accordingly.

2) Supplementary Figure 4" Palaeoenvironments classed as siliciclastic (purples) or carbonates (pinks), and shallow depths (medium), deep depths (dark), or unclassified depths (light).

We apologies, but we are not sure what this comment is referring to, but we have double-checked the captions for Supplementary Figure 4 and 2, and we do not find any inconsistencies between the palaeoenvironments legend and the figure captions.

3) Supplementary Table 1, "HBA = mean Shannon's Diversity across formations where bioturbators are present;" HBA revise into HBP.

Changes made: Thank you for pointing out this error. We have fixed this table caption.

4) Supplementary Table 3 , "Comparison of Shannon's Diversity (H) in formations

with (HRA) and without (HRP) reef-builders” HRA and HRP revise into HRP and HRA

Changes made: Thank you for pointing out this error. We have fixed this table caption and the same error that was in the column names.

5) Supplementary Table 3. “Results of effect size analysis for the presence of reef-builders. “Supplementary Table 3 revise into Supplementary Table 4

Changes made: Thank you for spotting this error. The supplementary table title has been corrected.

Reviewer 4

This manuscript presents a compelling investigation into the role of bioturbating and reef-building ecosystem engineers on marine biodiversity across the Phanerozoic. The analysis leverages fossil occurrence data and modern ecological methods to provide a quantitative assessment of the biodiversity impacts of ecosystem engineers over geologic time. The study's strengths include its novel focus, rigorous statistical treatment, and integration of paleoecological and climatic perspectives. Additionally, the authors provide a well-rounded discussion, highlighting the limitations of their analysis while emphasizing key findings: (1) Consistent and significant positive relationship between the presence of bioturbating and reef-building ecosystem engineers and biodiversity across most of the Phanerozoic. (2) The two groups of ecosystem engineers responded differently to mass extinction events, with their effects on biodiversity varying depending on evolutionary context and ecological conditions. (3) While the diversification of these groups does not appear to directly explain the observed trends, the authors suggest that climate likely played a role in modulating their influence on biodiversity, particularly under conditions of moderate global temperatures. This thorough consideration of both the strengths and constraints of the analysis enhances the robustness of the study's conclusions.

The authors show methodological rigor: i.e. subsampling protocols and effect size analyses are well-detailed, ensuring transparency and reproducibility. Moreover, the the results emphasize the fundamental role of biotic interactions in shaping marine biodiversity and provide insights into how ecosystem engineering influences biodiversity under varying climatic conditions. Moreover, the figures effectively display trends and results across the Phanerozoic, though some refinements could enhance their interpretability (see suggestions below). Thus, I believe the authors should be proud of their detailed analyses and robust discussion. The integration of paleoclimate and biodiversity dynamics provides a comprehensive narrative that will resonate with both paleoecologists and macroecologists. Addressing these minor suggestions will further strengthen the clarity and accessibility of this excellent study.

Thank you for your review and kind comments. We greatly appreciate the time you have taken to read and provide feedback on our manuscript. Below, we have responded and made changes in accordance with your review. We also note that, in line with the other reviews, we have updated our methodology descripts and GitHub repositories to maintain our levels of methodological transparency and ensure reproducibility of any new analyses.

Suggestions for Minor Changes:
Adding Age/Time to Geological Periods:
Include the approximate age range of each geological period mentioned throughout the text (e.g., "Cambrian (~541–485 Ma)") to assist readers unfamiliar with the names in geologic time scales.
Changes made: Thank you for the suggestion. We agree this will be helpful for a broad readership. As our results are stage-level, we have given the approximate upper and lower ages boundaries of stages defined by the International Commission on Stratigraphy when stages are first mentioned in text throughout the results section.

Figures 1 and 2 could be merged. Combining Figures 1 and 2 into a single figure with consistent formatting for both bioturbators and reef-builders would streamline comparisons. Hedges' g Plot could be simplified, i.e. standardize the size of data points and use gradients or horizontal lines to emphasize effect size trends, shown per see on the Hedges's g value on the y axis. This adjustment would maintain clarity while reducing visual clutter.
Changes not made: Thank you for the suggestion. We agree in theory, but we do find that combining these figures into one that would fit across two columns of text as per journal formatting would cause the individual points to be far less readable.

Figure 4 (Global Generic Richness):
Clarify whether "global generic richness" is measured or directly observed. If derived from models, make this explicit. Confirm whether the term "generic richness" consistently refers to "genera richness" or species-level data. A consistent term will avoid confusion.
Changes made: Thank you for the suggestion. We agree this caption would benefit from clarification, so we have edited the caption to now read: "A) Global (stage-level, across all formations) generic richness" to clarify that this measure of generic richness is not the average formation-level richness as it was measured for the effect sizes."

Figure 6 (Bioturbators vs. Reef Builders):
Add a clear title indicating the figure's focus on comparing bioturbators and reef builders, including a brief note about their respective impacts.
Changes not made: Thank you for the suggestion. We have opted to not add titles to stay in line with the format of the other figures following journal style, but we have further clarified comparison of bioturbators and reef-builders in the caption

Reviewer #1 (Remarks to the Author):

I would like to thank the authors for engaging with my comments so thoroughly and constructively, and apologise for the delay in my review of the revised version of this manuscript. All issues I initially raised have been satisfactorily addressed through editing, additional analyses, or thoughtful responses. Whilst I cannot speak for the author's responses to the other reviewer's comments (as I have not reviewed them in the interest of expediting the return of my review), from my point of view, the manuscript is ready for publication. I recommend it for acceptance.

If the other reviewer is not satisfied with the author's responses and so an additional round of revisions becomes necessary, then I think a great addition to this manuscript to bulletproof the results would be additional sensitivity tests where occurrences are rasterised and grid cells that don't meet some quality criteria (e.g., minimum number of occurrences, minimum number of genera, etc.) are filtered out before the 700 occurrences are drawn from each stage as part of the normal pipeline. Something similar for the formation analyses would be reassuring as well (i.e., the application of quality criteria to the formations before the dataset is analysed). HOWEVER, I stress these sensitivity tests are not essential. Just something I think would be worth checking if there are other issues that necessitate a second round of revisions.

Congratulations to the authors for a job well done!

Best wishes,
Tom Roper-Smith
Museum of Natural History
University of Oxford

Response to reviewer:

Thank you for your comments! Regarding your suggestion, we have run a set of analyses in undersampled regions (formations or grid cells with fewer than 20 occurrences) were removed from the dataset. We include the four sets of effect size results below with discussion of key points:

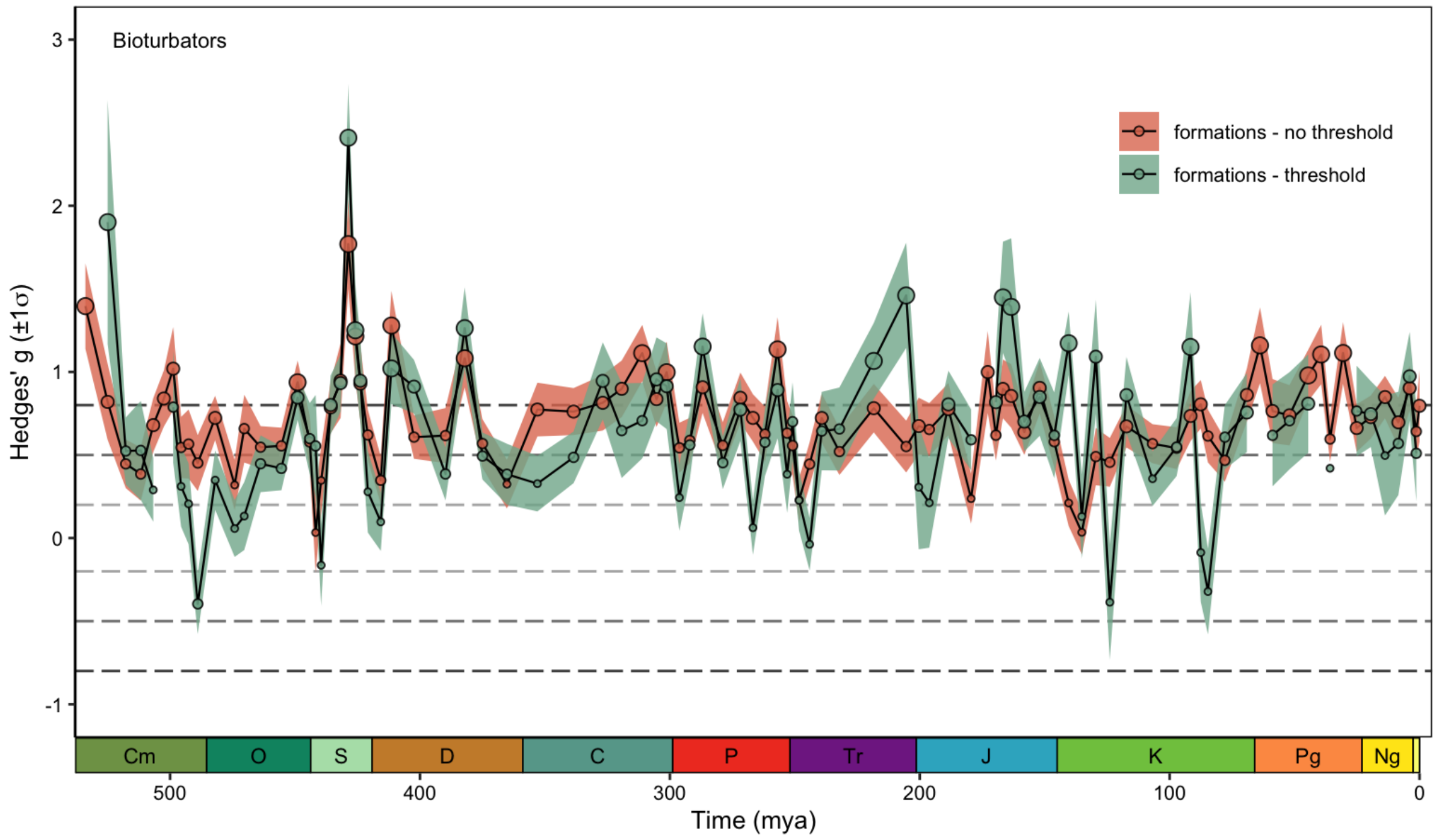

Removing data from formations where there were fewer than 20 occurrences per region results (green points and line) results in an effect size curve that is largely more dynamic than the full dataset. It is also notable that we lose a few stages in the Paleogene because, after the Paleozoic, undersampled regions primarily do not contain ecosystem engineers – this makes sense, given the effects of the MMR on infauna. At first, these results may seem troubling – there are instances where effect sizes seem to plummet (the endo f the Cambrian and during the Cretaceous) and there are other time intervals where effect sizes seem to spike (the late Triassic and Jurassic). However, these are not, in reality, particularly significant changes – the effect sizes drops occur in time intervals where effect sizes were necessarily not strong to begin with, and the apparently new peaks occur where effect sizes where strong. Overall, we emphasize that the message of these effect sizes is **not the direction of stage-by-stage trends, but the persistent record of strong effect sizes through the Phanerozoic, which remain in this 'threshold' test.**

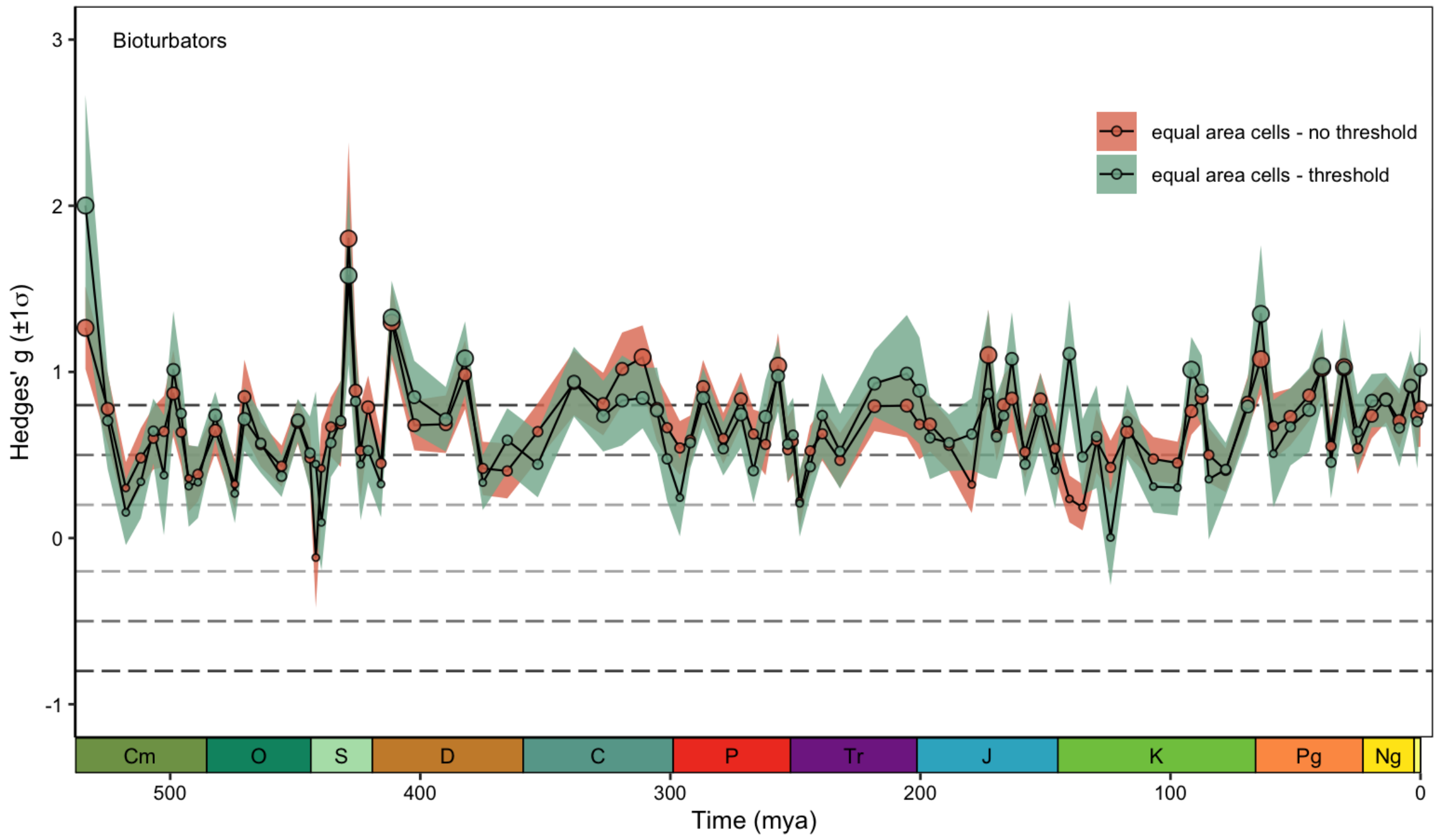

Interestingly, setting the 20 occurrences/grid cell threshold for the bioturbator effect size has little to no effect on the overall effect size results.

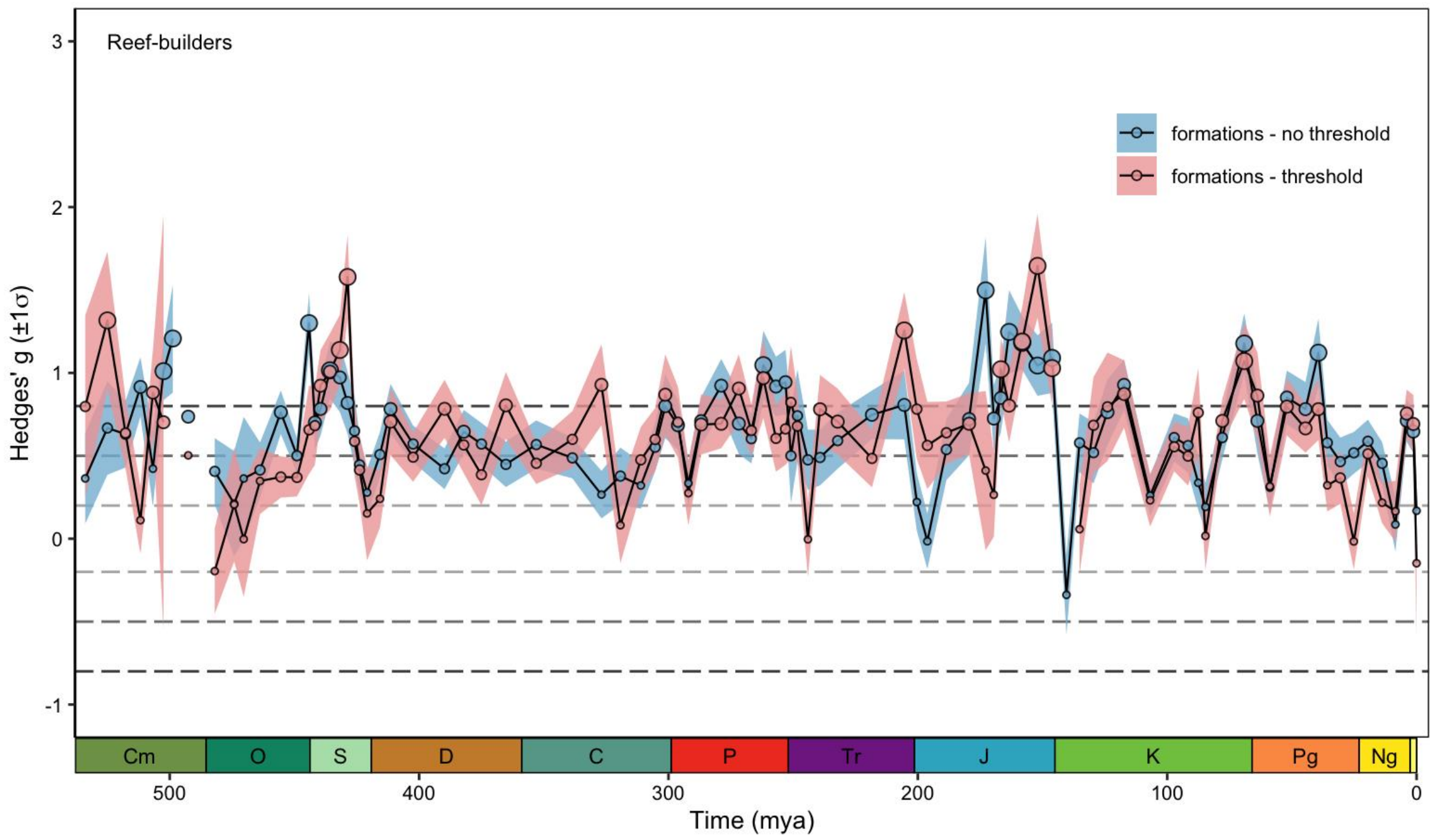

For the reef-builders, setting a threshold of 20 occurrences/formation has less of an impact than it does on bioturbators. Again, we lose some stages due to an abundance of undersampled non-reefal regions – this is a notorious issue at the

Jurassic-Cretaceous boundary, for example. A few strong effect size stages are lost during the Jurassic and early Paleozoic, but the message is largely the same.

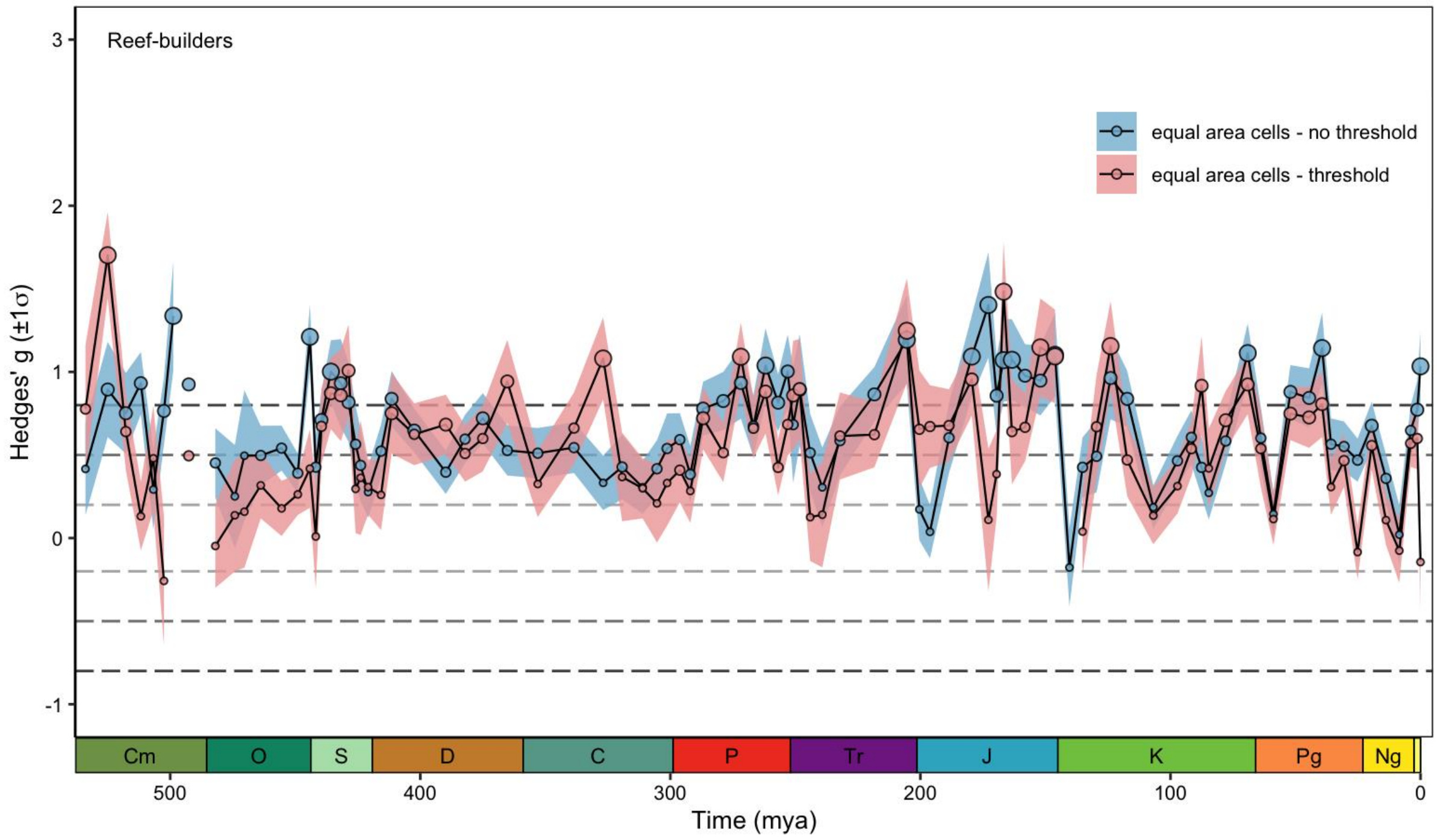

The threshold set on grid cells impacts reef-builder effect sizes in largely the same way it does for bioturbators. Again, this does not account for the persistence of strong effect sizes throughout the Phanerozoic, even if (as with the within-palaeoenvironment trends) the stage-by-stage trends are slightly different.

Why the different spatial threshold approaches impact effect sizes differently is not clear, and a true sensitivity test that tests the occurrence or collection threshold and the size of equal area grid cells is unfortunately out of scope for this study (as one of these effect size analyses typically takes up to 24 hours of computation time, and would be unfeasible for this revision).

We have included these figures in the supplemental information, and added the following text to the manuscript:

"To test the impacts of undersampled regions, we also conducted a set of effect size analyses on a dataset in which data from formations and equal area grid cells that do not contain 20 occurrences in each stage were removed from the dataset prior to analysis. Although we find some stage-by-stage differences in some threshold tests, this protocol has minimal statistical impact on broad Phanerozoic results (Supplementary Figure 11)." Methods: Subsampling protocol, lines 483-488.

Finally, thank you for your reviews! They have certainly improved the statistical robustness of the study and the overall manuscript.

Review of Marine Phanerozoic biodiversity increased in presence 1 of ecosystem engineers (NCOMMS-24-79000-T) by Cribb et al.

General comments:

Thank you for inviting me to review this manuscript and please accept my apologies for my delayed response. If you have any questions about any of my comments, I will endeavour to reply right away to speed up the rest of the review process.

This paper by Cribb and colleagues describes the results of an exciting new set of analyses using a refined version of the PBDB (the standard for such analyses) which demonstrate that ecosystem engineers, specifically reef-builders and bioturbators, have a significant impact on the taxonomic diversity of the geological formations they originate from across the Phanerozoic. As far as I am aware, the results of such ambitious analyses have never been published before, which would make this paper a worthy addition to the *Nature Communications* Portfolio. I would like to congratulate the authors for their excellent writing, clear figures, and overall great work.

I only have two issues with the paper as currently written that require addressing before I can recommend it for acceptance for publication (the rest of my comments below are very minor). These are outlined in detail below but in summary they are:

1. Whether the within-formation subsampling the authors use mitigates the spatial biases in the PBDB as well as the more common practice of geographic grid cell subsampling,

2. Whether the variation in palaeoenvironment composition through time between the formations that have ecosystem engineers and those that don't is having an impact on effect size.

If the authors have any questions about how best to approach this, I am happy for them to get in touch with me directly, as I think this will be a very impactful study when published.

Best wishes,
Tom Smith
Department of Earth Sciences
University of Oxford

Specific comments:
Introduction:
Line 36: I don't think "Diversity has been generally increasing" encompasses the full spectrum of recent results. Close et al's (2020, *Science*, reference 15) spatially standardised analyses recover a noisy pattern with comparable spikes throughout the Phanerozoic. Given the rest of the paragraph focusses on the first key feature (that diversity rises and falls), maybe tweak this sentence to reflect that perceptions of diversity depend on how data are standardised or something else?

Lines 50-53: A bit of a pedantic point (sorry!) but I think it is important to explicitly state that ecosystem engineers differentiate themselves from other organisms in that they change the resource availability for other organisms, not just themselves. Maybe just add 'for other organisms' to the end of the sentence?

Line 64: typo? "key drivers of increasing biodiversity"?

Results – effects on Phanerozoic biodiversity:
Lines 93-95: reference to Figures 1 and 2 needed.

95-96: I think it could be worth defining weak, significant, and strong effect sizes up top. Perhaps "These associations are reflected in strong (g≥0.8 with non-overlapping uncertainty, significant (g≥0.5), and weak (g≥0.2) positive effect sizes? I know this is in the Methods of the paper but I think having it here will help.

Lines 96-99: I appreciate the author's candour here and this is a completely subjective opinion (so please feel free to ignore) but I feel qualifying statements (especially those referring the supplementary figures) are best saved for the end of the sections they pertain to. I'd suggest moving these lines to the end of the subsection, so readers have a better idea as to what they pertain to.

Lines 102-105: Similar to above, I feel pointing readers to supplemental figures showing the impact of palaeoenvironment before digging into the main data figures is a bit confusing. I'd recommend moving this aside to the end of the paragraph.

Line 114: maybe quantify the difference between effect sizes required between a pair of stages for the latter to be considered notable?

Line 125: reference to Figures 2 needed.

Lins 129-131: similar to the paragraph about bioturbators, I'd move the nod to the supplemental results looking at the impact of palaeoenvironment to the end of the paragraph.

Results – effects during mass extinctions:
Lines 149-150: I think the description of the effect sizes of bioturbators across the end-Ordovician mass extinction needs changing to reflect the crash before the boundary and steady increase across it.

Line 164: "significant moderately positive" is a bit confusing. Significant positive is enough if you explicitly specify what each effect size category is comprised of at the beginning of the Results.

General: I wonder if there is a way this section can be re-written to make it less repetitive and more concise. This is just a suggestion, so please feel free to ignore, but I think these results would be much easier to digest if you discussed bioturbators and reef-builders separately and didn't describe each curve so granularly (the figures are clear enough to make such descriptions unnecessary in my opinion). For each, you could state over which boundaries each of them increases/decreases/stays the same and then wrap up with the summary.

Discussion:
General: I think the discussion is well-written and doesn't overreach. However, I do think some space needs to be given to a discussion about the interaction between time, environment (shallow versus deep, carbonate versus siliciclastic), and effect size. Looking at supplementary figures 3 and 5 (which I feel belong in the main text, personally), formations with bioturbators and without reef-builders seem to be split 50-50 between siliciclastic and carbonate sediments in more stages than those with reef builders and without bioturbators (which seen to lean carbonate). This strikes me as something that could impact the analyses and needs addressing in some way. Below I suggest fitting a GLMM to the data but I am open to other solutions.

Line 246: “reef-builder” missing ‘s’.

Line 343: “more particularly” maybe drop the more?

Methods – subsampling protocol:
General: I think I understood what the authors did here (first sampling 700 fossil occurrences for each stage, then from all the formations represented within this 700-occurrence sample, sampling 20 occurrences of each) but it’s unclear. Was the random sampling of the formation occurrences conducted with replacement within each time bin? A bit of extra detail would be great.

Within-formation subsampling: While I think the authors protocol is effective in terms of standardising the number of occurrences per formation, I am unsure as to how effective it is at controlling for the impact of the species-area effect, given that geological formations vary in the size of the geographic areas they preserve. Therefore, I am a little concerned that there may still be spatial biases lurking in the results.

This is easily addressed, however! The authors could add of a rasterization step into the subsampling pipeline after time-binning the data but before standardising the number of occurrences per formation. This would necessitate the dropping of the stage-level subsampling but, given that the number of occurrences will be standardised by later steps in the pipeline, I don’t think this will be an issue.

Alternatively, they could run a sensitivity test that swaps the within-formation subsampling for a within-grid-cell subsampling alternative (again, it would likely become necessary to drop the stage subsampling step) and then compares the results of the two for differences. Either way, I’d like to see this tested.

Methods – effect size analysis:
General: is there enough data to fit a/some GLMM(s)? If possible, I think it would strengthen the results of this study to be able to see the variance in Shannon’s Diversity Index apportioned to ecosystem engineer diversity while controlling for the impact of global average temperature, water depth, and palaeoenvironment. From the looks of Supplementary Figures 3 and 5, the latter two may have an impact which is currently unaccounted for.

A GLMM approach would also allow you to model geographic location of samples as a random effect if you rasterised the data as suggested above. I should stress that this is not something that I think is mandatory – the only aspect of this that I think *needs* addressing is the role palaeoenvironment plays.

Line 464: I actually think Supplementary Figures 3 and 5 (maybe 4 and 6 as well) belong in the main text, perhaps combined into a single figure somehow. Just a personal opinion but I think there’s something to them worth discussing (see above).

Methods – climactic and evolutionary correlates:
General: as with above, I think there is a place for rasterization in this workflow to ensure spatial biases aren’t compromising these results.

Figures:
Figure 3: I’d suggest standardising either the duration of the time series or the number of sampled points across the 5 panels. At the moment, comparisons between the 5 are hindered by the differences in the above. Also, can you shrink the y-axis to maybe -1.25 – 1.25? Will make it easier to view changes in effect size through time, especially if 0 is at the midline.

Figure 4. Panel B y-axis label cut off.

Figure 6: Panels A & C,I like the colours but personally, think I'd find it easier to track the relationship between temperature and the effect size if average temperature was plotted as a separate curve. Just a thought!
